# Acute effects of moderate vs. vigorous endurance exercise on urinary metabolites in healthy, young, physically active men—A multi-platform metabolomics approach

**DOI:** 10.3389/fphys.2023.1028643

**Published:** 2023-01-30

**Authors:** Sina Kistner, Carina I. Mack, Manuela J. Rist, Ralf Krüger, Björn Egert, Nathalie Biniaminov, Ann Katrin Engelbert, Stephanie Seifert, Claudia Dörr, Paola G. Ferrario, Rainer Neumann, Stefan Altmann, Achim Bub

**Affiliations:** ^1^ Department of Physiology and Biochemistry of Nutrition, Max Rubner-Institut, Karlsruhe, Germany; ^2^ Department of Safety and Quality of Fruit and Vegetables, Max Rubner-Institut, Karlsruhe, Germany; ^3^ Institute of Sports and Sports Science, Karlsruhe Institute of Technology, Karlsruhe, Germany; ^4^ TSG ResearchLab gGmbH, Zuzenhausen, Germany

**Keywords:** exercise metabolomics, continuous physical exercise, exercise intensity, urine metabolome, metabolic markers, NMR, LC-MS, GC×GC-MS

## Abstract

**Introduction:** Endurance exercise alters whole-body as well as skeletal muscle metabolism and physiology, leading to improvements in performance and health. However, biological mechanisms underlying the body’s adaptations to different endurance exercise protocols are not entirely understood.

**Methods:** We applied a multi-platform metabolomics approach to identify urinary metabolites and associated metabolic pathways that distinguish the acute metabolic response to two endurance exercise interventions at distinct intensities. In our randomized crossover study, 16 healthy, young, and physically active men performed 30 min of continuous moderate exercise (CME) and continuous vigorous exercise (CVE). Urine was collected during three post-exercise sampling phases (U01/U02/U03: until 45/105/195 min post-exercise), providing detailed temporal information on the response of the urinary metabolome to CME and CVE. Also, fasting spot urine samples were collected pre-exercise (U00) and on the following day (U04). While untargeted two-dimensional gas chromatography-mass spectrometry (GC×GC-MS) led to the detection of 608 spectral features, 44 metabolites were identified and quantified by targeted nuclear magnetic resonance (NMR) spectroscopy or liquid chromatography-mass spectrometry (LC-MS).

**Results:** 104 urinary metabolites showed at least one significant difference for selected comparisons of sampling time points within or between exercise trials as well as a relevant median fold change >1.5 or <0. $\bar{6}$ (NMR, LC-MS) or >2.0 or <0.5 (GC×GC-MS), being classified as either *exercise-responsive* or *intensity-dependent*. Our findings indicate that CVE induced more profound alterations in the urinary metabolome than CME, especially at U01, returning to baseline within 24 h after U00. Most differences between exercise trials are likely to reflect higher energy requirements during CVE, as demonstrated by greater shifts in metabolites related to glycolysis (e.g., lactate, pyruvate), tricarboxylic acid cycle (e.g., *cis*-aconitate, malate), purine nucleotide breakdown (e.g., hypoxanthine), and amino acid mobilization (e.g., alanine) or degradation (e.g., 4-hydroxyphenylacetate).

**Discussion:** To conclude, this study provided first evidence of specific urinary metabolites as potential metabolic markers of endurance exercise intensity. Future studies are needed to validate our results and to examine whether acute metabolite changes in urine might also be partly reflective of mechanisms underlying the health- or performance-enhancing effects of endurance exercise, particularly if performed at high intensities.

## 1 Introduction

Continuous endurance exercise is known to play an important role in the general human population, leading to improvements of cardiorespiratory fitness, metabolic adaptations, and reductions in disease risks (Egan and Zierath, 2013). For adults, at least 150–300 min of moderate-intensity or 75–150 min of vigorous-intensity aerobic physical activity are recommended per week (Bull et al., 2020). Yet, biological mechanisms involved in mediating the body’s adaptation to acute and chronic physical activity are still insufficiently understood (Zierath and Wallberg-Henriksson, 2015). Metabolomics has the potential to simultaneously analyze a wide range of metabolites (Bujak et al., 2015), hence allowing to systematically examine exercise-related changes in human metabolism and to hint at metabolic markers or pathways associated with the health- or performance-enhancing effects of physical activity (Kelly et al., 2020; Belhaj et al., 2021). In the context of endurance exercise, metabolomics can help to distinguish metabolic alterations in response to exercise protocols varying with regard to intensity, duration, and frequency in different populations (Duft et al., 2017) and, finally, to define appropriate exercise regimens for obtaining specific effects on metabolism (Heaney et al., 2017).

During the last decade, metabolomics has been increasingly applied to study the effects of acute endurance exercise on the human metabolome. Most studies focused on continuous exercise at either moderate (Manaf et al., 2018) or vigorous (Enea et al., 2010; Krug et al., 2012; Nieman et al., 2017; Davison et al., 2018) intensity, (sub-)maximal varying intensity (Pohjanen et al., 2007; Chorell et al., 2012; Muhsen Ali et al., 2016; Stander et al., 2018; Nayor et al., 2020; Shi et al., 2020), or incremental intensity until exhaustion (Lewis et al., 2010; Mukherjee et al., 2014; Kistner et al., 2020; San-Millán et al., 2020; Schranner et al., 2021). Other studies compared different exercise parameters, e.g., continuous vs. interval and/or resistance exercise (Peake et al., 2014; Zafeiridis et al., 2016; Siopi et al., 2017; Siopi et al., 2019; Morville et al., 2020), or 120-min moderate vs. 60-min vigorous endurance exercise (Lehmann et al., 2010) by using either a crossover or, as in the latter case, a parallel study design. Many metabolites showing a global increase in response to endurance exercise were related to lipid metabolites, e.g., glycerol (Pohjanen et al., 2007; Lewis et al., 2010; Zafeiridis et al., 2016; Manaf et al., 2018; Stander et al., 2018; Shi et al., 2020), long-chain fatty acids (Nieman et al., 2017), or acylcarnitines (Lehmann et al., 2010; Krug et al., 2012; Nieman et al., 2017). Further exercise-related changes have been documented for e.g., lactate and pyruvate (Lewis et al., 2010; Mukherjee et al., 2014; Zafeiridis et al., 2016), tricarboxylic acid (TCA) cycle intermediates (Peake et al., 2014; Nieman et al., 2017; Stander et al., 2018; Shi et al., 2020), or ketone bodies (Lewis et al., 2010; Stander et al., 2018), reflecting alterations in energy-producing metabolic pathways. Even if those study results are not directly comparable due to discrepancies with regard to exercise protocols, included participants, collected biospecimens, sampling times, and applied analytical techniques, it can be deduced from the current state of research that acute endurance exercise of lower duration and intensity leads to rather modest shifts in post-exercise metabolite concentrations compared to more intensive and prolonged endurance exercise (Kelly et al., 2020). However, to the best of our knowledge, no metabolomics study has until now evaluated differences in the acute metabolic response to continuous moderate exercise (CME) or continuous vigorous exercise (CVE) of equal duration within the same study cohort. Although blood has been the most widely used biofluid in metabolomics research so far (Bujak et al., 2015), recent studies support the utility of urine to reflect acute exercise-induced metabolic alterations, e.g., after (sub-)maximal endurance exercise, as demonstrated by changes in metabolites related to glycolysis (e.g., lactate, pyruvate) or amino acid (AA) metabolism (e.g., alanine, taurine) (Mukherjee et al., 2014; Muhsen Ali et al., 2016; Siopi et al., 2017; Kistner et al., 2020). Yet, those studies were restricted to a single analytical technique, i.e., either targeted nuclear magnetic resonance (NMR) (Mukherjee et al., 2014; Kistner et al., 2020) or targeted liquid chromatography-mass spectrometry (LC-MS) (Muhsen Ali et al., 2016; Siopi et al., 2017) analysis, and detected a rather small number of urinary metabolites, ranging from 32 (Mukherjee et al., 2014) to 64 (Siopi et al., 2017). A further limitation in two of the mentioned studies is that only single pre- and post-exercise urine samples were collected, respectively (Mukherjee et al., 2014; Kistner et al., 2020), impeding to obtain time-resolved information on metabolite alterations.

Intending to address several of the above described research gaps in acute endurance exercise-related metabolomics studies and to provide a comprehensive overview of post-exercise urinary metabolite changes that are dependent on exercise intensity, we conducted a randomized crossover intervention study under controlled laboratory conditions. Since knowledge on specific biomarkers for different endurance exercise protocols in easily obtainable biofluids is rather scarce, the aim of this study was to investigate the effects of 30-min CME (at 50% of peak oxygen uptake (VO_2peak_)) and CVE (at 75% VO_2peak_) on the urinary metabolome of 16 healthy, young, and physically active men. Urine was chosen as a biospecimen because it is easily accessible, stable, and under less homeostatic regulation than other biofluids (Bouatra et al., 2013; Wu and Gao, 2015). By applying a multi-platform metabolomics approach (i.e., targeted NMR and LC-MS and untargeted two-dimensional gas chromatography-mass spectrometry (GC×GC-MS)), a broad coverage of the urinary metabolome was obtained. While targeted analyses allowed the absolute quantification of known metabolites, untargeted analyses additionally permitted to detect previously unknown, but potentially important exercise-related metabolic markers. In more detail, we aimed to detect urinary metabolites responsive to CME and/or CVE (i.e., *exercise-responsive* metabolites) as well as urinary metabolites that showed intensity-dependent differences in the post-exercise response between CME and CVE (i.e., *intensity-dependent* metabolites). This study was characterized by highly standardized experimental procedures, e.g., controlled diets on the days before and during intervention, individualized exercise protocols, and clearly defined post-exercise urine collection phases, thus taking potential confounding factors into account and ensuring to obtain temporal information on post-exercise urinary metabolite alterations. A further advantage of the present study is that absolute and cumulative excretion of metabolites from targeted approaches could be calculated owing to complete urine collection and determination of urine volume in each post-exercise sampling phase.

## 2 Materials and methods

### 2.1 Participants and study design

**FIGURE 1:**
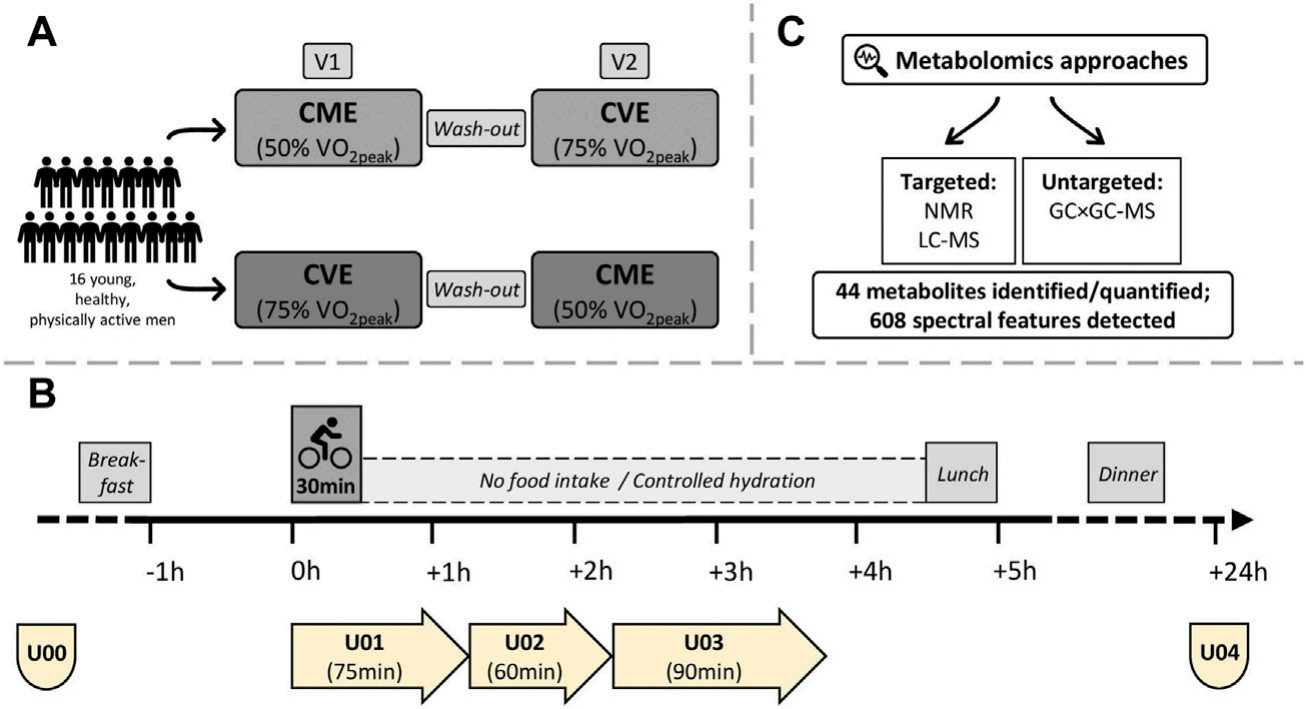
Study design, urine sample collections, and metabolomics approaches. **(A)** Participants were randomized to 30 min of CME or CVE at V1 or V2, respectively. **(B)** Fasting spot urine samples were obtained in the morning of V1 and V2 (U00) and after 24 h (U04). Three urine collection phases covered the (post-)exercise periods (U01: urine produced during exercise trials until 45 min post-exercise; U02: urine produced from 45 to 105 min post-exercise; U03: urine produced from 105 to 195 min post-exercise. **(C)** 44 metabolites were identified and quantified by targeted NMR or targeted LC-MS. 608 spectral features were detected by untargeted GC×GC-MS.

Seventeen young, healthy, and physically active men took part in this acute exercise trial conducted at the Division of Human Studies at the Max Rubner-Institut in Karlsruhe, Germany. One participant was excluded from data analysis (see Section 2.7), therefore, the final sample size was *n* = 16. All participants were non-smokers, participated in regular physical training, and provided written informed consent before entering the study. Inclusion and exclusion criteria are listed in Supplementary Table S1. By using a crossover design, participants completed two 30-min endurance exercise trials at either moderate (50% VO_2peak_) or vigorous (75% VO_2peak_) intensity, which were scheduled at least 1 week apart. After preliminary examinations (see Sections 2.2, 2.3), participants were randomly assigned to the CME or CVE intervention at visit 1 (V1) or visit 2 (V2), respectively (Figure 1A).

### 2.2 Anthropometry, body composition, and clinical examinations

Before study onset, body weight and height were measured in underwear and without shoes (Seca 285, Hamburg, Germany), and body mass index was calculated. Additionally, body composition was assessed by bioelectrical impedance analysis (BIA Nutriguard MS, Data Input, Pöcking, Germany). Lean body mass and fat mass were determined with the software NutriPlus Version 5.3.0. Percentage fat mass was calculated by dividing total fat mass by total body weight and multiplying by 100. Clinical parameters like resting heart rate (HR_rest_), systolic and diastolic blood pressure were measured after a resting period of at least 5 min in a sitting position (Boso Carat Professional, Bosch & Sohn, Jungingen, Germany).

### 2.3 Preliminary exercise testing

To establish individual protocols for the acute exercise trials, participants performed a cardiopulmonary exercise test on a bicycle ergometer (Excalibur Sport Ergometer, Lode B.V., Groningen, Netherlands). Briefly, each participant started cycling at 50 W and workload was then augmented by 50 W every 3 min until individual exhaustion. During the test, a pedal frequency of 60–90 revolutions per minute had to be maintained. Respiratory gas exchange was measured by using a portable spiroergometry system with breath-by-breath technology (MetaLyzer 3B, Cortex, Leipzig, Germany) and heart rate (HR) was continuously recorded (Polar H7, Polar, Kempele, Finland). All participants reached a respiratory exchange ratio >1.1, which stands for a strong exhaustion criterion (Löllgen and Leyk, 2018). Main endpoint of this test was VO_2peak_, i.e., the highest achieved oxygen uptake. To determine the workloads at which participants had to perform the 30-min CME or CVE, respectively, the power at either 50% or 75% of the individual’s VO_2peak_ was calculated. Further assessed measures were maximal power, maximum heart rate (HR_max_), and power at the individual anaerobic threshold (P_IAT_). The P_IAT_ was determined with the Ergonizer software (Ergonizer, Version 5.0, Freiburg, Germany) after having measured lactate (EKF Biosen C-Line, EKF Diagnostics GmbH, Barleben, Germany) in capillary blood samples taken from earlobes at the end of each incremental stage. If blood pressure had been slightly elevated in the resting state, it was also controlled during bicycle ergometry (boso Carat professional, Bosch + Sohn, Jungingen, Germany). According to national ergometry standards, certain break-off criteria, e.g., systolic blood pressure >230 mmHg or diastolic blood pressure >115 mmHg, angina pectoris symptoms, or severe dyspnea, were applied.

### 2.4 Controlled diet and hydration

On both exercise trial days as well as on each preceding day, participants were instructed to follow a prescribed standardized diet. The personalized dietary plans were designed by a qualified dietician by using DGExpert Version 1.9.1 (Deutsche Gesellschaft für Ernährung, Bonn, Germany), and aimed at filling body energy reserves adequately. A carbohydrate-rich mixed diet was chosen, providing about 55%–60% of the energy from carbohydrates, 30% from fat, and 10%–15% from protein (Supplementary Table S2). The foods included in the dietary plans were provided by the study center, ensuring that food selection was identical for all participants. To estimate the particular amounts of foods that should be consumed based on individual energy needs, basal metabolic rates were calculated by using a formula that has been generated based on data from the cross-sectional Karlsruhe Metabolomics and Nutrition study [*n* = 301; *R* ^2^ = 0.77 (Armbruster et al., 2018)]: Basal metabolic rate = 21.14 × lean body mass (kg) + 345.5 (previously unpublished formula). Basal metabolic rates were then multiplied by a physical activity factor of 1.8, reflecting the moderate to high activity level of participants (FAO/WHO/UNU, 2004). To minimize intra-individual variance between exercise trials, the personalized dietary plans were identical with regard to food quality for both visits and each preceding day. Besides, participants were instructed to drink water and apple juice and to refrain from intense exercise on the days before both exercise trials. Discrepancies between estimated and actual food consumption have not been documented, since the primary aim was to fill body energy reserves.

With regard to breakfast on both intervention days, the individual energy intake was designed to meet at least the energy expenditure in the particular exercise trials, which has been calculated based on spiroergometry data from preliminary exercise testing. The standardized breakfast included foods and drinks contained in the dietary plans (e.g., baguette rolls, butter, cheese, honey, jam, tea, water) and had to be finished approximately 1 h before the exercise trial started. If participants consumed more or less than the calculated minimum amounts of different foods, discrepancies were recorded. The actual energy and macronutrient intake at breakfast is provided in Supplementary Table S2. Hydration was also controlled during both exercise trials, i.e., the individual water intake was recorded until 195 min post-exercise (Supplementary Table S2).

### 2.5 Exercise interventions and urine sample collection

On both intervention days, participants arrived in the study center at 07.30 a.m. after an overnight fast and immediately provided a fasting spot urine sample (U00; pre-exercise) (Figure 1B). Ninety minutes before starting the exercise trials, participants consumed a standardized breakfast (see Section 2.4), and were then instructed to empty their bladders. Both exercise interventions took place between 09.00 and 10.00 a.m. According to the Practice Guidelines for Exercise Testing (Wonisch et al., 2008), standardized environmental conditions (i.e., ambient temperature and humidity) were maintained in the testing room. The ergometer saddle was set at the same height for both interventions. After a 2-min warm-up at 50 W (CME trial) or 100 W (CVE trial), participants performed their individual 30-min exercise protocols on a bicycle ergometer (SRM Sport High Performance, SRM, Jülich, Germany) at either moderate (50% VO_2peak_) or vigorous (75% VO_2peak_) intensity. During the exercise trials, a pedal frequency of 60–90 revolutions per minute had to be maintained. After having completed the acute exercise protocols, participants kept cycling at 50 W for 3 min (CME trial) or at 100 W for 1 min and 50 W for 2 min (CVE trial) to cool down.

The post-exercise urine sampling was divided into three collection phases (Figure 1B). While the first collection phase (U01) comprised the urine produced during each acute exercise trial and the following 45 min, the second (U02) and third (U03) collection phases covered the urine produced from 45 to 105 min and from 105 to 195 min post-exercise, respectively. At the end of each collection phase, participants were asked to empty their bladders. The post-exercise period (i.e., from the end of the acute exercise trial until lunch) was mainly spent in a sitting position and participants were instructed not to eat or drink anything but water. During both interventions until lunch, HR (Polar H7, Polar, Kempele, Finland) was continuously monitored. Finally, another fasting spot urine sample (U04; 24 h after U00) was provided from all participants in the morning after each intervention day. Fasting spot urine samples (U00, U04) were collected into polypropylene collection cups (Sarstedt AG & Co., Nümbrecht, Germany), immediately placed on ice, transferred into 15 mL tubes, and then centrifuged at 1850 g at 4°C for 10 min. Urine obtained in the post-exercise phases (U01, U02, and U03) was collected in 2 L urine containers (Sarstedt AG & Co., Nümbrecht, Germany), which were stored in styrofoam boxes filled with ice. After urine collections, urine volume was determined, samples were transferred into 15 mL tubes, and centrifuged at 1850 g at 4°C for 10 min. All urine samples were separated into aliquots and cryopreserved at −196°C until analyses. Osmolality of urine samples was determined by freezing point depression using an Advanced Instrument Micro-Osmometer model 2020 (Norwood, MA, United States).

### 2.6 Metabolomics analyses

To obtain a broad coverage of the urinary metabolome, three different analytical techniques were combined, namely targeted NMR and LC-MS as well as untargeted GC×GC-MS (Figure 1C). In a complementary manner, these three methods detected metabolites of different chemical classes and at different concentration ranges. NMR is suitable for detecting metabolic compounds in the milli-to micromolar range, with the advantage of being a relatively robust, rapid, and inherently quantitative method (Emwas, 2015). Hence, it was applied in this study to identify and quantify metabolites with comparatively high urinary concentration levels, such as several organic acids (e.g., lactate, pyruvate), AAs (e.g., alanine, glycine, threonine), or purine derivatives (e.g., hypoxanthine). Compared to NMR spectroscopy, MS-based techniques offer higher sensitivity and selectivity (Johnson et al., 2016), and allow to detect complementary analyte classes with different polarity and volatility (Lei et al., 2011). While LC-MS analysis was applied to add several methylated amino compounds (e.g., betaine, choline, dimethylamine, methylhistidines) to the targeted approach, untargeted GC×GC-MS analysis additionally provided semi-quantitative data of a wide range of urinary metabolites, including AAs (e.g., glutamic acid, tryptophan), organic acids (e.g., malic acid), phenolic acids (e.g., hippurate), sugars and derivatives (e.g., fructose, mannose, lactose), and several unidentified metabolites.

#### 2.6.1 NMR (targeted approach)

All urine samples were analyzed by 1D-^1^H-NMR spectroscopy, as previously described (Rist et al., 2017; Kistner et al., 2020). Briefly, samples were centrifuged and supernatants were mixed with 10% of a buffer containing 1.5 M KH_2_PO_4_, 2 mM NaN_3_, and 5.8 mM TSP in D_2_O at pH 7.4 in 5 mm NMR tubes (Duran, purchased from Roth GmbH & Co. KG, Karlsruhe, Germany). Samples were measured at 300 K on a Bruker 600 MHz spectrometer [AVANCE II with 1H-BBI room temperature probe (Bruker BioSpinGmbH, Rheinstetten, Germany)] equipped with a BACS sample changer. Quality control (QC) samples were prepared by pooling urine samples from all participants. On each measurement day, at least five QC samples were analyzed along with approximately 80 urine study samples, ensuring the comparability of the spectra over time. The identification and quantification of 54 metabolites was carried out with the Chenomx NMR Suite 8.4 (Chenomx, Edmonton, AB, Canada). Of those metabolites, 33 could be identified and quantified with sufficient confidence and were considered for further analyses. The remaining 21 metabolites were excluded, either due to low validity of peak annotation and/or quantification in study samples [e.g., due to high urine dilutions in post-exercise samples, i.e., U01, U02, U03 (*n* = 18), or small concentration ranges independent of sample osmolality, possibly due to high volatility of substances during sample preparation (*n* = 3)]. In the latter case, subsequent normalization to osmolality would have led to systematic errors in urinary metabolite concentrations.

#### 2.6.2 LC-MS (targeted approach)

Urine samples were also analyzed by targeted ultra-performance liquid chromatography-tandem mass spectrometry, using an Acquity H-Class UPLC coupled to a Xevo TQD triple quadrupole MS (both from Waters, Eschborn, Germany), as previously described (Kistner et al., 2019; Roggensack et al., 2020). Briefly, samples were diluted 25 times with eluent A (1:1 acetonitrile/aqueous 50 mM ammonium formate) and separated by an inverse acetonitrile gradient on a polar HILIC column (Acquity BEH Amide, 100 × 2.1 mm, 1.7 µm, Waters, Eschborn, Germany). Analytes were detected by positive ESI and timed MRM. For quantification, matrix-matched calibrators and QCs (3 levels), dedicated isotope-labelled internal standards, and standard addition were used. Of 29 detected analytes, 19 were quantified with sufficient confidence and included into further analyses. The remaining 10 analytes were excluded due to systematic interferences, attributed to exceptional urine dilution particularly during the post-exercise period. This led to low signal intensities in combination with enhanced background, preventing further evaluation of affected analytes. For two included metabolites (sarcosine, hydroxyproline), few samples had concentrations below the limit of quantification, i.e., signal-to-noise ratio 10:1. However, these values were not excluded from statistical calculations, since they represent at least a semi-quantitative number for a low background, thus avoiding missing values.

In total, 52 metabolites from both targeted approaches were eligible for data analysis. Of those, eight (β-aminoisobutyrate, betaine, carnitine, dimethylamine, *N*,*N*-dimethylglycine, histidine, trigonelline, trimethylamine *N*-oxide) were accessible by both NMR and LC-MS (method correlation coefficients: *R* ^2^ > 0.87). Since LC-MS offers a higher sensitivity than NMR and peak assignment as well as integration in NMR is sometimes critical and pH-dependent (Tebani et al., 2018), we only considered those values obtained by LC-MS for further data handling and statistical analyses. Finally, quantitative data from 44 different metabolites (25 NMR, 19 LC-MS) were combined into a common data matrix. For most included metabolites, imprecision was <15%. Before statistical analysis, two normalization approaches to control for variations in urine dilution were applied: For both spot urine samples (U00 and U04) and urine samples collected in the three post-exercise periods (U01, U02, and U03), metabolite concentrations were normalized to osmolality. In Supplementary Table S3, data on osmolality-normalized urinary concentrations for the 44 metabolites from targeted approaches are presented along with data for relevant GC×GC-MS metabolites, see Section 2.7.2. To additionally calculate absolute metabolite excretion in urine sample collection phases (U01, U02, and U03), respective urinary metabolite concentrations were multiplied by urine volume. The sum of absolute metabolite excretions during U01, U02, and U03 was used to estimate the cumulative metabolite excretion in the post-exercise period. In Supplementary Table S4, additional data on both absolute and cumulative excretion for the 44 metabolites from targeted approaches are provided.

#### 2.6.3 GC×GC-MS (untargeted approach)

Spot and collected urine samples were analyzed using a Shimadzu Corp. GC×GC-MS system with a common non-polar × medium polar column combination, as previously described (Vázquez-Manjarrez et al., 2019). Sample preparation was performed as described by Vázquez-Manjarrez et al. Briefly, urine samples were diluted according to osmolality (60 mOsm/kg), evaporated, and then derivatized in a two-step procedure. First, a methoximation using a methoxylaminhydrochloride solution (20 mg/mL in pyridine) was performed, followed by a trimethylsilylation using *N*-methyl-*N*-(trimethylsilyl)-trifluoroacetamide. Details of the GC×GC-MS analysis and processing of the data are described in Supplementary Data Sheet S1. A QC sample based local linear regression function was used to remove slight drift and offset effects (Egert et al., 2015). After automated data processing, 608 spectral features were detected and considered for further analysis.

### 2.7 Data handling and statistical analysis

We excluded one participant due to extremely high urine osmolality, suggesting a previously unknown disorder of water and electrolyte balance. Descriptive characteristics for the remaining 16 participants are presented as means and standard deviations, and differences between CME and CVE trials were examined by paired Wilcoxon signed-rank tests. Regarding metabolomics analyses, data handling and statistics were conducted separately for quantitative NMR and LC-MS data (targeted approach) and relative quantitative GC×GC-MS data (untargeted approach).

**FIGURE 2:**
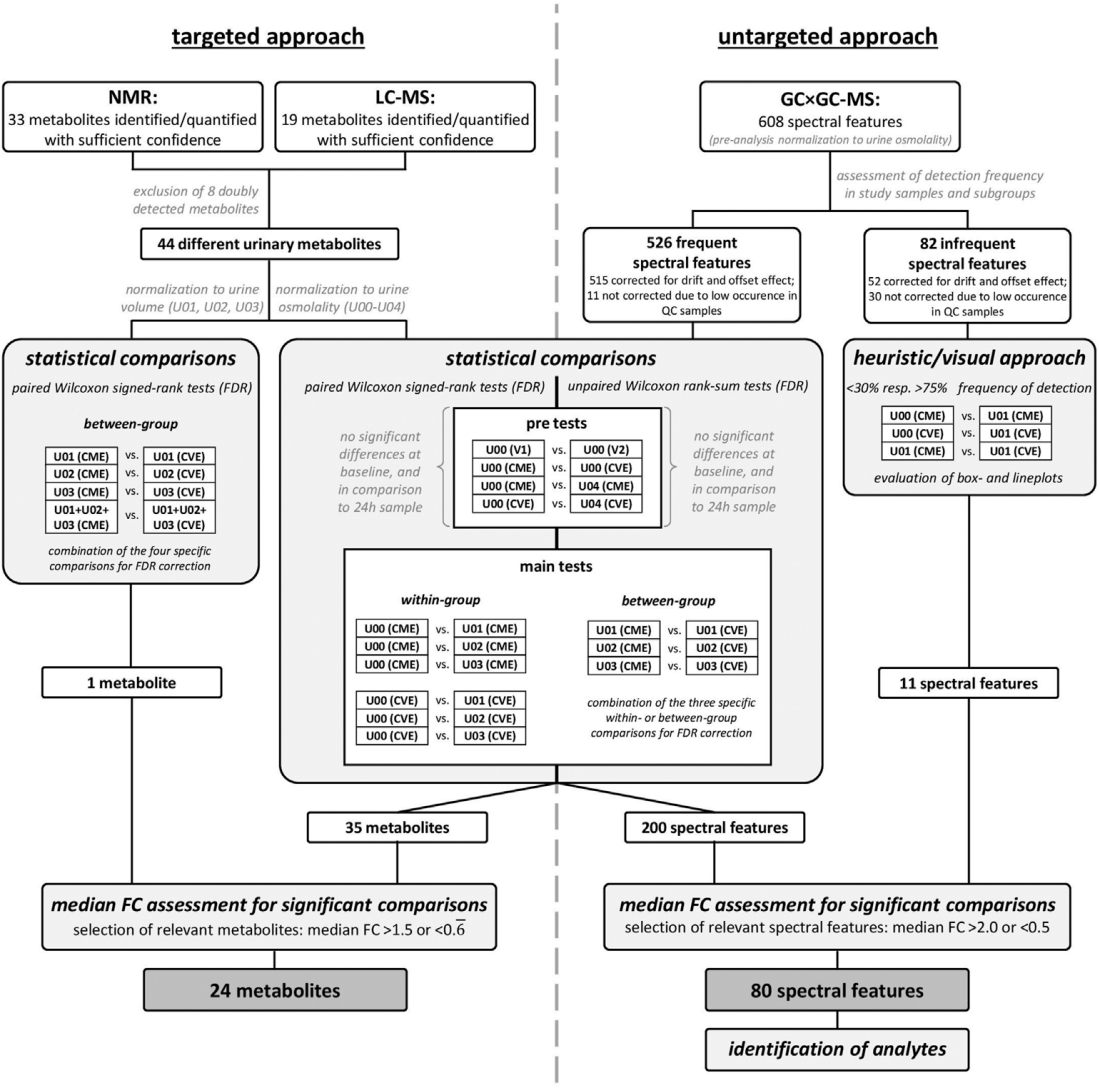
Workflow for metabolomics and statistical analyses.

Briefly, as depicted in Figure 2, four pre-tests were performed to ensure that baseline levels (U00) of urinary metabolites were comparable between V1 and V2 or the CME and CVE trial, respectively, and that baseline (U00) and end levels (U04) of urinary metabolites were comparable within the CME or CVE trial, respectively. Selected main comparisons were conducted to examine the effects of either the CME or CVE trial on urinary metabolites (*within-group* comparisons: U00-U01; U00-U02; U00-U03), or to examine differences in post-exercise urinary metabolites between the CME and CVE trial (*between-group* comparisons: U01-U01; U02-U02; U03-U03). Based on histograms and QQ-normal plots of metabolite concentrations and absolute metabolite excretions, normal distribution cannot be assumed. In fact, most distributions were skewed and the data points in the QQ-plots did not lie on a straight diagonal line. Therefore, non-parametric statistical analysis was conducted. The level of statistical significance for all comparisons was set at *α* = 0.05 after correcting for multiple testing. Statistical analysis und figure generation were performed using SAS JMP 16.1.0 (SAS Institute Inc. 2013, Cary, NC, United States).

#### 2.7.1 Targeted approach

For the 44 metabolites detected by targeted approaches, pre-tests as well as main comparisons were performed by using paired Wilcoxon signed-rank tests (Figure 2). The false discovery rate (FDR) correction for multiple testing was applied, separately for each pre-test and combined for the specific *within-* or *between-group* comparisons, respectively. While pre-tests and *within-group* comparisons were performed on osmolality-normalized metabolite concentrations, *between-group* comparisons were additionally conducted based on absolute and cumulative metabolite excretions. Relevant metabolites were selected based on an FDR-corrected *p*-value <0.05 and a median fold change (FC) >1.5 or <0. $\bar{6}$ .

#### 2.7.2 Untargeted approach

The 608 spectral features were included for further evaluation in a two-step approach (Figure 2). For this, the spectral features were separated based on their frequency of detection in each of the different urine samples for each intervention (i.e., subgroups, such as U00 (CME), U00 (CVE), etc.); only spectral features with more than 50% frequency in at least nine of the ten subgroups were considered for statistical analysis. This resulted in 526 spectral features for the statistical analysis, while the remaining 82 spectral features were evaluated based on a heuristic and visual approach. Due to a problem during the first two measurement days, 22 study samples had to be excluded from evaluation. As a result, a pairwise strategy was no longer possible without further loss of statistical power, therefore, unpaired, non-parametric Wilcoxon tests with FDR correction for multiple testing were performed for selected comparisons. The most relevant spectral features were selected based on a median FC >2.0 or <0.5. The remaining 82 less frequent spectral features were evaluated based on substantial differences in the frequencies of detection in selected subgroups (Figure 2). Spectral features with less than 30% and more than 75% frequency of detection and at the same time a median FC >2.0 or <0.5 for selected comparisons of subgroups were evaluated by box- and lineplots. Visual analysis was performed to ensure that remaining spectral features were interesting and no random effects occurred.

Due to differences in the analytical precision of the applied methods, different boundaries for relevant FCs were selected. Thus, metabolites with a median FC >1.5 or <0. $\bar{6}$ (for quantitative NMR and LC-MS data) and >2.0 or <0.5 (for relative quantitative GC×GC-MS data) were considered relevant and defined as *exercise-responsive* (for *within-group* comparisons) or *intensity-dependent* (for *between-group* comparisons). With respect to GC×GC-MS data, only metabolites selected as relevant after statistical and heuristic approaches were annotated. Annotation of compounds was performed automatically with an in-house spectral library including fatty acid methyl ester-based retention indices. Furthermore, manual matching against the National Institute of Standards and Technology 17 library, the FiehnLib library, and the Golm Metabolome Database (http://gmd.mpimp-golm.mpg.de/analysisinput.aspx) was performed to assess the identity of marker candidates. At this stage, metabolites were also checked with respect to their reproducibility and potential coelution and, if necessary, metabolites not reproducible or with unacceptable coelution were removed. In Supplementary Table S3, data for all relevant and annotated metabolites from untargeted GC×GC-MS are combined with data for the 44 metabolites detected by targeted approaches.

### 2.8 Metabolite classification

Metabolites were classified according to the Metabolomics Standards Initiative (MSI) (Sumner et al., 2007). For biological interpretation, relevant metabolites with MSI level 1 or 2 (identified or putatively annotated) were manually assigned to major and specific metabolic pathways/classes based on information provided by the human metabolome database (https://hmdb.ca/), Version 5.0 (Wishart et al., 2022) and the Kyoto Encyclopedia of Genes and Genomes PATHWAY database (https://www.genome.jp/kegg/pathway.html) (Aoki and Kanehisa, 2005). Since some identified metabolites have various origins or participate in different metabolic pathways, an unambiguous metabolite classification was not always possible. With regard to sugar-related compounds, we applied a clear procedure for metabolite classification: If sugars or sugar derivatives show an exogenous origin and are not known to be produced in human metabolism (e.g., isomaltose, lactose, gluconate), they were assigned to the major pathway/class “xenobiotic metabolites” and the sub-pathway “sugars, sugar substitutes and sugar derivatives.” In contrast, compounds that can be endogenously produced in human metabolism (e.g., lactate, pyruvate, fructose) were classified to the major pathway “carbohydrate metabolism” and the respective sub-pathway (Supplementary Tables S3, S5). When interpreting the results, it has been kept in mind that some metabolites can be assigned to more than one metabolic pathway. Analytes with MSI level 3 or 4 remained unclassified/unknown.

## 3 Results

### 3.1 Participant characteristics

Descriptive characteristics of participants and exercise parameters are summarized in Table 1. The study sample consisted of 16 healthy men with a mean age of 24.9 years, a mean percentage fat mass of 15.0%, and a mean VO_2peak_ of 56.6 mL × kg^−1^ × min^−1^. While the CME trial was performed at a mean workload of 129.4 W, the CVE trial was performed at a significantly higher mean workload of 224.8 W (*p* <0.0001). Likewise, a significantly different HR_max_ was reached during exercise interventions (*p* = 0.005). As depicted in Supplementary Table S2, participants consumed a carbohydrate-rich breakfast at both intervention days. Although slightly significant differences between the percentage of carbohydrate (*p* = 0.0214) and protein (*p* = 0.0054) intake were observed between trials, no significant difference was observed for the actual energy intake. In the post-exercise period of the CME and CVE trial, participants drank an average of 2.2 and 2.7 L water, respectively. The liquid intake was significantly different with a higher mean in the CVE trial (*p* = 0.0012). Concomitant with an increased water intake after both exercise interventions, osmolality of urine samples decreased from U00 to U03 (Supplementary Figure S1), showing significantly lower values at U03 after CVE compared to CME.

**TABLE 1:** Descriptive characteristics of participants (*n* = 16) and exercise parameters.

Basic characteristics	Mean ± SD	Min.	Max.
Age (years)	24.9 ± 2.8	18.7	30.3
Body height (cm)	182.9 ± 5.6	175.0	191.0
Body weight (kg)	75.5 ± 7.2	67.0	90.8
Body mass index (kg × (m^2^)^−1^)	22.6 ± 1.8	20.2	25.8
Lean body mass (kg)	64.1 ± 6.1	56.8	75.4
Fat mass (%)	15.0 ± 4.7	7.4	24.8
Blood pressure, systolic (mmHg)	124.4 ± 10.3	108.0	146.0
Blood pressure, diasystolic (mmHg)	76.2 ± 6.7	66.0	87.0
HR_rest_ (bpm)	63.4 ± 15.8	49.0	112.0

Preliminary exercise testing			
VO_2peak_ (mL × kg^−1^ × min^−1^)	56.6 ± 6.7	45.0	68.0
P_max_ (W)	337.7 ± 44.0	274.0	450.0
P_IAT_ (W)	213.7 ± 34.2	149.5	289.6
HR_max_ (bpm)	191.9 ± 8.2	173.0	204.0

Exercise interventions			
CME trial			
P_50%_ (W)	129.4* ± 21.7	77.0	156.0
HR_max_ (bpm)	131.6^#^ ± 15.1	105.0	153.0
CVE trial			
P_75%_ (W)	224.8* ± 33.1	163.0	270.0
HR_max_ (bpm)	159.9^#^ ± 23.3	100.0	187.0

CME, continuous moderate exercise; CVE, continuous vigorous exercise; HR_max_, maximum heart rate; HR_rest_, resting heart rate; Max, maximum; Min, minimum; P_50%_, power at 50% VO_2peak_; P_75%_, power at 75% VO_2peak_; P_IAT_, power at individual anaerobic threshold; P_max_, maximal power; SD, standard deviation; VO_2peak_, peak oxygen uptake. */^#^: significant difference (*p* <0.001) between CME and CVE trials based on paired Wilcoxon signed-rank test.

### 3.2 Metabolomics data

Regarding the 44 urinary metabolites identified by targeted approaches and the 526 spectral features detected by untargeted GC×GC-MS, no significant differences were observed for the performed pre-tests, which means 1) that the baseline levels (U00) of metabolites were comparable between both interventions, 2) that the order of interventions had no influence, and 3) that spot urine samples before and 24 h after intervention were comparable within the CME and CVE trial, respectively.

The statistical analyses depicted in Figure 2 led to 35 metabolites (targeted approach) and 200 spectral features (untargeted approach) with significant differences for at least one of the performed group comparisons. An additional filter for identifying *exercise-responsive* (*within-group* comparisons) or *intensity-dependent* (*between-group* comparisons) metabolites was applied, namely a median FC >1.5 or <0. $\bar{6}$ (for quantitative NMR and LC-MS data) and >2.0 or <0.5 (for relative quantitative GC×GC-MS data). By this way, a final number of 24 (targeted approach) and 69 (untargeted approach) relevant metabolites was obtained. Using the visual and heuristic approach for untargeted GC×GC-MS data, 11 additional metabolites were found to be interesting (Figure 2). From a total of 104 relevant metabolites, six (4-hydroxyphenylacetate, alanine, *cis*-aconitate, hippurate, hypoxanthine, and lactate) were identified by both targeted and untargeted approaches. For metabolic pathway classification, those doubly detected metabolites were only considered once. As illustrated in Supplementary Figure S2, most of the 57 relevant urinary metabolites with MSI level 1 or 2 belonged to AA metabolism or xenobiotic metabolites (each 35.1%), followed by carbohydrate metabolism and mammalian-microbial cometabolism (each 7.0%), energy metabolism and nucleotide metabolism (each 5.3%), cofactors and vitamins metabolism (3.5%), and lipid metabolism (1.8%). A metabolic pathway map of relevant metabolites with MSI level 1 or 2 is additionally provided in Supplementary Figure S3 (for CME) and Supplementary Figure S4 (for CVE).

**FIGURE 3:**
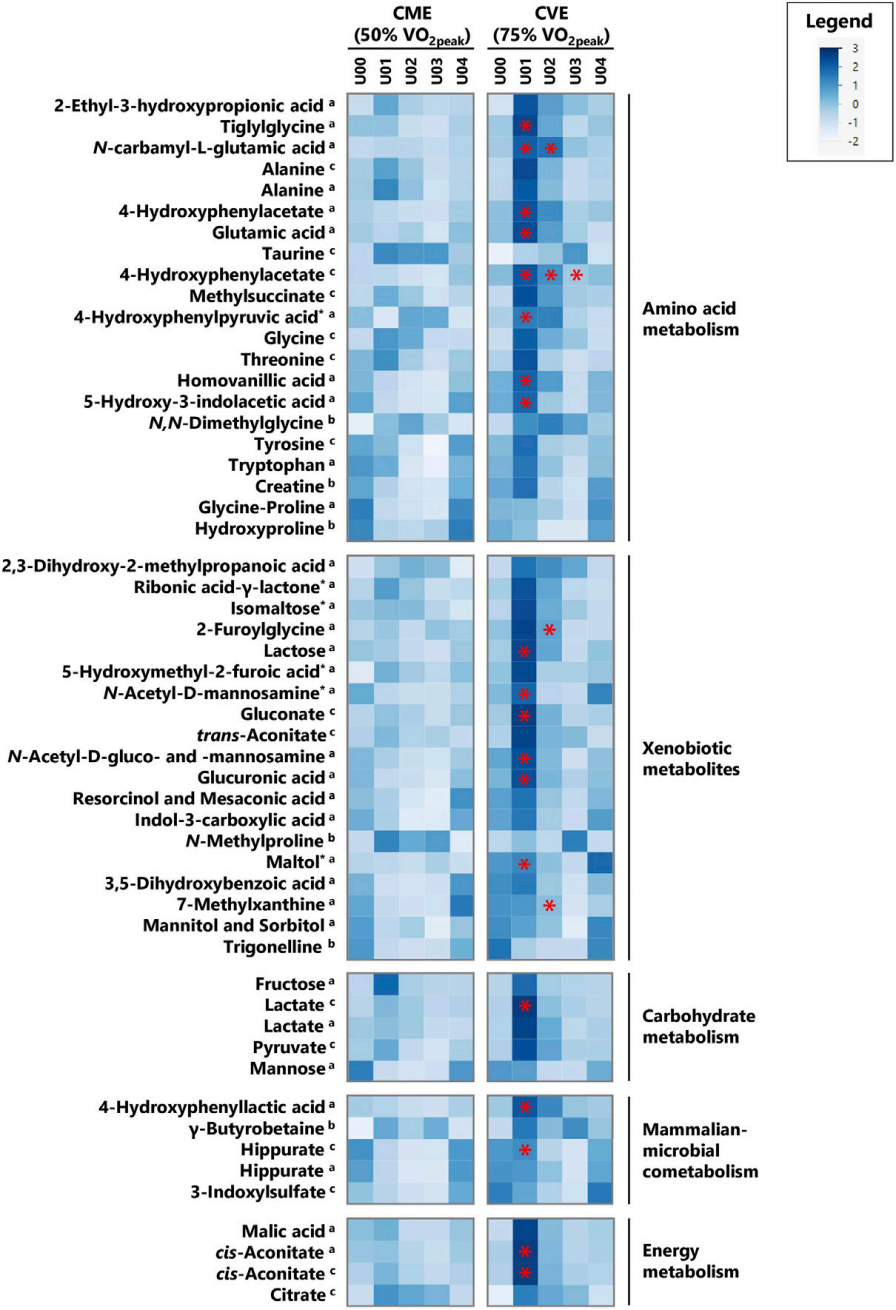
Heatmap of *z*-transformed median concentrations or signal intensities per urine sample time point in the CME and CVE trial for *exercise-responsive* and/or *intensity-dependent* metabolites, sorted by most relevant major metabolic pathways/classes and decreasing median FCs for U01 (CVE)/U00 (CVE) ratio. Metabolites with relevant differences between interventions are indicated with a red asterisk. ^a^: GC×GC-MS-detected; ^b^: LC-MS-detected; ^c^: NMR-detected; *: evaluated based on heuristic/visual approach; U: unknown analyte.

**FIGURE 4:**
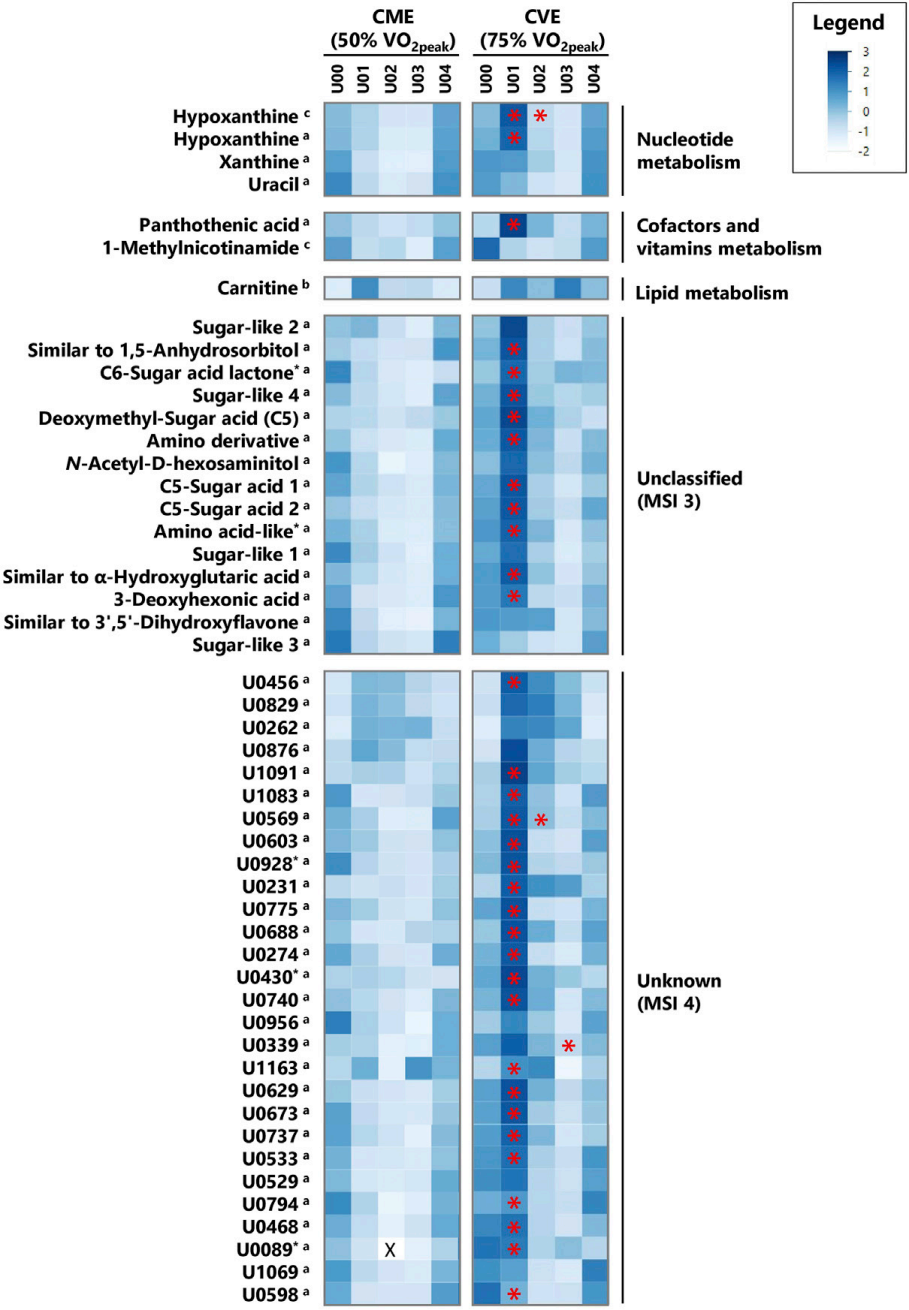
Heatmap of *z*-transformed median concentrations or signal intensities per urine sample time point in the CME and CVE trial for *exercise-responsive* and/or *intensity-dependent* metabolites, sorted by most relevant major metabolic pathways/classes and decreasing median FCs for U01 (CVE)/U00 (CVE) ratio (*continued*). Metabolites with relevant differences between interventions are indicated with a red asterisk. ^a^: GC×GC-MS-detected; ^b^: LC-MS-detected; ^c^: NMR-detected; *: evaluated based on heuristic/visual approach; U: unknown analyte; X: missing values.

**FIGURE 5:**
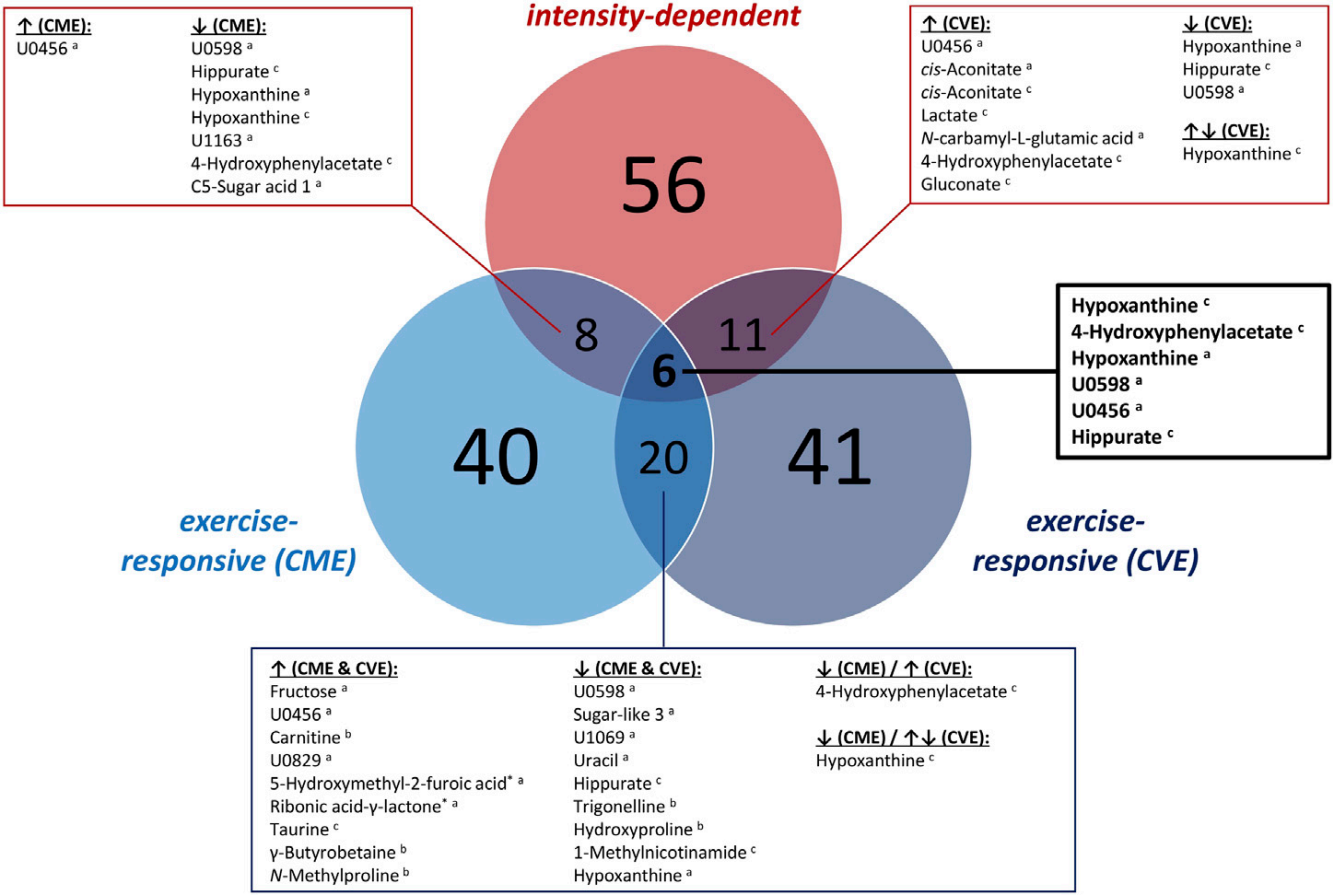
Venn diagram summarizing the number of *exercise-responsive* as well *as intensity-dependent* urinary metabolites. ^a^: GC×GC-MS-detected; ^b^: LC-MS-detected; ^c^: NMR-detected; *: relevant metabolites based on heuristic/visual approach; U: unknown analyte.

In Supplementary Table S5, all 104 relevant metabolites are listed according to metabolic pathways, including information on MSI levels, direction of change for both exercise trials, and median FCs in case of significant comparisons or relevant visual differences. Figures 3, 4 depict a heatmap of *z*-transformed median concentrations or signal intensities per sampling time point in the CME and CVE trial for all relevant, i.e., *exercise-responsive* or *intensity-dependent*, metabolites. Boxplots of those metabolites are additionally provided in Supplementary Data Sheet S2. The Venn diagram in Figure 5 summarizes the total number of *exercise-responsive* plus *intensity-dependent* metabolites, as well as respective overlaps. Analytical platforms of all relevant metabolites are indicated in Supplementary Table S5, Figures 3, 4. In the following sub-sections, for metabolites detected in more than one analytical platform, the respective platform is indicated by superscript letters (^a^: GC×GC-MS; ^b^: LC-MS; ^c^: NMR) along with FC presentation.

#### 3.2.1 Alterations in response to CME and/or CVE (*exercise-responsive* metabolites)

As illustrated in Figure 5, 40 and 41 *exercise-responsive* urinary metabolites were detected for the CME and CVE intervention, respectively. In general, 10 metabolites increased (↑) and 30 metabolites (↓) decreased in response to CME, whereas 30 metabolites increased, 10 metabolites decreased, and one metabolite initially increased and then decreased after CVE (Supplementary Table S5). For the CME trial, the highest number of relevant metabolites were observed for the comparison between U00 and U03 (3 ↑, 30 ↓), followed by the comparisons between U00 and U02 (2 ↑, 15 ↓) and U00 and U01 (8 ↑, 4 ↓) (Supplementary Table S5). Regarding immediate post-exercise alterations in metabolite concentrations, i.e., from U00 to U01, high FCs were documented for, e.g., fructose (FC = 22.0), carnitine (FC = 3.0), 5-hydroxymethyl-2-furoic acid (FC = 2.5), ribonic acid-γ-lactone (FC = 2.2), or taurine (FC = 1.8), while low FCs were shown for, e.g., hippurate (FC = 0.5^c^), trigonelline (FC = 0.6), or hydroxyproline (FC = 0.6). Of those metabolites with high FCs, only taurine remained elevated at both U02 and U03. In contrast, all metabolites with low FCs showed a persistent decrease from U00 to U03. Most post-exercise changes comprised late decreases, with 23 metabolites demonstrating FCs <0.5 at U02 and/or U03, e.g., xanthine, hypoxanthine, uracil, hippurate, glycine-proline, tyrosine, or tryptophan (Figures 3, 4). For the CVE trial, most relevant metabolites were documented for the comparison between U00 and U01 (30 ↑, 1 ↓), followed by the comparisons between U00 and U03 (6 ↑, 11 ↓) and U00 and U02 (7 ↑, 7 ↓) (Supplementary Table S5). With regard to immediate post-exercise increases in metabolite concentrations, i.e., from U00 to U01, high FCs were observed for, e.g., fructose (FC = 15.7), malic acid (FC = 4.2), 2-ethyl-3-hydroxypropionic acid (FC = 3.5), *cis*-aconitate (FC = 3.1^a^/2.8^c^), ribonic acid-γ-lactone (FC = 2.9), lactate (FC = 2.8^c^/2.0^a^), alanine (FC = 2.4^c^/2.3^a^), hypoxanthine (FC = 2.1^c^), pyruvate (FC = 2.0), taurine (FC = 2.0), or citrate (FC = 1.9). While most of these metabolites only showed a transient increase at U01, taurine, citrate, and two unknowns remained elevated at U02 and U03. Metabolites with decreasing urinary concentrations included trigonelline, which showed a persistent decline from U00 to U03 (FCs ≤0.6), and ten metabolites with FCs <0.5 at U02 and/or U03, e.g., hydroxyproline, uracil, hypoxanthine, and hippurate (Figures 3, 4).

In total, 20 of the *exercise-responsive* metabolites were identical between the CME and CVE trial, belonging to the major metabolic pathways/classes xenobiotic metabolites (5-hydroxymethyl-2-furoic acid, ribonic acid-γ-lactone, *N*-methylproline, trigonelline), AA metabolism (taurine, 4‐hydroxyphenylacetate, hydroxyproline), nucleotide metabolism (hypoxanthine, uracil), mammalian-microbial cometabolism (γ-butyrobetaine, hippurate), carbohydrate metabolism (fructose), lipid metabolism (carnitine), cofactors and vitamins metabolism (1-methylnicotinamide), or being unclassified/unknown. As depicted in Figure 5, most of these overlapping metabolites showed a similar response to both exercise interventions, except for 4-hydroxyphenylacetate and hypoxanthine.

#### 3.2.2 Differences between CME and CVE (*intensity-dependent* metabolites)

As summarized in Figure 5, 56 metabolites showed relevant post-exercise differences between the CME and CVE trial. In fact, the highest number of *intensity-dependent* metabolites were observed for the comparison between both exercise trials at U01 (53 higher in CVE), followed by the comparisons at U02 (6 higher in CVE) and U03 (1 higher, 1 lower in CVE) (Supplementary Table S5). Regarding U01, the highest FCs for CVE/CME ratio were observed for 4-hydroxyphenylacetate (FC = 3.6^a^/2.9^c^), *N*-carbamyl-L-glutamic acid (FC = 3.2), glutamic acid (FC = 3.2), pantothenic acid (FC = 3.1), maltol (FC = 3.1), and two unknowns. FCs >1.5 were found for, e.g., hypoxanthine, 4-hydroxyphenyllactic acid, 4-hydroxyphenylpyruvic acid, *N*-acetyl-D-mannosamine, *cis*-aconitate, tiglylglycine, homovanillic acid, lactate, hippurate, and a number of unknown/unclassified analytes. Of those metabolites with immediate post-exercise differences between exercise trials, *N*-carbamyl-L-glutamic acid, 4-hydroxyphenylacetate, hypoxanthine, and one unknown also showed higher post-exercise urinary concentrations at U02 after CVE compared to CME (FCs ≥1.5). Other relevant metabolites at U02 were 2-furoylglycine (FC = 2.7) and 7-methylxanthine (FC = 2.5). Only 4-hydroxyphenylacetate showed FCs ≥2.0^c^ at all post-exercise urine sampling time points.

Most of the *intensity-dependent* metabolites belonged to the major metabolic pathway/class xenobiotic metabolites (maltol, 2-furoylglycine, *N*-acetyl-D-mannosamine, 7-methylxanthine, *N*-acetyl-D-gluco- and mannosamine, lactose, glucuronic acid, gluconate), followed by AA metabolism (4-hydroxyphenylacetate, *N*-carbamyl-L-glutamic acid, glutamic acid, 4-hydroxyphenylpyruvic acid, tiglyglycine, 5-hydroxy-3-indolacetic acid, homovanillic acid), mammalian-microbial cometabolism (hippurate, 4-hydroxyphenyllactic acid), energy metabolism (*cis*-aconitate), nucleotide metabolism (hypoxanthine), carbohydrate metabolism (lactate), cofactors and vitamins metabolism (pantothenic acid), or were unclassified/unknown.

When additionally comparing the absolute urinary excretion of targeted NMR and LC-MS metabolites during each sample collection phase between trials, only hypoxanthine showed significant differences at U01 (FC = 2.6, for CVE/CME ratio). The cumulative excretion of hypoxanthine was also significantly different with a higher mean in the post-exercise period of CVE compared to CME (FC = 1.9). While 15 other metabolites, e.g., 4-hydroxyphenylacetate, lactate, hippurate, sarcosine, citrate, or taurine, also showed relevant median FCs >1.5 or <0. $\bar{6}$ for selected comparisons between trials, *p*-values were >0.05 after FDR correction (Supplementary Table S6, Supplementary Data Sheet S3).

#### 3.2.3 Overlap between *exercise-responsive* and *intensity-dependent* metabolites

As illustrated in Figure 5, certain overlaps between *exercise-responsive* and *intensity-dependent* metabolites were observed. Boxplots of those overlapping metabolites are provided in Supplementary Figures S5, S6. In total, six metabolites (hypoxanthine (detected by NMR and GC×GC-MS), 4-hydroxyphenylacetate, hippurate, and two unknowns) showed relevant differences in all cases, i.e., within and between the CME and CVE trial. While hypoxanthine increased at U01 only in response to the CVE trial (FC = 2.1^c^/1.7^a^), decreasing concentrations were observed at U02 and/or U03 within both the CME and CVE trial. Different responses to the exercise trials were also documented for 4-hydroxyphenylacetate, which showed a late decrease within the CME trial (FC = 0.6^c^ at U03) but an immediate increase within the CVE trial (FC = 1.9^c^ at U01), and hippurate, which showed a persistent decrease after CME (FCs ≤0.5^c^, U01 to U03) but a late decrease after CVE (FCs ≤0.6^c^, U02 to U03) (Supplementary Figure S5). Two further *intensity-dependent* metabolites (C5-sugar acid 1, one unknown) decreased at U03 in response to the CME trial (FCs ≤0.5), while being unchanged after CVE. Conversely, five *intensity-dependent* metabolites (*cis*-aconitate (detected by NMR and GC×GC-MS), lactate, *N*-carbamyl-L-glutamic acid, and gluconate) showed immediate increases at U01 in response to the CVE trial (FCs ≥1.7). However, they remained unchanged after CME (Supplementary Figure S6).

## 4 Discussion

This study provides a comprehensive overview and comparison of urinary metabolite changes after 30-min CME or CVE, pointing out potential metabolic markers reflective of exercise intensity. Our main finding is that the CVE trial induced a greater response in the urinary metabolome than the CME trial, as demonstrated by a higher number of metabolites with immediate post-exercise alterations after CVE (see Section 3.2.1) and relevant metabolite differences between both interventions, particularly at U01 (see Section 3.2.2). As expected, most alterations in the urinary metabolome occurred in the first post-exercise collection phase (CVE>CME), returning to baseline within 24 h after U00, which has so far been documented by one recent urine exercise metabolomics study (Siopi et al., 2017). In total, the observed metabolite changes indicate an increased degradation of carbohydrates, purine nucleotides, or AAs especially after CVE, confirming well-known exercise-induced perturbations in pathways related to energy metabolism. However, some novel exercise-related urinary metabolites of foreign origin (e.g., xenobiotics or microbial metabolites) or being associated with vitamins metabolism were also revealed. In the next sub-section, a discussion of the most important *exercise-responsive* and/or *intensity-dependent* metabolites is provided, separated by metabolic pathways/classes.

### 4.1 AA metabolism

Regarding proteinogenic AAs, alanine, glycine, and threonine showed increasing concentrations at U01 after CVE, whereas both tyrosine and tryptophan demonstrated late decreases at U03 after CME. Glutamic acid was higher at U01 after CVE compared to CME (Supplementary Table S5). While the rise in urinary alanine was consistent across several endurance exercise-related metabolomics studies (Mukherjee et al., 2014; Siopi et al., 2017; Kistner et al., 2020), previous work either confirmed higher urinary glycine and threonine after CME (Siopi et al., 2017) or incremental exercise (Kistner et al., 2020), or reported lower urinary glycine (Mukherjee et al., 2014) or threonine (Muhsen Ali et al., 2016) in response to 45-min endurance exercise at varying workloads. A decrease in tryptophan has also been previously detected in post-exercise 24-h urine samples (Mukherjee et al., 2014). Contrary to our results, urinary tyrosine increased 2 h after CME in a previous study (Siopi et al., 2017). Observed changes in urinary AAs might be related to altered circulating concentrations due to an increased mobilization of glucogenic AAs (e.g., alanine, glutamic acid, glycine, threonine), especially during CVE, or an increased use of also ketogenic AAs (e.g., tryptophan, tyrosine) in the late post-exercise period, i.e., when participants remained in a fasting state. However, we cannot explain why decreases in ketogenic AAs exclusively appeared after CME. In fact, only two non-proteinogenic AAs (taurine, hydroxyproline) and 4-hydroxyphenylacetate were responsive to both CME and CVE (Figure 5). Post-exercise increases in urinary taurine have been reported previously (Cuisinier et al., 2001; Siopi et al., 2017; Kistner et al., 2020), whereas decreases in hydroxyproline have, to our knowledge, been observed in blood (Nayor et al., 2020), but not yet in urine. While elevated urinary hydroxyproline is assumed to indicate muscle collagen breakdown (Brown et al., 1997), lower levels might be related to a higher conversion of hydroxyproline into glycine (Wu, 2020), which tended to increase after both CME and CVE. Taurine is highly abundant in skeletal muscles. It is involved in cell volume regulation, membrane stabilization, antioxidant activities (Lambert et al., 2015), and, potentially, energy metabolism (Wen et al., 2019). The observed persistent rise in urinary taurine might be traced back to an increased muscular taurine release, which has already been discussed as a mechanism to control either osmoregulation or calcium homeostasis in response to endurance exercise (Ward et al., 1999; Cuisinier et al., 2002). 4-hydroxyphenylacetate, a product of tyrosine catabolism, tended to decrease after CME until U03, whereas it immediately increased after CVE, showing significantly different urinary concentrations between interventions at all post-exercise timepoints (Supplementary Table S5). Other tyrosine degradation products (e.g., 4-hydroxyphenylpyruvic acid, homovanillic acid) as well as a serotonin breakdown metabolite (5-hydroxy-3-indolacetic acid) and a derivative of glutamic acid (*N*-carbamyl-L-glutamic acid) were also higher after CVE compared to CME. Similarly, intermediates of leucine (methylsuccinate) or isoleucine (tiglylglycine, 2-ethyl-3-hydroxypropionic acid) catabolism showed either post-exercise increases in response to CVE or significant differences between trials at U01 (Supplementary Table S5), possibly reflecting an increased muscular utilization of branched-chain AAs during CVE (Tarnopolsky, 2004; Holeček, 2018). To our knowledge, metabolomics studies have reported neither exercise-induced nor intensity-dependent changes in the urinary concentration of those metabolites [except for methylsuccinate (Kistner et al., 2020)] so far.

### 4.2 Xenobiotic metabolites

Of xenobiotic metabolites, four were responsive to both CME and CVE (Figure 5) and eight differed between interventions (Figure 3). Most relevant xenobiotics were classified as sugar derivatives and food or plant constituents (Supplementary Table S5). While the sugar acid derivative ribonic acid-γ-lactone and 5-hydroxymethyl-2-furoic acid, an organic acid naturally occurring in urine (Bouatra et al., 2013), showed post-exercise increases at U01 in both trials, two diet-related metabolites demonstrated either a late increase at U03 [*N*-methylproline, present in citrus fruits (Servillo et al., 2011)] or a transient decrease from U00 to U03 [trigonelline, present in coffee (Madrid-Gambin et al., 2016)]. Regarding intensity-dependent xenobiotics, higher urinary levels after CVE compared to CME were documented for sugars and sugar derivatives, such as maltol, *N*-acetyl-D-mannosamine, *N*-acetyl-D-gluco- and mannosamine, lactose, and glucuronic acid, at U01, and coffee or cocoa biomarkers, such as 2-furoylglycine (Madrid-Gambin et al., 2016) and 7-methylxanthine (Martínez-López et al., 2014), at U02. Gluconate, which is present in fruits, dairy products, or honey (Ramachandran et al., 2006), was the only intensity-dependent xenobiotic that increased at U01 after CVE (Supplementary Table S5). In brief, many exercise-related changes in xenobiotic metabolites have not been reported previously and might be either related to exercise-elicited changes in their renal excretion (e.g., increased glomerular filtration or decreased tubular reabsorption) or possibly reflect the intake of foods not contained in the dietary plan (e.g., coffee) on the day before interventions. Unfortunately, discrepancies between estimated and actual food consumption have not been registered. Finally, those metabolites classified as xenobiotics in this study seem to be rather negligible when selecting urinary markers specific for acute physical exercise.

### 4.3 Carbohydrate metabolism

Regarding carbohydrate metabolism, only fructose was responsive to both CME and CVE, showing the strongest rise from U00 to U01 of all assessed metabolites (Supplementary Table S5). To our knowledge, metabolomics studies have observed an increasing post-exercise abundance of fructose in saliva (Zauber et al., 2012), but not yet in urine. Like glucose, fructose can be utilized for carbohydrate oxidation during exercise (Jentjens et al., 2004). However, urinary levels of this monosaccharide did not show any intensity-dependent differences in our study. Since urinary fructose is recognized as a biomarker of sugar intake (Clarke et al., 2019), we suppose its post-exercise increase to be a result of the dietary intake of honey or jam during breakfast. Contrary to fructose, pyruvate and lactate showed immediate post-exercise increases only in response to CVE. The latter also exhibited significant differences between trials at U01 (Supplementary Table S5). Elevated levels of urinary pyruvate and lactate after endurance exercise have previously been observed (Mukherjee et al., 2014; Muhsen Ali et al., 2016; Kistner et al., 2020). Although higher post-exercise increases in urinary pyruvate and lactate have already been documented after interval or resistance exercise than after CME (Siopi et al., 2017), we firstly detected those differences between two endurance trials at different intensities. Blood glucose and muscle glycogen are the major carbohydrate substrates to generate adenosine triphosphate (ATP) during exercise, and its rate of degradation is known to be dependent on exercise intensity (Hargreaves and Spriet, 2020). Pronounced increases in urinary pyruvate and lactate after CVE can therefore be seen as a result of augmented rates of glycolysis at higher exercise intensities. As end-products of glucose breakdown, pyruvate and lactate can be released into circulation and partly excreted *via* urine (Adeva-Andany et al., 2014). Interestingly, lactate has been considered as a metabolic waste product for many years, but is now recognized as a substrate for oxidative metabolism and as a signaling molecule that mediates exercise adaptations, such as mitochondrial biogenesis (Brooks, 2020; Hargreaves and Spriet, 2020). To what extent urinary concentrations might reflect those (non-)metabolic functions of lactate, needs to be examined in further studies.

### 4.4 Mammalian-microbial cometabolism

Of microbial metabolites, γ-butyrobetaine and hippurate were responsive to CME and CVE (Figure 5). While the former showed increases at U01 and U03 after both trials, the latter tended to persistently decline from U00 to U03 and additionally revealed significant differences between CME and CVE at U01 (Supplementary Table S5). γ-butyrobetaine is known as a precursor for carnitine biosynthesis (Vaz and Wanders, 2002), but has recently emerged as an intermediate of carnitine degradation by the gut microbiota (Koeth et al., 2014). To our knowledge, exercise-related alterations in urinary γ-butyrobetaine have not been observed so far. As urinary carnitine showed a similar response to both exercise trials (see Section 4.8), it cannot be excluded that the intake of carnitine-containing food [e.g., cheese (Vaz and Wanders, 2002)] at breakfast also led to the observed increase in urinary γ-butyrobetaine. Hippurate excretion has been associated with the microbial degradation of dietary components such as vegetables, fruits, tea, or coffee (Lees et al., 2013). Former studies also showed decreasing post-exercise hippurate in urine (Mukherjee et al., 2014; Pechlivanis et al., 2015; Kistner et al., 2020). Since active renal tubular secretion is the principle elimination route for hippurate (Lees et al., 2013), its lower post-exercise urinary excretion might be traced back to a transiently impaired renal function due to renal tubular acidosis resulting from high lactate concentrations (Pechlivanis et al., 2015). However, it remains speculative why the decline in urinary hippurate at U01 was more pronounced in the CME trial. For 4-hydroxyphenyllactic acid, a microbial metabolite resulting from tyrosine degradation (Mu et al., 2010), higher urinary concentrations after CVE compared to CME were detected (Supplementary Table S5). 3-indoxylsulfate, a mammalian-microbial cometabolite resulting from tryptophan degradation (Zhang and Davies, 2016), showed late declines at U02 and U03 only after CVE (Supplementary Table S5). While urinary 3-indoxylsulfate also tended to decrease after incremental exercise in our earlier study (Kistner et al., 2020), higher urinary levels were reported after endurance exercise in three males (Daskalaki et al., 2015). Even if 3-indoxylsulfate is a known uremic toxin, being related to vascular disease and renal dysfunction (Zhang and Davies, 2016), underlying mechanisms as well as the physiological relevance of lower post-exercise urinary concentrations are largely speculative. In summary, CME and/or CVE led to rather unexpected changes in the level of microbial metabolites in urine. Future studies are needed to investigate in detail how the intestinal production and/or absorption of microbial metabolites, their hepatic metabolism, and renal excretion are affected by endurance exercise, and which physiological implications this has.

### 4.5 Energy metabolism

All analyzed intermediates of the TCA cycle (i.e., *cis*-aconitate, citrate, malate) showed increasing urinary concentrations at U01 only after CVE. In detail, citrate was elevated during the whole post-exercise period and *cis*-aconitate exhibited intensity-dependent differences at U01. Also, malic acid tended to be higher at U01 after CVE compared to CME (Supplementary Table S5). In our earlier study, urinary *cis*-aconitate and citrate also tended to increase after incremental exercise (Kistner et al., 2020), whereas urinary citrate slightly decreased in previous metabolomics studies focusing on CME (Siopi et al., 2017) or continuous exercise at varying workloads (Mukherjee et al., 2014). To the best of our knowledge, increasing *cis*-aconitate (Peake et al., 2014), citrate (Peake et al., 2014; Danaher et al., 2015; Zafeiridis et al., 2016), and malate (Lewis et al., 2010; Chorell et al., 2012; Danaher et al., 2015; San-Millán et al., 2020; Shi et al., 2020) have so far mostly been documented in blood, e.g., in response to high-intensity interval exercise (Peake et al., 2014; Danaher et al., 2015; Zafeiridis et al., 2016) and endurance exercise at increasing intensities (Chorell et al., 2012; San-Millán et al., 2020) or of longer duration (Lewis et al., 2010; Shi et al., 2020). In this study, we pointed out specific TCA cycle intermediates in urine as potential markers of acute endurance exercise at high workloads. It seems likely that these compounds were released from muscles into blood due to an increased TCA cycle flux during exercise (Bowtell et al., 2007), being finally excreted *via* urine. While intensity-dependent changes likely reflect higher muscular energy needs during CVE than CME, the functional role of TCA cycle metabolites in blood/urine still remains to be elucidated (Bowtell et al., 2007; Peake et al., 2014). Of note, recent rodent studies assume that TCA cycle intermediates also have non-metabolic signaling roles, possibly acting as humoral mediators of exercise adaptation processes, e.g., by promoting post-translational protein modifications (Martínez-Reyes and Chandel, 2020; Maurer et al., 2021).

### 4.6 Nucleotide metabolism

With regard to nucleotide metabolism, hypoxanthine and uracil were responsive to both CME and CVE, showing late decreases at U02 and/or U03. Hypoxanthine also demonstrated relevant increases at U01 after CVE. Both its urinary concentration (Supplementary Table S5) and absolute excretion (Supplementary Table S6, Supplementary Data Sheet S3) at U01 were higher after CVE compared to CME, confirming earlier studies that observed post-exercise increases in urinary hypoxanthine primarily in response to high-intensive (interval) exercise and, to a lesser extent, CME (Gerber et al., 2014; Mukherjee et al., 2014; Muhsen Ali et al., 2016; Siopi et al., 2017). As the final ATP degradation product, hypoxanthine is released from muscles to blood and excreted *via* urine (Zielinski and Kusy, 2015). Hence, intensity-dependent differences in urinary hypoxanthine could be explained by a higher muscular purine nucleotide breakdown and/or saturation of purine salvage during CVE compared to CME. While hypoxanthine in blood has already been proposed as an indicator of exercise-related energetic stress (Zielinski and Kusy, 2015), our results underline existing evidence of urinary hypoxanthine as a marker of exercise intensity. Lower urinary hypoxanthine in the recovery period compared to U00 might potentially be traced back to restorative processes required for intramuscular ATP resynthesis. It is known that the purine nucleotide salvage pathway is capable to reutilize about 75% of intramuscular hypoxanthine in resting conditions (Edwards et al., 1979). However, its role in post-exercise purine recovery is currently unclear (Zielinski and Kusy, 2015).

### 4.7 Cofactors and vitamins metabolism

While pantothenic acid (vitamin B5) was significantly different with a higher mean at U01 after CVE compared to CME, 1-methylnicotinamide exhibited a late decrease at U02 and/or U03 after both trials (Supplementary Table S5). So far, metabolomics studies have shown increases in blood pantothenic acid in response to intensive interval (Siopi et al., 2019) or endurance (Lewis et al., 2010; Nayor et al., 2020) exercise. After CME, either decreasing blood (Siopi et al., 2019) or increasing urinary (Daskalaki et al., 2015; Muhsen Ali et al., 2016) levels have been observed. Given its role as a precursor of coenzyme-A (CoA), pantothenic acid is involved in, e.g., fatty acid metabolism and TCA cycle activity (Miller and Rucker, 2020). Since amounts above its renal threshold are excreted (Miller and Rucker, 2020), higher pantothenic acid in urine after CVE could hint at a higher mobilization and transport of this vitamin to skeletal muscles for CoA biosynthesis. Otherwise, as previously suggested (Daskalaki et al., 2015; Muhsen Ali et al., 2016), increased urinary levels might also indicate a reduced demand for CoA, reflecting an increased reliance on glycogen than lipids, which require an excess of free CoA (Dansie et al., 2014), for energy provision during CVE (Hargreaves and Spriet, 2020). 1-methylnicotinamide is an endogenous metabolite of nicotinamide and higher urinary outputs have been linked to increased utilization of nicotinamide adenine dinucleotide, a coenzyme central to energy metabolism (Okamoto et al., 2003). Since 1-methylnicotinamide tended to decrease after both exercise trials, our findings were rather contradictory to an increased nicotinamide metabolism, which could have been assumed especially in response to the CVE trial. However, 1-methylnicotinamide has recently been suggested as a myokine that might stimulate the mobilization of energy substrates (Ström et al., 2018) or exert vasoprotective effects (Chłopicki et al., 2012). Hence, decreasing urinary levels could potentially be related to a higher use of circulating 1-methylnicotinamide in response to exercise.

### 4.8 Lipid metabolism

Urinary carnitine increased at U01 after both CME and CVE (Supplementary Table S5). Apart from our earlier study (Kistner et al., 2020), a post-exercise rise of free carnitine in urine has not been noticed so far. Even if carnitine is involved in lipid metabolism, transporting long-chain fatty acids into the mitochondrial matrix (Stephens, 2018), its higher urinary concentrations might rather be explained by the intake of carnitine-containing food, e.g., dairy products (Vaz and Wanders, 2002), at breakfast.

### 4.9 Exercise, renal function, and metabolite excretion

As described in the literature, exercise can lead to alterations in renal function, including changes in renal hemodynamics (e.g., reduced renal plasma flow/glomerular filtration rate) or an increased protein excretion due to a higher glomerular permeability and/or partial inhibition of tubular reabsorption (Poortmans, 1984; Poortmans and Vanderstraeten, 1994). Though, a high degree of hydration seems to have an important effect on the glomerular filtration rate during exercise, reducing plasma protein clearance (Poortmans, 1984). With regard to the metabolites analyzed in this study, the question could be raised whether alterations in their urinary concentrations were related to processes in non-renal locations, such as skeletal muscle energy metabolism, or if they rather occurred due to exercise-elicited changes in renal excretion rates. As for lactate, it is known that exercise leads to increased urinary excretion due to both augmented production within active muscles, causing a rise in plasma lactate, and exercise-related saturation of tubular reabsorption (Poortmans and Vanderstraeten, 1994). However, it is not yet known how far tubular processes and renal excretion rates of those metabolites, which have been identified as either *exercise-responsive* or *intensity-dependent*, were specifically affected by moderate or vigorous endurance exercise. Indeed, participants were sufficiently hydrated during both exercise interventions and a similar time course of urinary osmolality was observed for the CME and CVE trial (Supplementary Figure S1). Moreover, no significant difference in absolute creatinine excretion was detected between trials (Supplementary Figure S7). These observations reveal no relevant changes regarding the general kidney function after the CVE trial compared to the CME trial, which could have explained the higher urinary metabolite concentrations at U01. Additionally, only 104 out of more than 600 detected metabolites and spectral features, respectively, showed relevant alterations after exercise or differences between trials (Figures 3, 4, Supplementary Table S5). In case of exercise-induced changes in renal function, such as a general increase in glomerular permeability or a general inhibition of tubular reabsorption, one could expect a majority of analytes showing higher urinary excretion, especially after CVE. However, this was not the case in this study.

### 4.10 Strengths and limitations

This study has several strengths. Firstly, it was characterized by highly standardized examinations (e.g., VO_2peak_ assessment) and strictly controlled experimental procedures (e.g., clearly defined urine sampling phases, controlled diet and hydration, or individualized exercise protocols). A crossover study design was chosen to adequately consider the inter-individual variability of the metabolome. The applied multi-platform metabolomics approach allowed to detect a large number of metabolites from different chemical classes and metabolic pathways. While targeted NMR and LC-MS analyses permitted the absolute quantification of metabolites with known identities, and, additionally, to calculate absolute and cumulative urinary metabolite excretion due to complete urine collection in each of the three post-exercise periods, untargeted GC×GC-MS offered the ability to detect previously unknown but important exercise-related metabolites. Of note, six relevant urinary metabolites were identified by both targeted and untargeted approaches, which can be seen as an internal validation of results, since correlations between methods were good. Thus, also similar post-exercise trends were observed for those doubly detected metabolites. Slight discrepancies in median FCs and significance levels could mainly be explained by different analytical precisions of the applied methods and selected boundaries for choosing relevant metabolites. Regrettably, some relevant GC×GC-MS analytes could not be identified with certainty and thus remained unknown. In total, our univariate statistical analyses should be regarded as an exploratory approach for obtaining first information about *exercise-responsive* and/or *intensity-dependent* metabolites. Unfortunately, the use of repeated-measures approaches such as multivariate analysis of variance has not been possible because we had to exclude 22 study samples from the analysis of untargeted metabolomics data (due to technical problems during the first two measurement days). Since this would have led to the exclusion of 14 out of 16 participants for multivariate analysis of variance, we rather conducted selected main comparisons based on non-parametric univariate tests. Another limitation is that no conclusions on lipid metabolism can be drawn based on the urinary metabolome. Consequently, future studies should also focus on the plasma metabolome, which comprises more lipid-related metabolites. Additionally, comparative metabolomics analyses in blood would facilitate the interpretation of observed metabolite alterations. Participants were sufficiently hydrated during exercise interventions and a similar time course of urinary osmolality was documented for the CME and CVE trial (Supplementary Figure S1). Furthermore, a comparable absolute urinary creatinine excretion was observed within and between trials (Supplementary Figure S7), respectively, suggesting that no relevant changes in the general kidney function have occurred. However, exercise-induced and/or intensity-dependent alterations of renal excretion (e.g., due to changes in glomerular permeability or tubular reabsorption) of specific metabolites cannot be entirely excluded and might potentially underlie the observed changes after CVE, particularly with regard to xenobiotic metabolites which are not known to be related to exercise-elicited changes in, e.g., skeletal muscle metabolism. Moreover, since the urinary excretion of some metabolites [e.g., taurine (Lambert et al., 2015), carnitine (Vaz and Wanders, 2002), or fructose (Clarke et al., 2019)] depends on their dietary intake, acute effects of the pre-exercise breakfast could not be excluded and should be kept in mind, especially if interpreting metabolites responsive to both exercise interventions. Due to the fact that exercise intensities for CME and CVE trials were calculated based on respiratory parameters (i.e., power at 50% or 75% of VO_2peak_), not directly considering metabolic parameters such as blood lactate thresholds, participants either exercised above (*n* = 10), at (*n* = 3), or below (*n* = 3) their P_IAT_ during CVE. Consequently, inter-individual variation in post-exercise metabolite alterations might partly be explained by differences in training status or sport disciplines of participants. Lastly, our results cannot be directly transferred to the general population. As this study included a homogenous group of young, active men, more research in both women and men of different ages and fitness status is required.

## 5 Conclusion

To our knowledge, this is the first study using a multi-platform metabolomics approach to examine acute changes in the urinary metabolome after two endurance exercise trials at different intensities. By providing temporal information on post-exercise metabolite alterations in urine, we showed that CVE caused more profound metabolic perturbations than CME, especially at U01 (i.e., until 45 min post-exercise). Differences in urinary metabolites are likely to reflect higher energy requirements during CVE, as demonstrated by greater shifts in metabolites related to cellular energy metabolism including glycolysis (e.g., lactate, pyruvate) and TCA cycle (e.g., *cis*-aconitate, malate), purine nucleotide breakdown (e.g., hypoxanthine), or whole-body substrate metabolism such as AA mobilization (e.g., alanine) and AA degradation (e.g., 4-hydroxyphenylacetate). Interestingly, some of the metabolites we identified as potential urinary markers of endurance exercise intensity (e.g., *cis*-aconitate, malate, lactate) have recently been discussed as signaling molecules with myokine-like functions. Future studies are needed to validate our findings, to elucidate underlying mechanisms as well as tissue origin, and to clarify whether acute metabolite alterations in urine reflect changes in circulating levels of those metabolites potentially involved in mediating beneficial adaptive responses to physical exercise. With particular regard to xenobiotic and microbial metabolites, it will be necessary to evaluate physiological mechanisms (e.g., altered intestinal uptake, hepatic metabolism, or renal excretion) contributing to the exercise-related changes in their urinary concentration.

## Data Availability

The original contributions presented in the study are included in the article/Supplementary Material, further inquiries can be directed to the corresponding authors.

## Supplementary Material

SUPPLEMENTARY TABLE S1 Overview of inclusion and exclusion criteria for study participation.

SUPPLEMENTARY TABLE S2 Overview of calculated energy and macronutrient intake for (pre-)intervention days, actual energy and macronutrient intake at breakfast, and post-exercise water intake on both intervention days.

SUPPLEMENTARY TABLE S3 Combined data set for all 44 metabolites detected by targeted approaches and 80 metabolites detected by untargeted GC×GC-MS and selected as relevant after statistical and heuristic approaches. Osmolality-normalized urinary metabolite concentrations in mmol/L per Osm/kg (targeted approaches) or signal intensities (untargeted GC×GC-MS) are shown. All relevant metabolites have been annotated and respective MSI levels, major and specific metabolic pathways/classes are indicated.

SUPPLEMENTARY TABLE S4 Data on absolute and cumulative urinary excretion (in µmol) for all 44 metabolites detected by targeted approaches.

SUPPLEMENTARY TABLE S5 Overview of 104 metabolites with relevant urinary concentration differences within or between trials, using statistical or visual/heuristic analyses and median FCs >2.0 or <0.5 (GC×GC-MS data) or >1.5 or <0. $\bar{6}$ (LC-MS and NMR data).

SUPPLEMENTARY TABLE S6
*Between-group* comparison of the absolute and cumulative urinary excretion of 16 metabolites with median FCs >1.5 or <0. $\bar{6}$ regarding CVE/CME ratio for at least one of the three post-exercise sampling time points or the whole post-exercise period.

SUPPLEMENTARY DATA SHEET S1 Details of GC×GC-MS analysis and data processing.

SUPPLEMENTARY DATA SHEET S2 Boxplots of 104 metabolites with relevant urinary concentration differences within or between trials, using statistical or visual/heuristic analyses and median FCs >2.0 or <0.5 (GC×GC-MS data) or >1.5 or <0. $\bar{6}\;($ LC-MS and NMR data). Metabolites are sorted in alphabetical order.

SUPPLEMENTARY DATA SHEET S3 Bar charts showing absolute urinary excretion (in µmol/min; medians and interquartile ranges are displayed) of 16 metabolites with median FCs >1.5 or <0. $\bar{6}$ for CVE/CME ratio at the three post-exercise sampling time points. Metabolites are sorted by decreasing median FCs for U01 (CVE)/U01 (CME) ratio. *: FDR-corrected *p*-value <0.05 for *between-group* comparisons.

SUPPLEMENTARY FIGURE S1 Boxplots showing osmolality of urine samples within the CME and CVE trial. *: *p*-value <0.05 for *between-group* comparisons.

SUPPLEMENTARY FIGURE S2 Classification of 57 *exercise-responsive* and/or *intensity-dependent* metabolites with MSI level 1 or 2 to major metabolic pathways/classes.

SUPPLEMENTARY FIGURE S3 Metabolic pathway map of relevant urinary metabolites with MSI level 1 or 2 (CME trial). Post-exercise changes are color coded as follows: dark red, increase at U01; red, increase at U02; light red, increase at U03, dark blue, decrease at U01; blue, decrease at U02; light blue, decrease at U03. ^1^: significant difference between groups at U01; ^2^: significant difference between groups at U02; ^3^: significant difference between groups at U03; grey: undetected metabolites; *: evaluated based on heuristic/visual approach; ?: existence of metabolic pathway in humans unclear.

SUPPLEMENTARY FIGURE S4 Metabolic pathway map of relevant urinary metabolites with MSI level 1 or 2 (CVE trial). Post-exercise changes are color coded as follows: dark red, increase at U01; middle red, increase at U02; light red, increase at U03, dark blue, decrease at U01; middle blue, decrease at U02; light blue, decrease at U03. ^1^: significant difference between groups at U01; ^2^: significant difference between groups at U02; ^3^: significant difference between groups at U03; grey: undetected metabolites; *: evaluated based on heuristic/visual approach; ?: existence of metabolic pathway in humans unclear.

SUPPLEMENTARY FIGURE S5 Boxplots of six metabolites with relevant urinary concentration differences between and within the CME and CVE trial, sorted by decreasing median FCs for U01(CVE)/U01(CME) ratio. On the y-axis, absolute concentrations in mmol/L per Osm/kg (targeted approaches) or signal intensities (untargeted GC×GC-MS) are displayed. *: relevant median FC >2.0 or <0.5 (GC×GC-MS data) or >1.5 or <0. $\bar{6}$ (NMR data) and FDR-corrected *p*-value <0.05 for *between-group* comparisons; ^#^: relevant median FC >2.0 or <0.5 (GC×GC-MS data) or >1.5 or <0. $\bar{6}$ (NMR data) and FDR-corrected *p*-value <0.05 for *within-group* comparisons to U00.

SUPPLEMENTARY FIGURE S6 Boxplots of seven metabolites with relevant urinary concentration differences between trials and within either the CME or CVE trial, sorted by decreasing median FCs for U01/U00 ratio. On the y-axis, absolute concentrations in mmol/L per Osm/kg (targeted approaches) or signal intensities (untargeted GC×GC-MS) are displayed. *: relevant median FC >2.0 or <0.5 (GC×GC-MS data) or >1.5 or <0. $\bar{6}$ (NMR data) and FDR-corrected *p*-value <0.05 for *between-group* comparisons; ^#^: relevant median FC >2.0 or <0.5 (GC×GC-MS data) or >1.5 or <0. $\bar{6}$ (NMR data) and FDR-corrected *p*-value <0.05 for *within-group* comparisons to U00.

SUPPLEMENTARY FIGURE S7 Bar charts showing absolute urinary creatinine excretion (medians and interquartile ranges are displayed) at the three post-exercise sampling time points within the CME and CVE trial.

## Abbreviations

AA, amino acid; ATP, adenosine triphosphate; CME, continuous moderate exercise; CoA, coenzyme-A; CVE, continuous vigorous exercise; FC, fold change; FDR, false discovery rate; GC×GC, two-dimensional gas chromatography; HR, heart rate; HR_max_, maximum heart rate; HR_rest_, resting heart rate; LC, liquid chromatography; MS, mass spectrometry; MSI, Metabolomics Standards Initiative; NMR, nuclear magnetic resonance; P_IAT_, power at individual anaerobic threshold; QC, quality control; TCA, tricarboxylic acid; V1, visit 1; V2, visit 2; VO_2peak_, peak oxygen uptake.
