# Supplementary material for: Acute effects of moderate vs. vigorous endurance exercise on urinary metabolites in healthy, young, physically active men—A multi-platform metabolomics approach
Source: Front Physiol. 2023 Jan 30;14:1028643. doi: 10.3389/fphys.2023.1028643 (PMC9927024; doi:10.3389/fphys.2023.1028643)

Variability Gauge

Variability Chart for 1-Methylnicotinamide

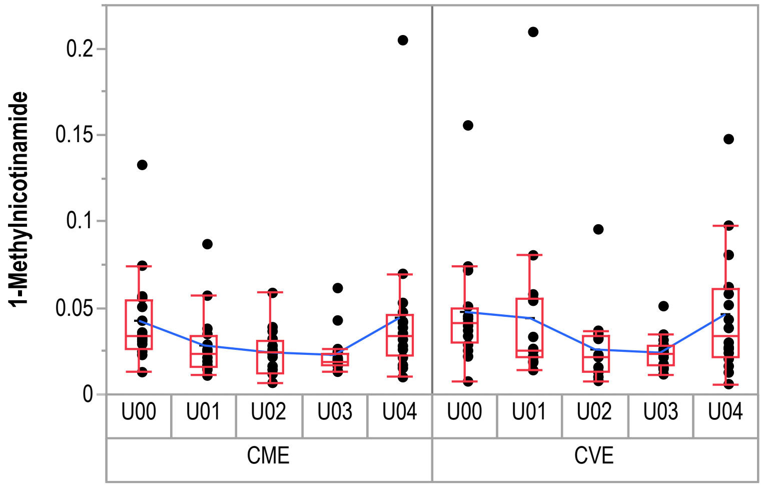

Variability Gauge

Variability Chart for 2,3-Dihydroxy-2-methylpropanoic acid

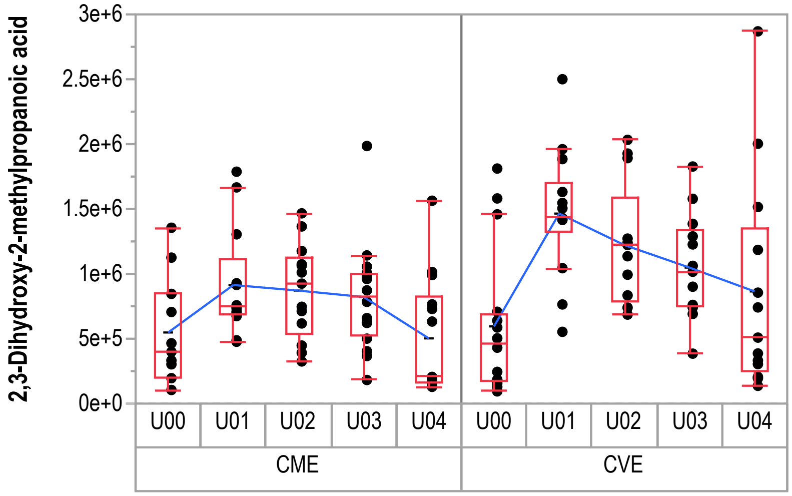

Variability Gauge

Variability Chart for 2-Ethyl-3-hydroxypropionic acid

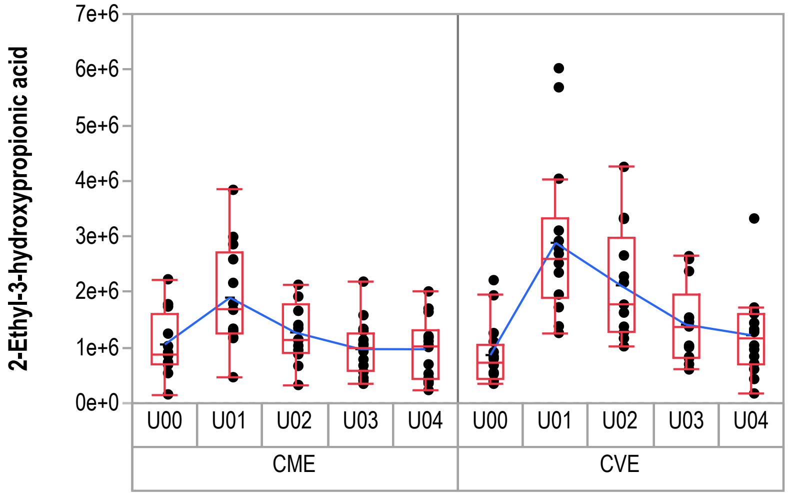

Variability Gauge

Variability Chart for 2-Furoylglycine

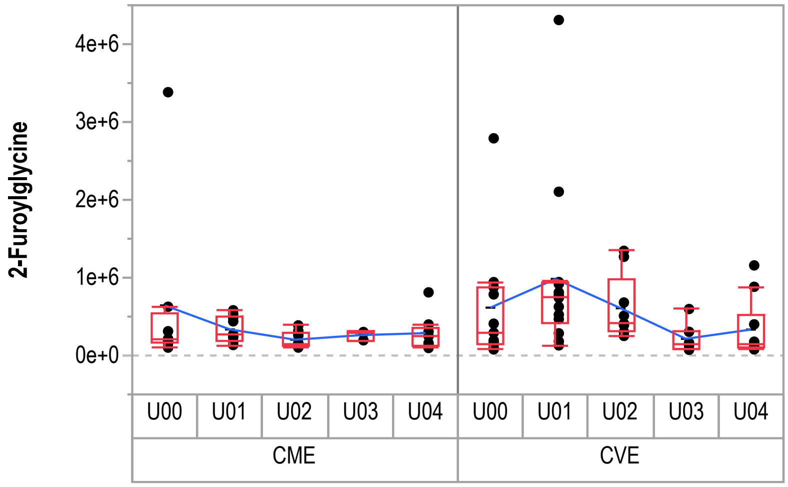

Variability Gauge

Variability Chart for 3,5-Dihydroxybenzoic acid

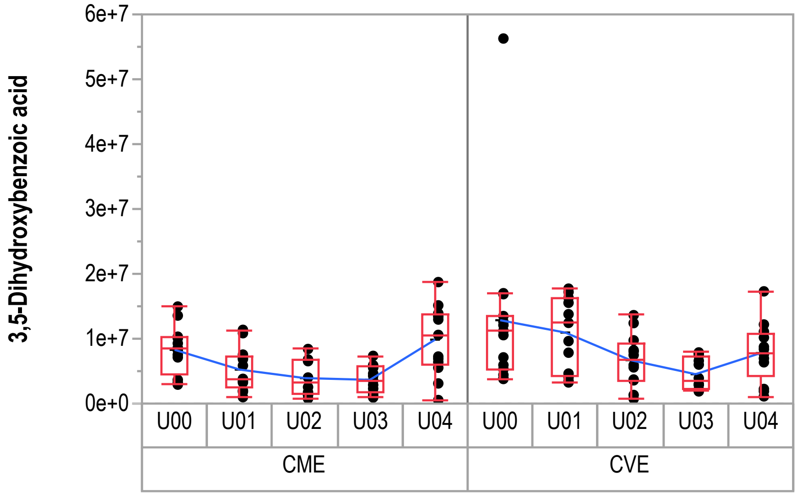

Variability Gauge

Variability Chart for 3-Deoxyhexonic acid

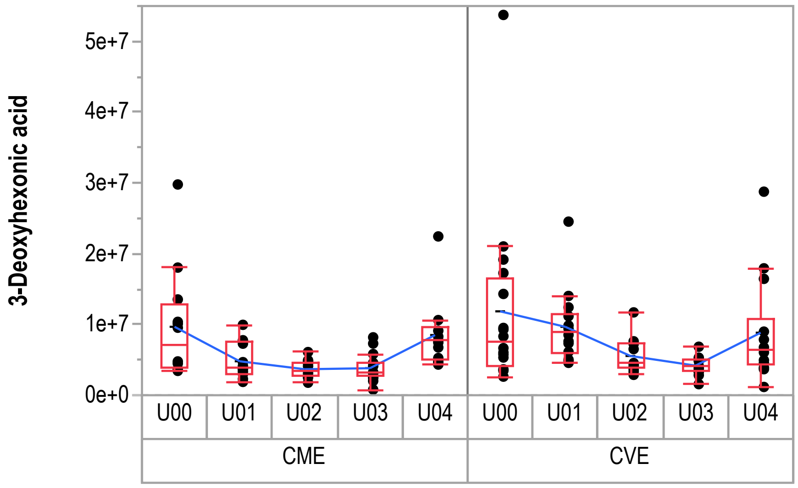

Variability Gauge

Variability Chart for 3-Indoxylsulfate

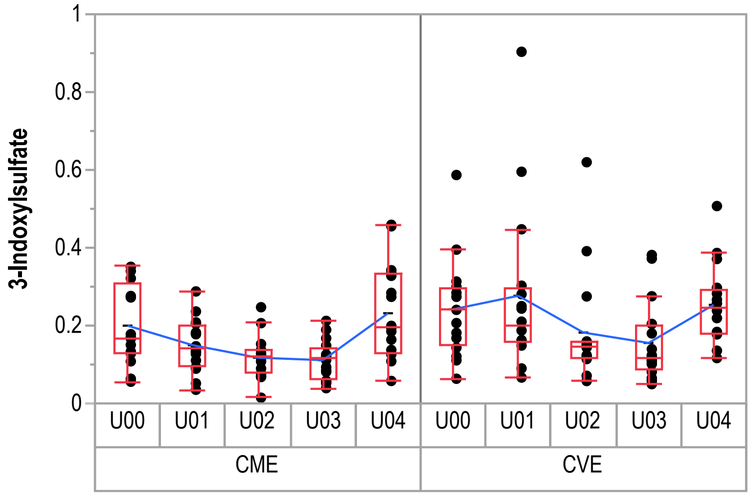

Variability Gauge

Variability Chart for 4-Hydroxyphenylacetate (GC×GC-MS)

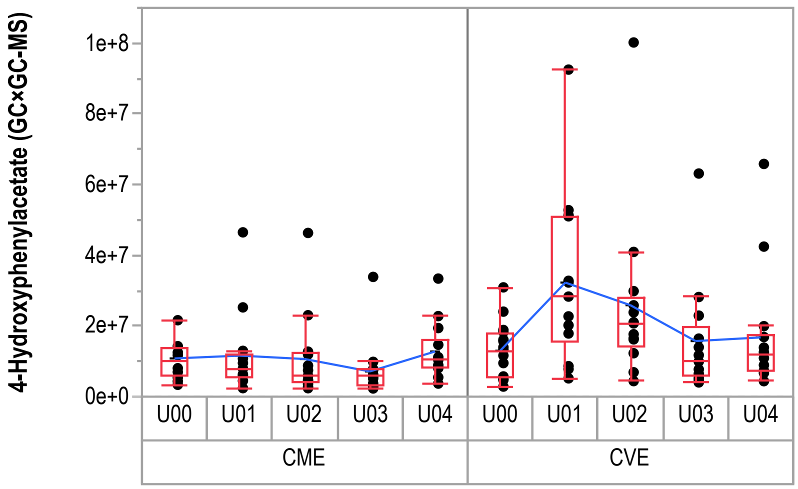

Variability Gauge

Variability Chart for 4-Hydroxyphenylacetate (NMR)

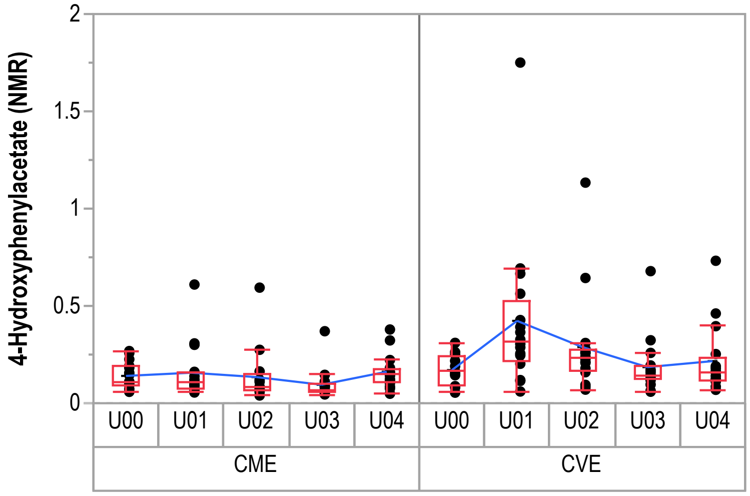

Variability Gauge

Variability Chart for 4-Hydroxyphenyllactic acid

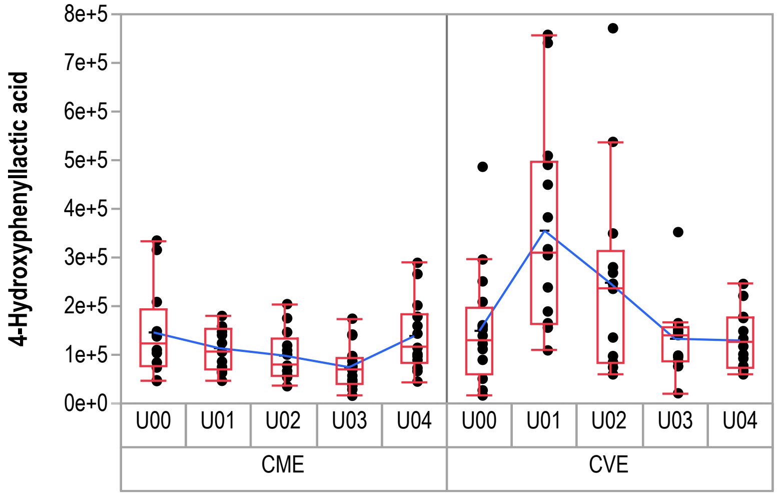

Variability Gauge

Variability Chart for 4-Hydroxyphenylpyruvic acid*

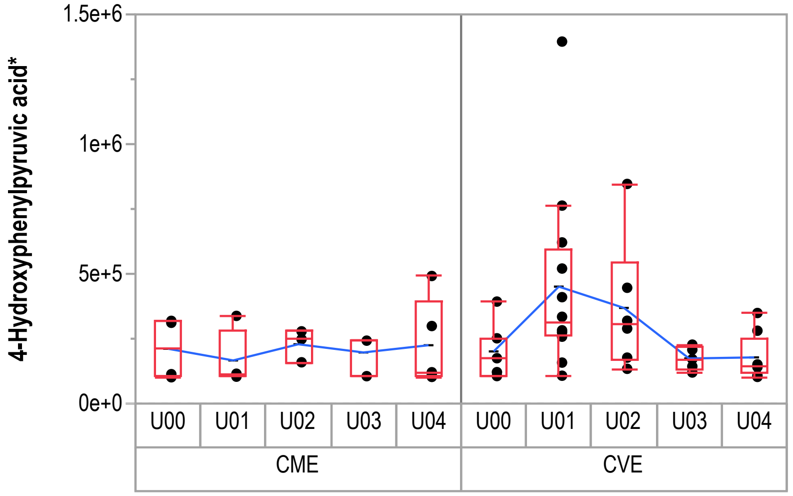

Variability Gauge

Variability Chart for 5-Hydroxy-3-indolacetic acid

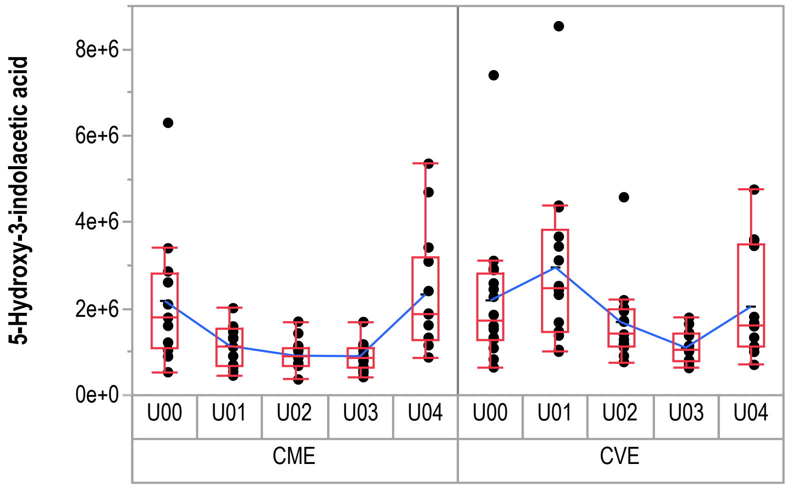

Variability Gauge

Variability Chart for 5-Hydroxymethyl-2-furoic acid*

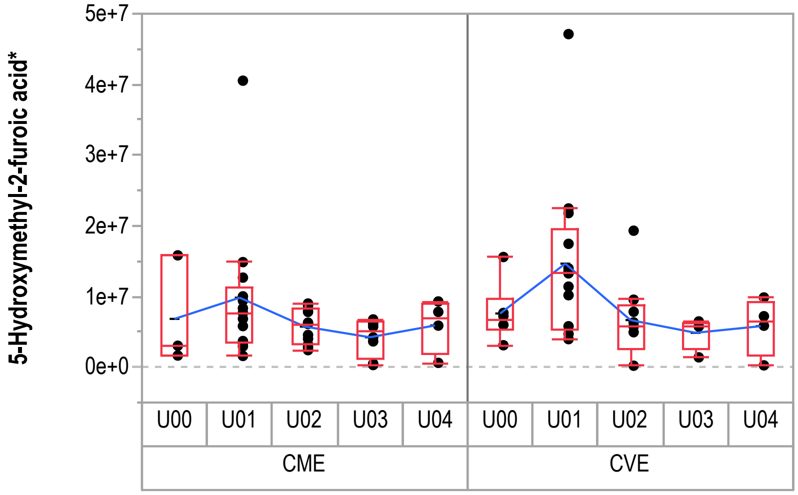

Variability Gauge

Variability Chart for 7-Methylxanthine

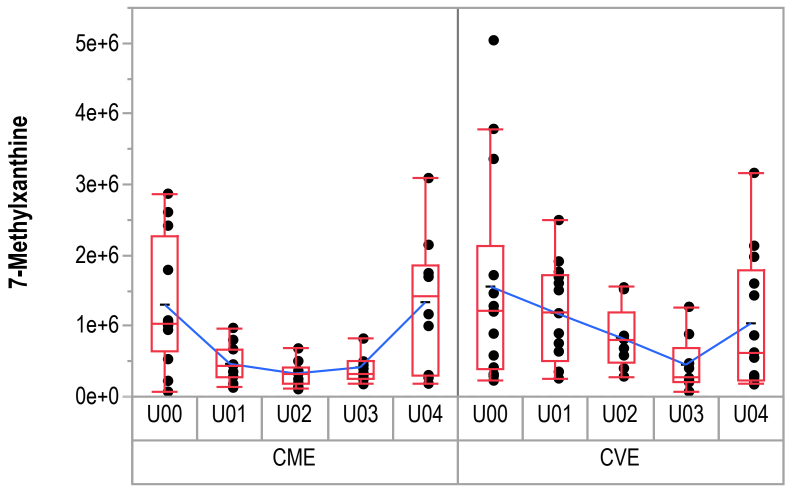

Variability Gauge

Variability Chart for Alanine (GC×GC-MS)

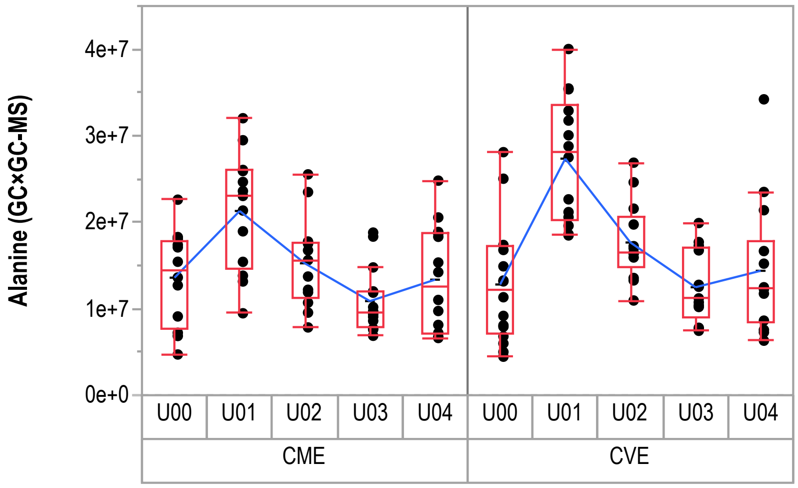

Variability Gauge

Variability Chart for Alanine (NMR)

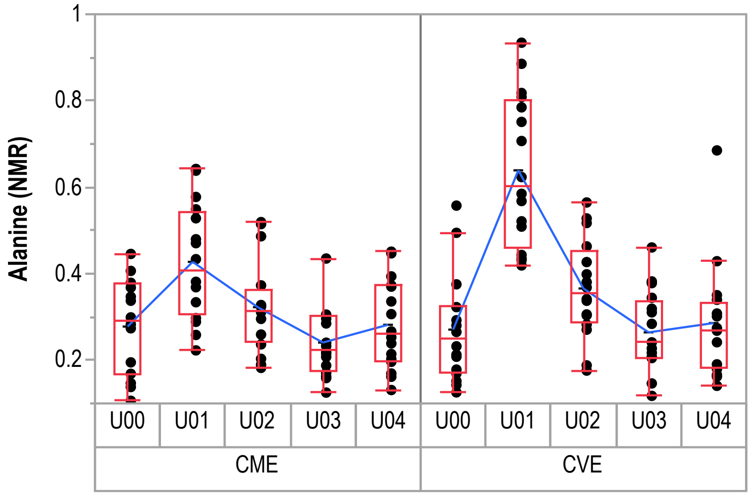

Variability Gauge

Variability Chart for Amino acid-like*

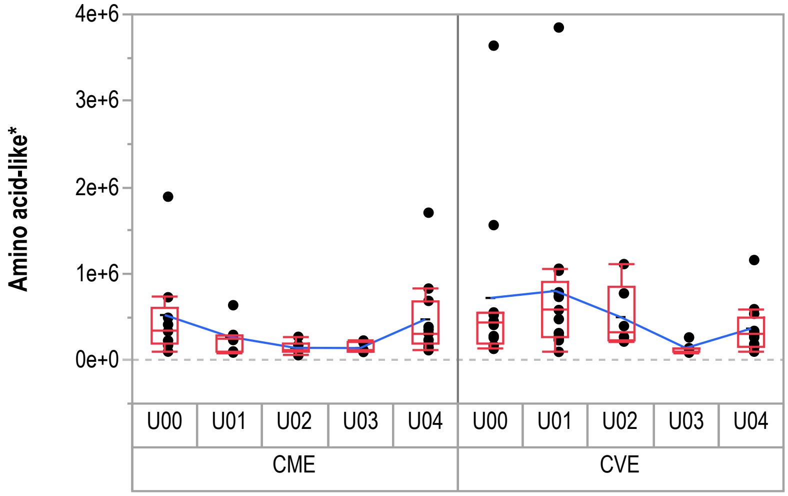

Variability Gauge

Variability Chart for Amino derivative

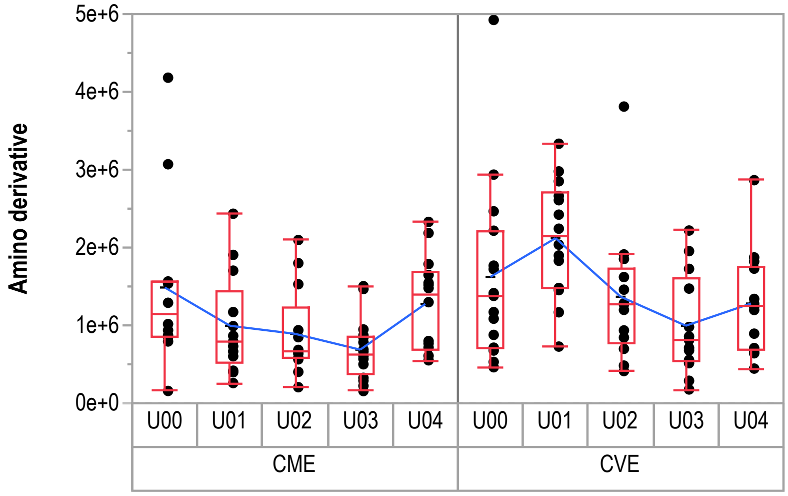

Variability Gauge

Variability Chart for C5-Sugar acid 1

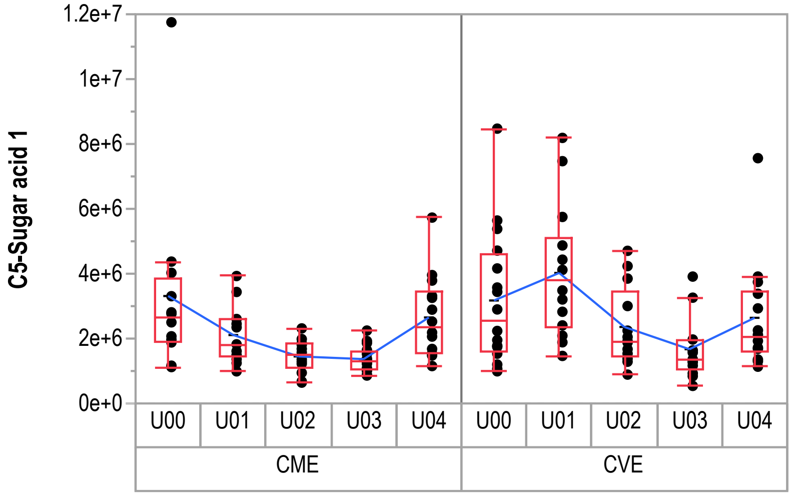

Variability Gauge

Variability Chart for C5-Sugar acid 2

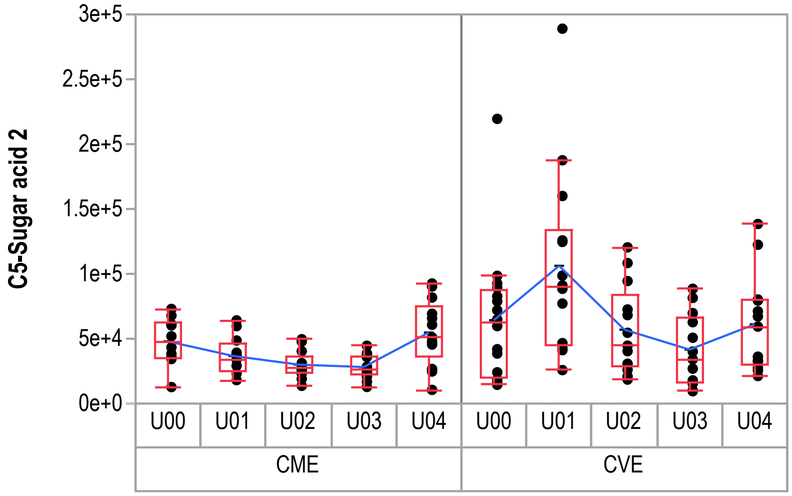

Variability Gauge

Variability Chart for C6-Sugar acid lactone*

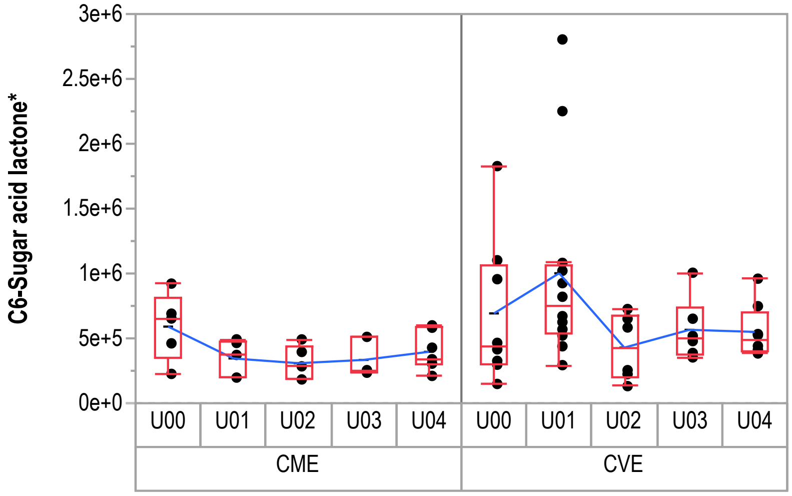

Variability Gauge

Variability Chart for Carnitine

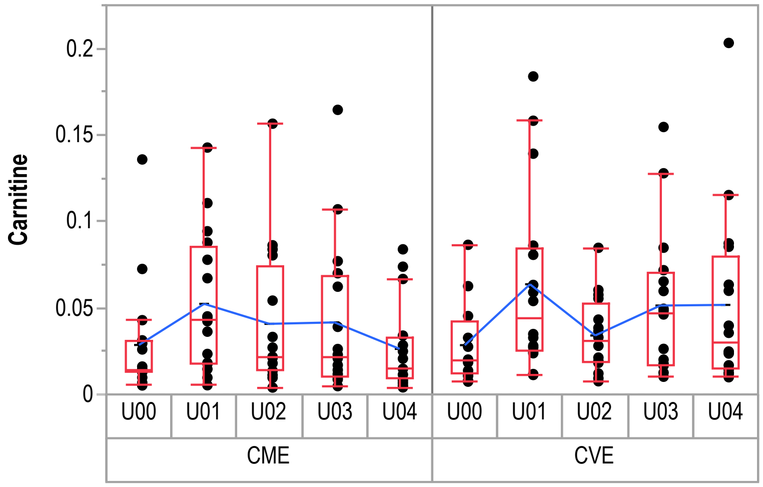

Variability Gauge

Variability Chart for cis-Aconitate (GC×GC-MS)

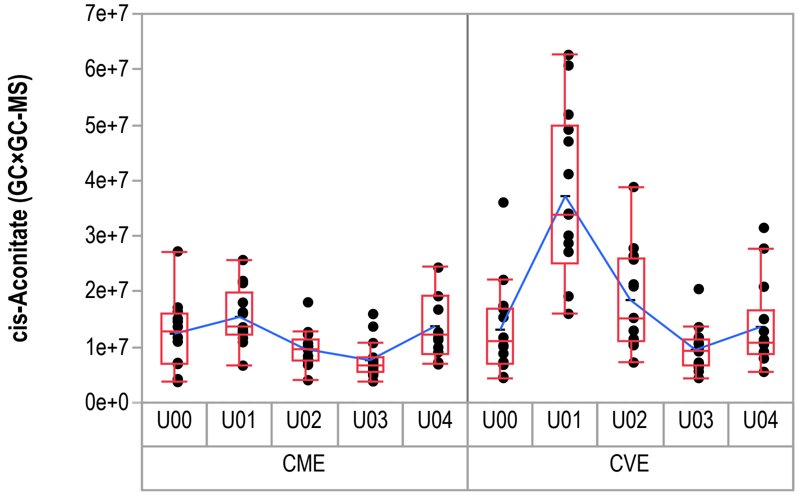

Variability Gauge

Variability Chart for cis-Aconitate (NMR)

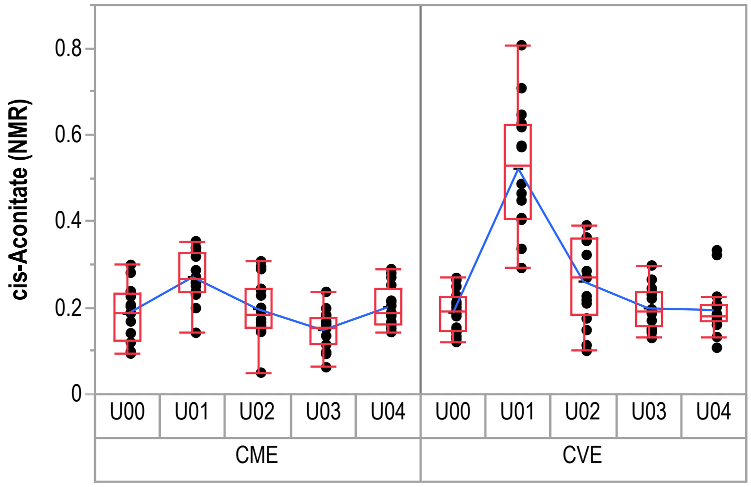

Variability Gauge

Variability Chart for Citrate

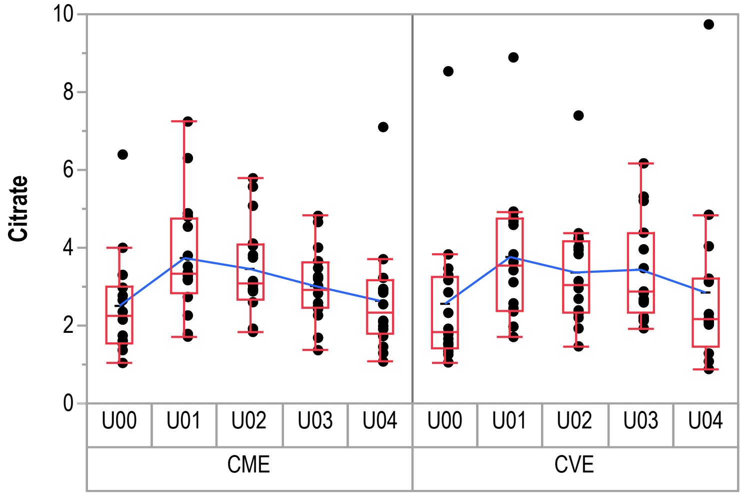

Variability Gauge

Variability Chart for Creatine

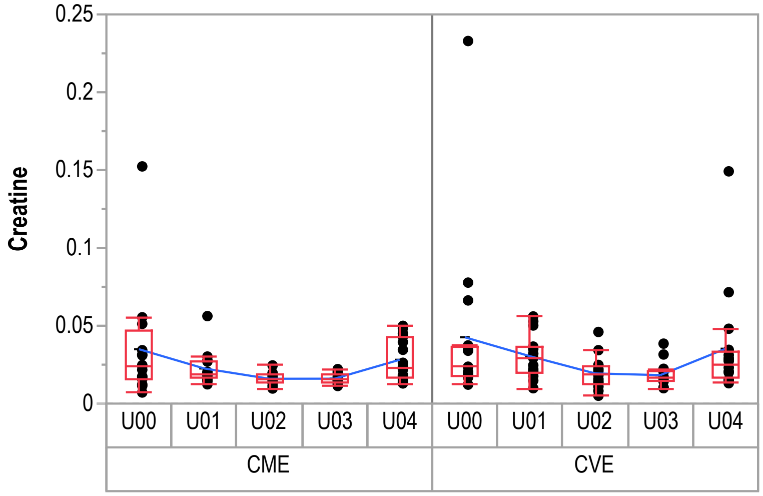

Variability Gauge

Variability Chart for Deoxymethyl-Sugar acid (C5)

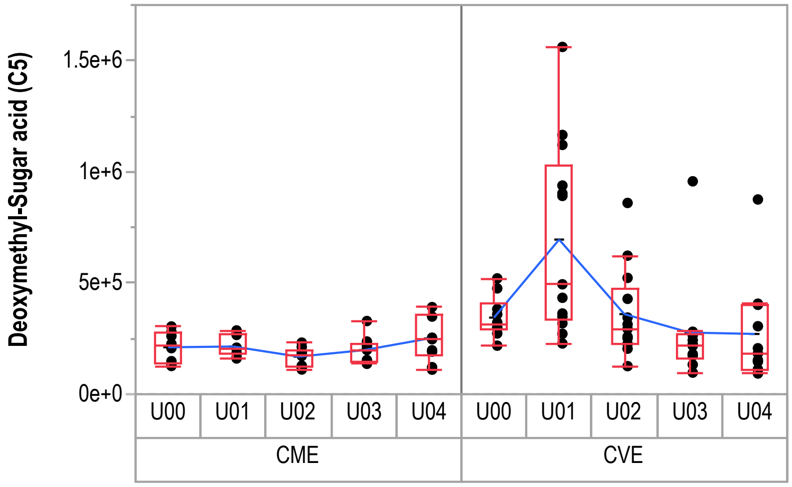

Variability Gauge

Variability Chart for Fructose

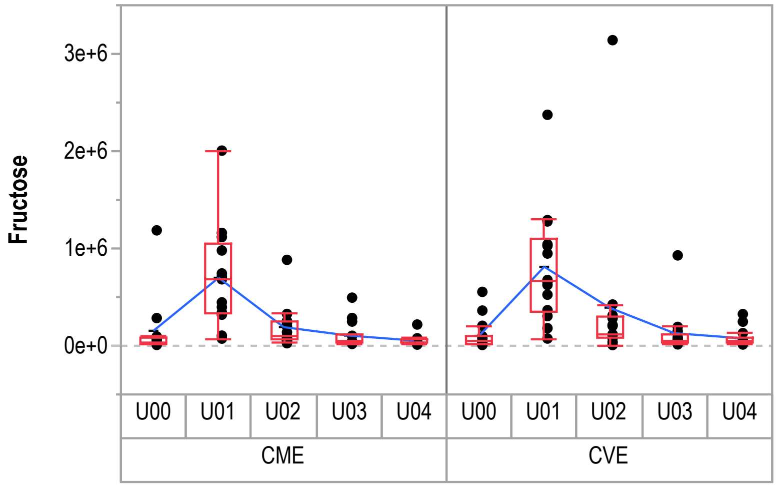

Variability Gauge

Variability Chart for Gluconate

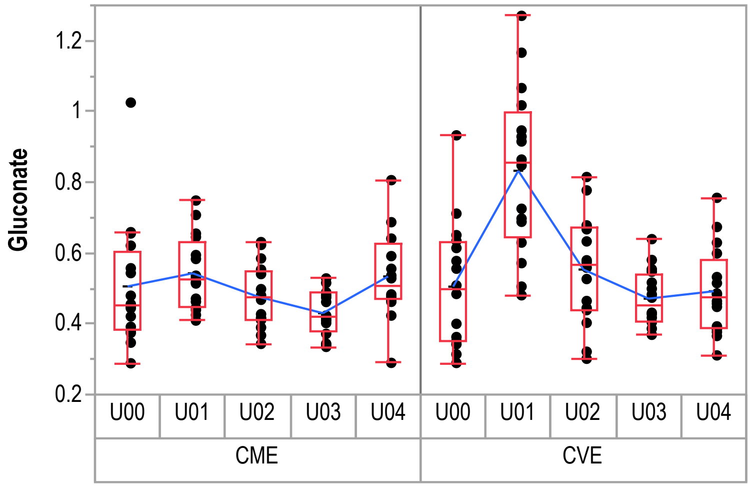

Variability Gauge

Variability Chart for Glucuronic acid

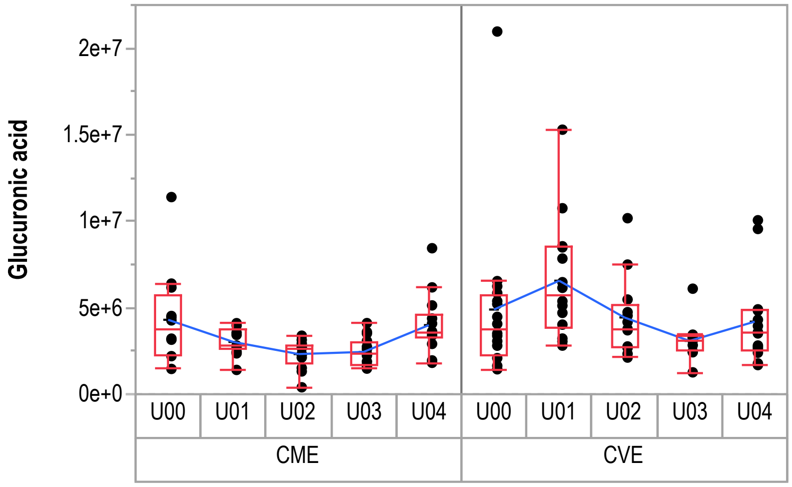

Variability Gauge

Variability Chart for Glutamic acid

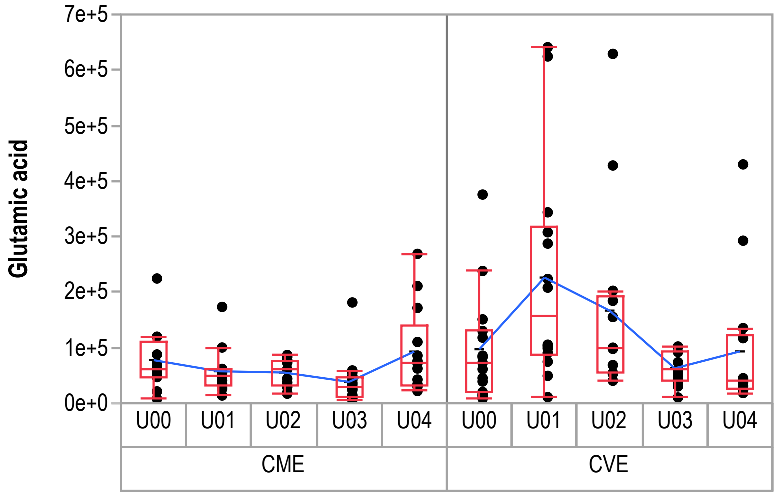

Variability Gauge

Variability Chart for Glycine

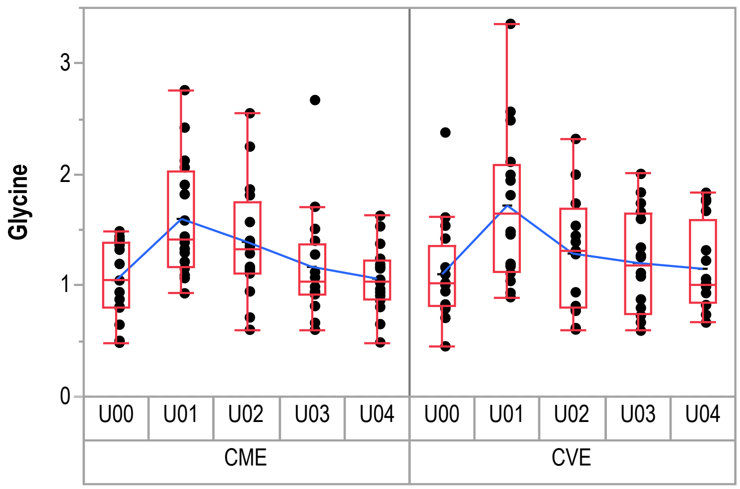

Variability Gauge

Variability Chart for Glycine-Proline

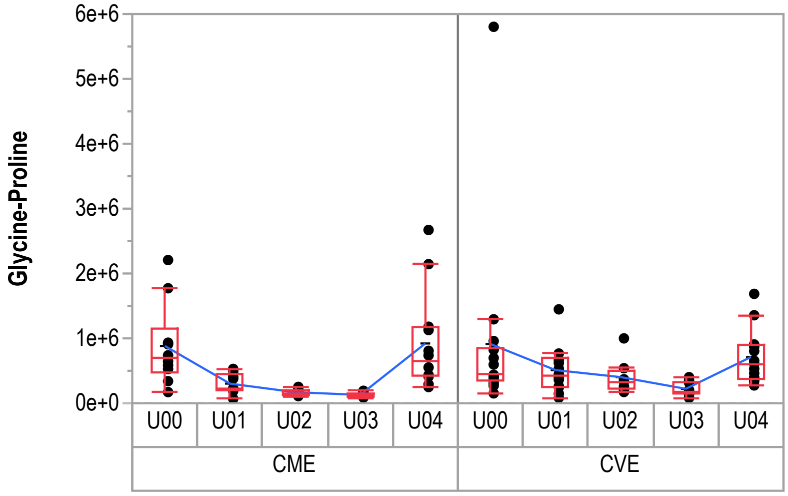

Variability Gauge

Variability Chart for Hippurate (GC×GC-MS)

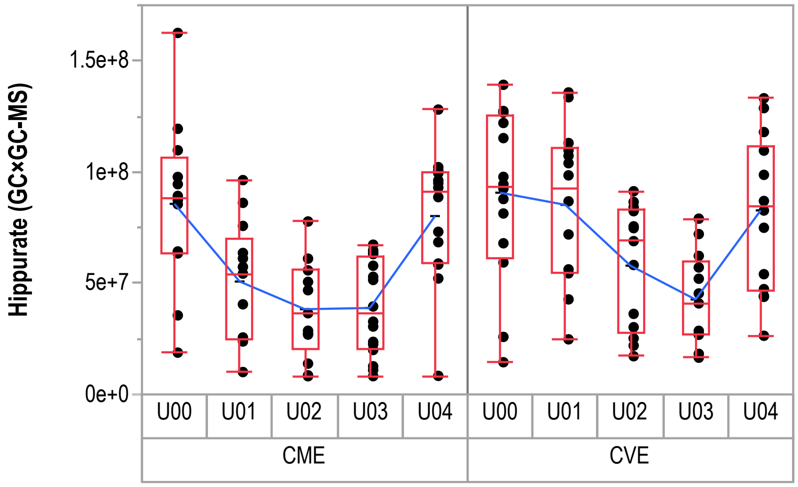

Variability Gauge

Variability Chart for Hippurate (NMR)

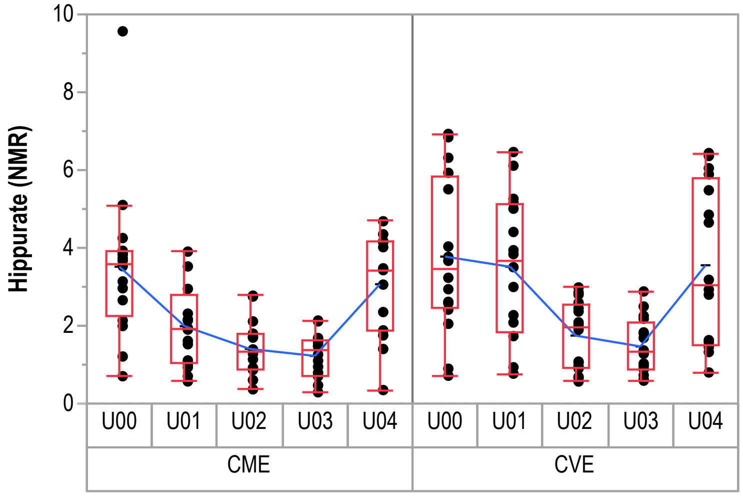

Variability Gauge

Variability Chart for Homovanillic acid

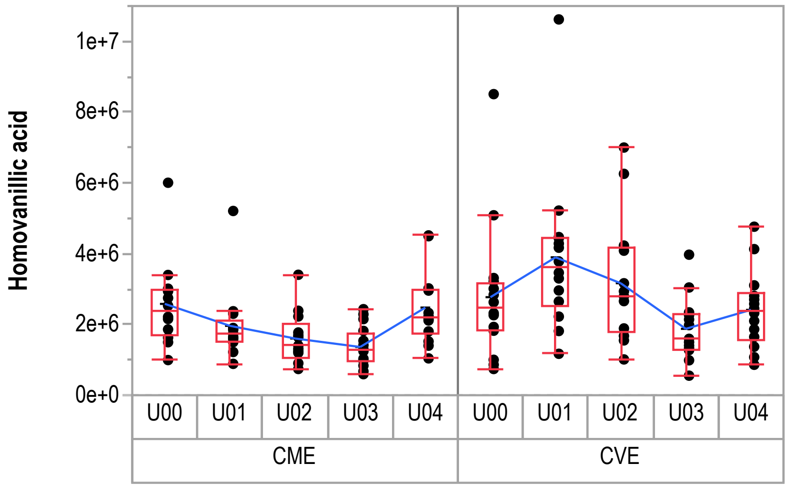

Variability Gauge

Variability Chart for Hydroxyproline

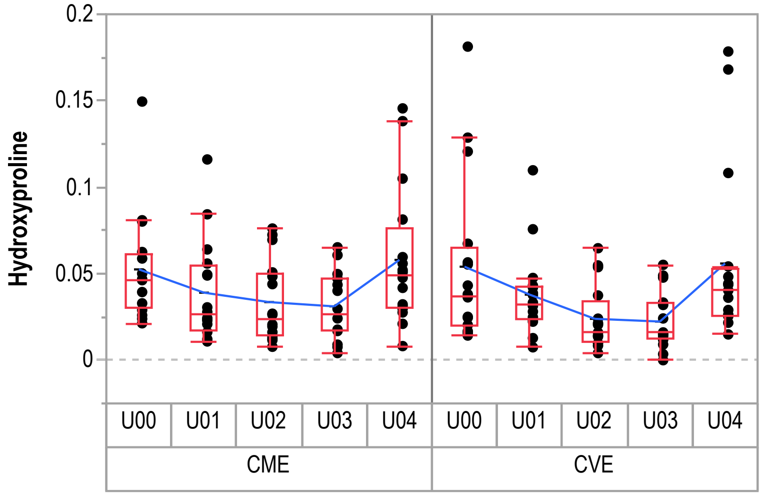

Variability Gauge

Variability Chart for Hypoxanthine (GC×GC-MS)

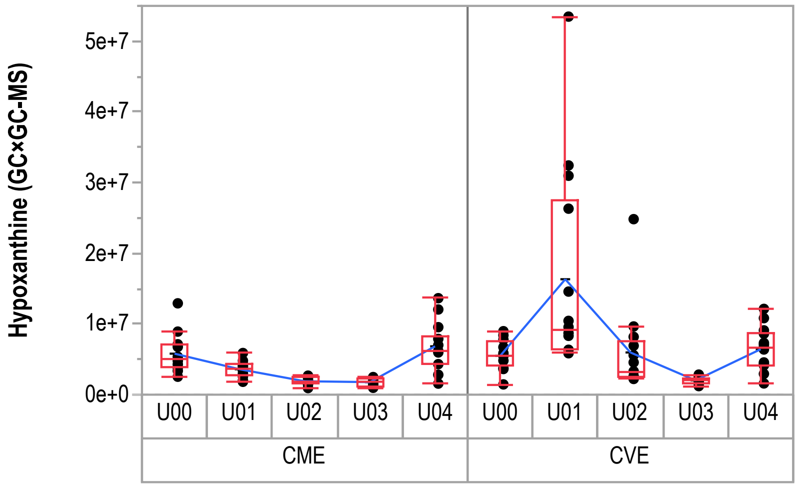

Variability Gauge

Variability Chart for Hypoxanthine (NMR)

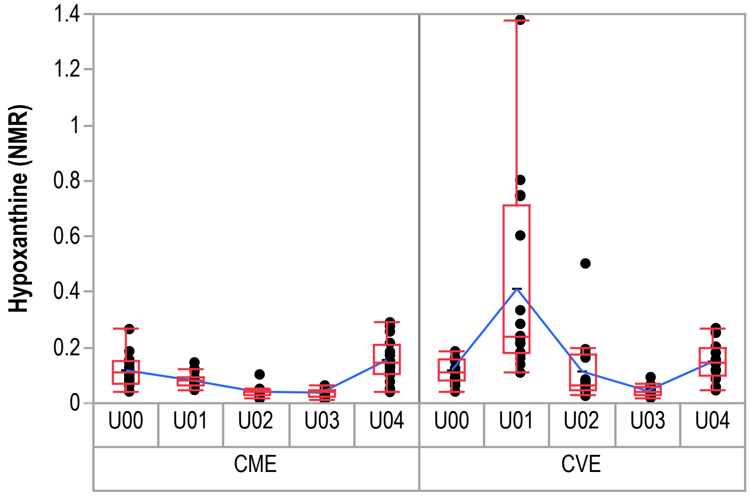

Variability Gauge

Variability Chart for Indol-3-carboxylic acid

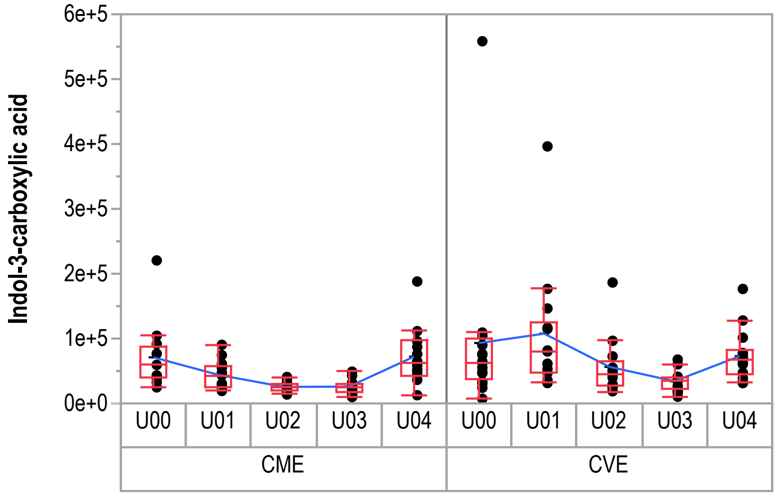

Variability Gauge

Variability Chart for Isomaltose*

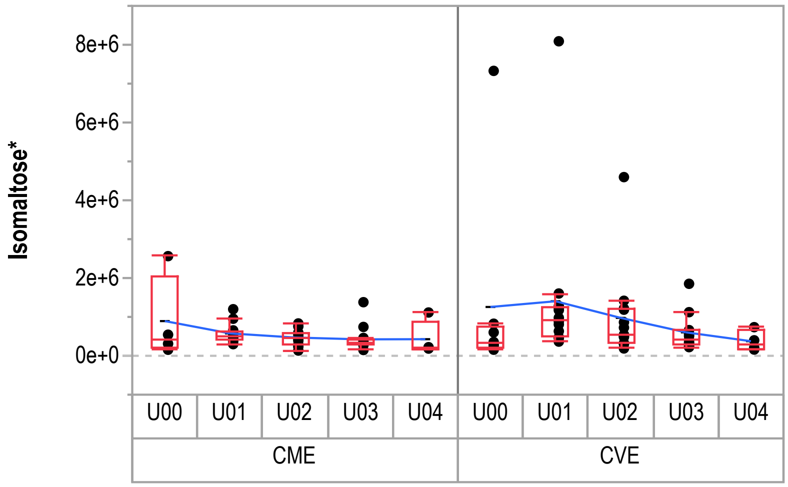

Variability Gauge

Variability Chart for Lactate (GC×GC-MS)

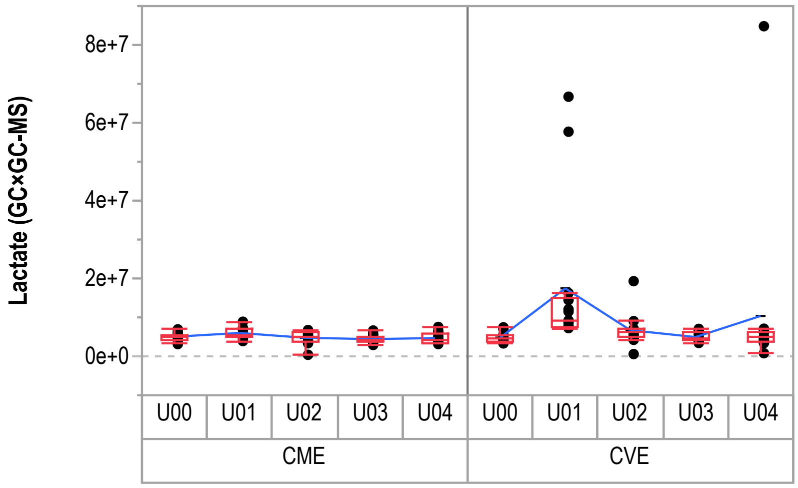

Variability Gauge

Variability Chart for Lactate (NMR)

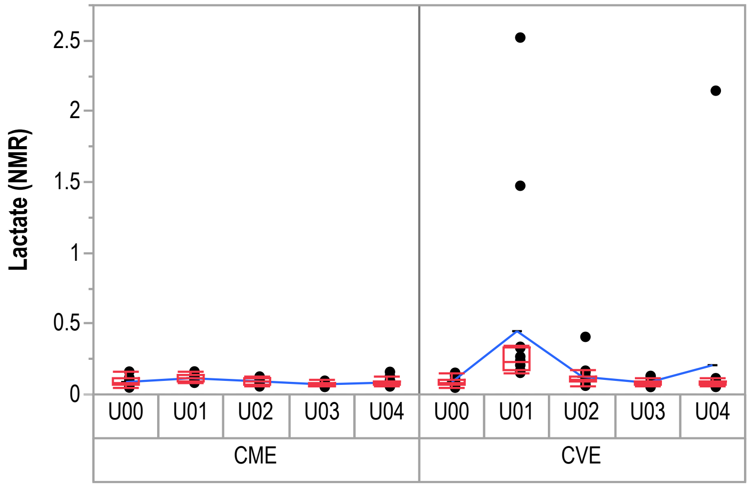

Variability Gauge

Variability Chart for Lactose

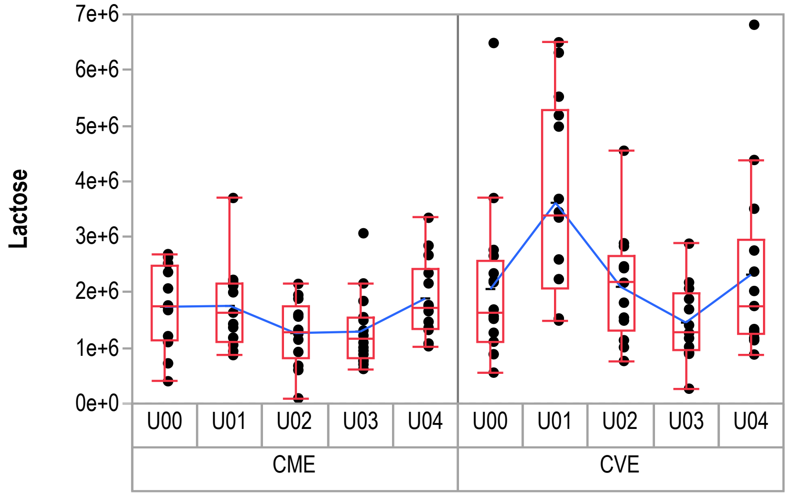

Variability Gauge

Variability Chart for Malic acid

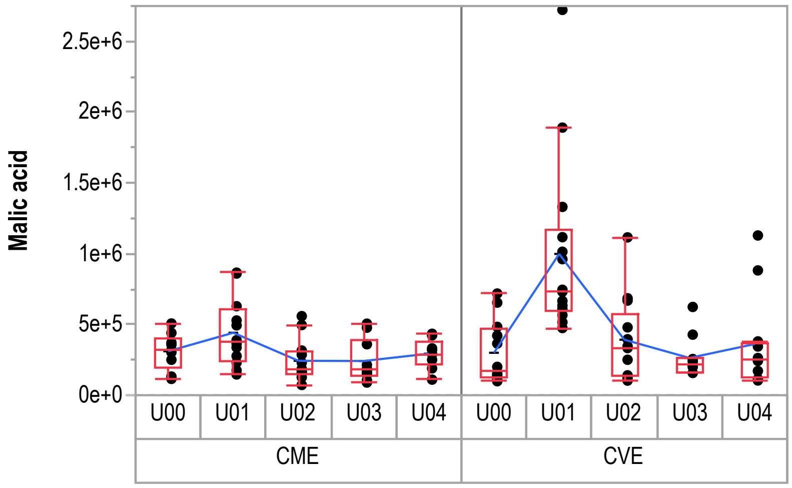

Variability Gauge

Variability Chart for Maltol*

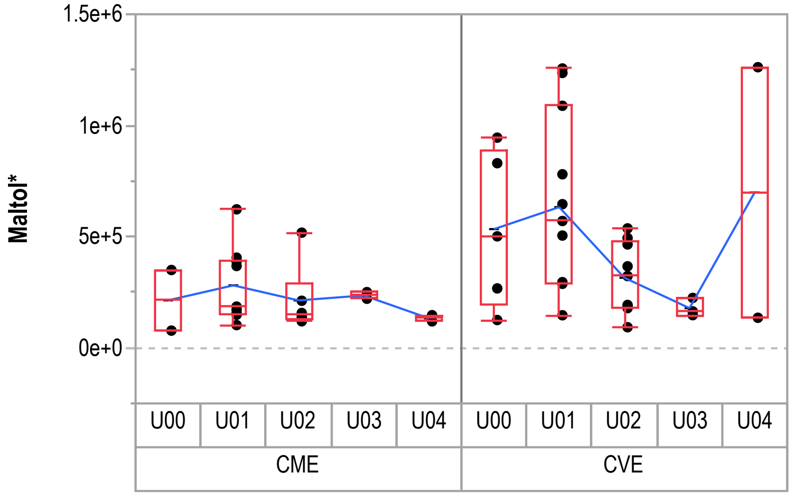

Variability Gauge

Variability Chart for Mannitol and Sorbitol

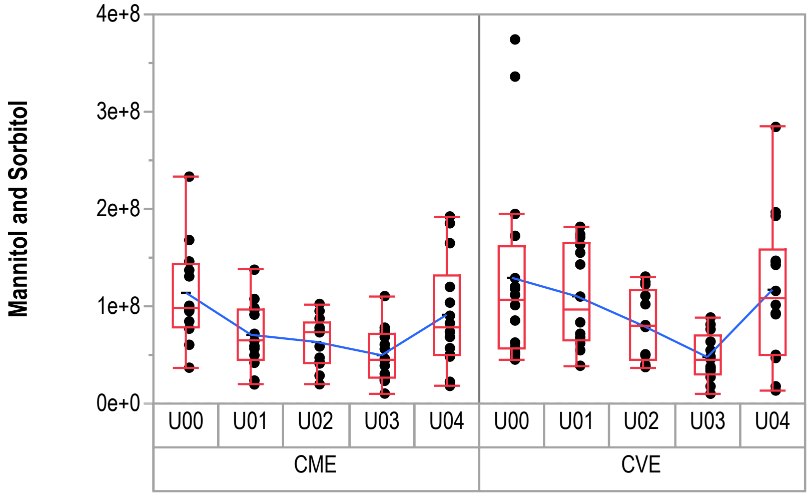

Variability Gauge

Variability Chart for Mannose

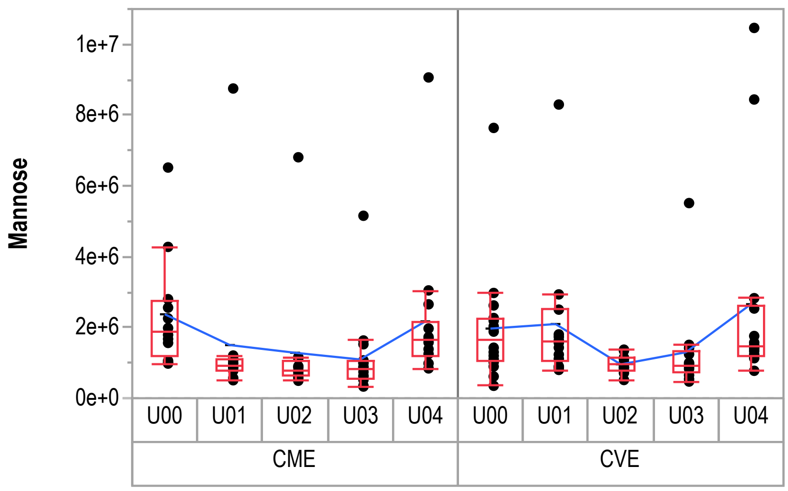

Variability Gauge

Variability Chart for Methylsuccinate

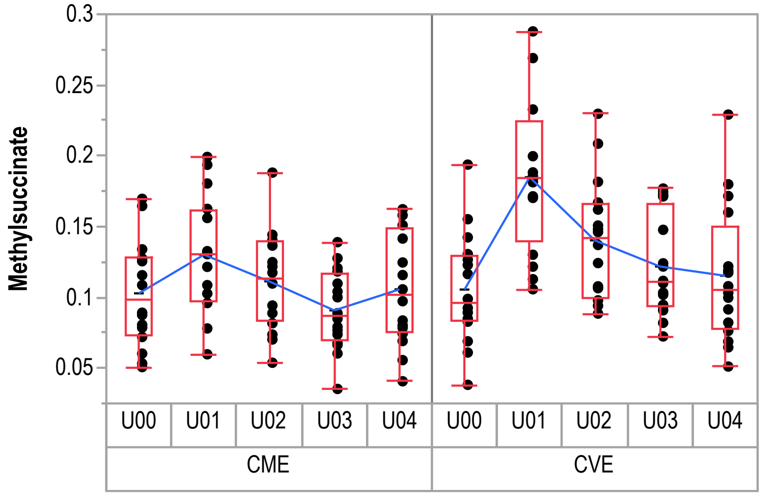

Variability Gauge

Variability Chart for N,N-Dimethylglycine

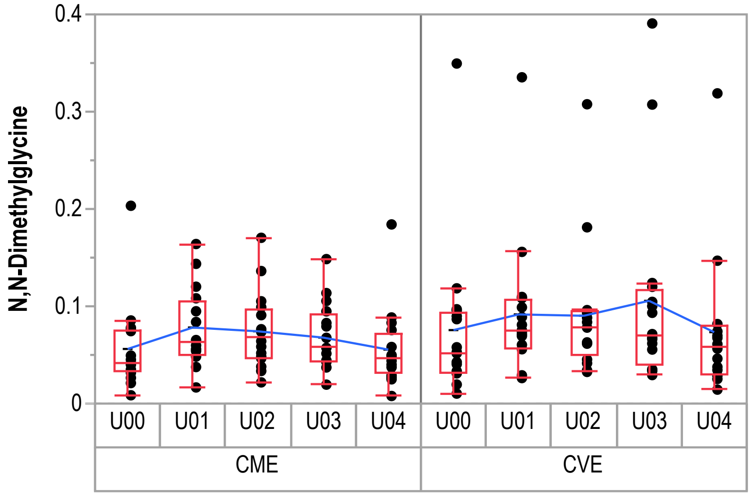

Variability Gauge

Variability Chart for N-Acetyl-D-gluco- and -mannosamine

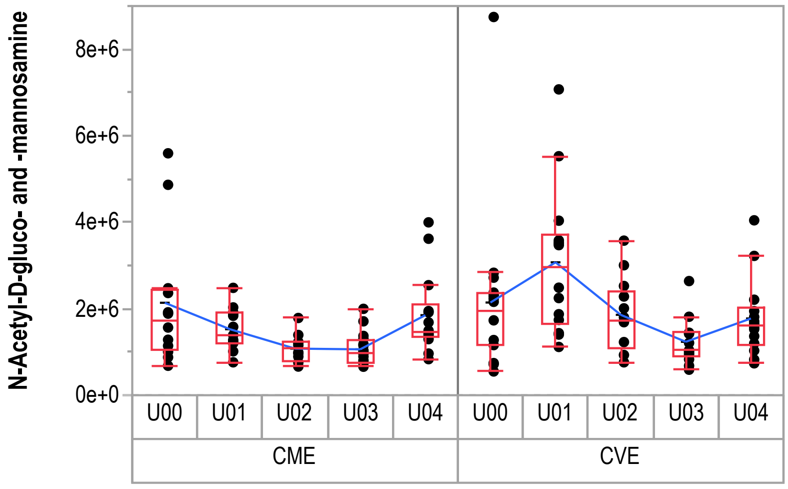

Variability Gauge

Variability Chart for N-Acetyl-D-hexosaminitol

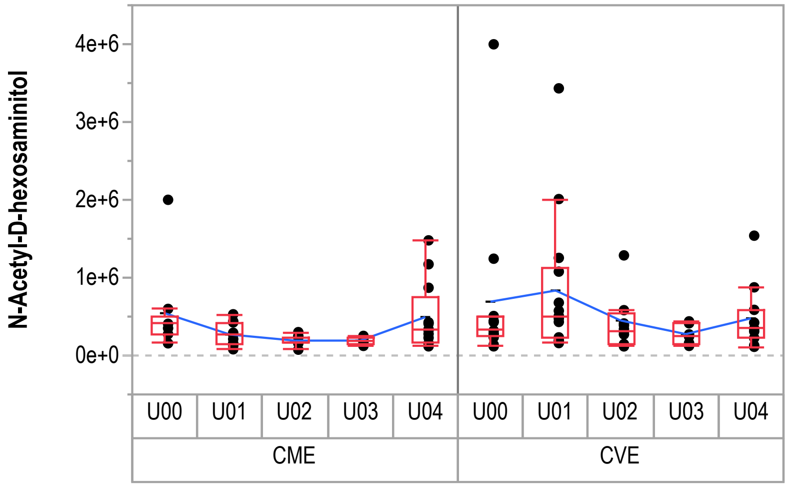

Variability Gauge

Variability Chart for N-Acetyl-D-mannosamine*

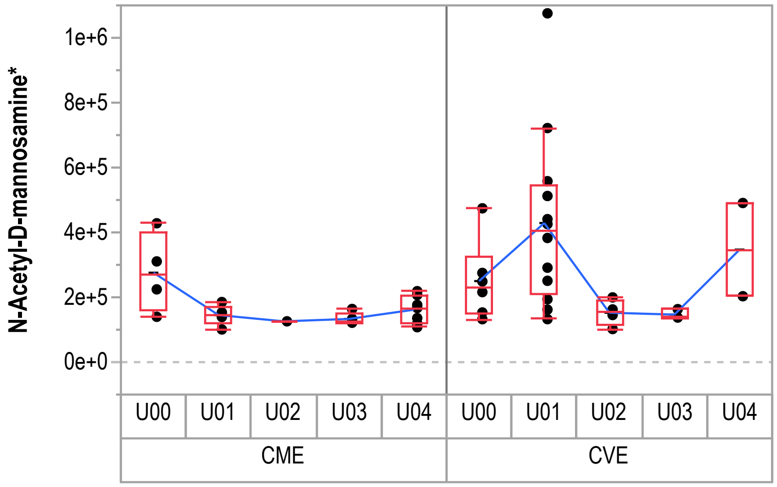

Variability Gauge

Variability Chart for N-carbamyl-L-glutamic acid

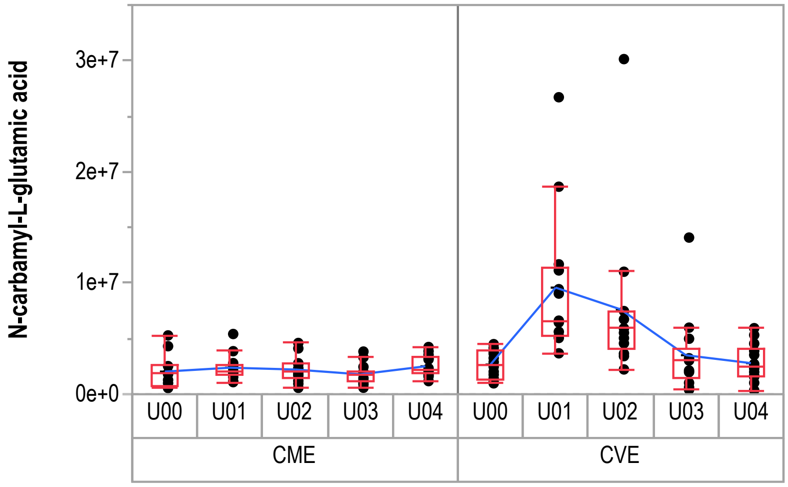

Variability Gauge

Variability Chart for N-Methylproline

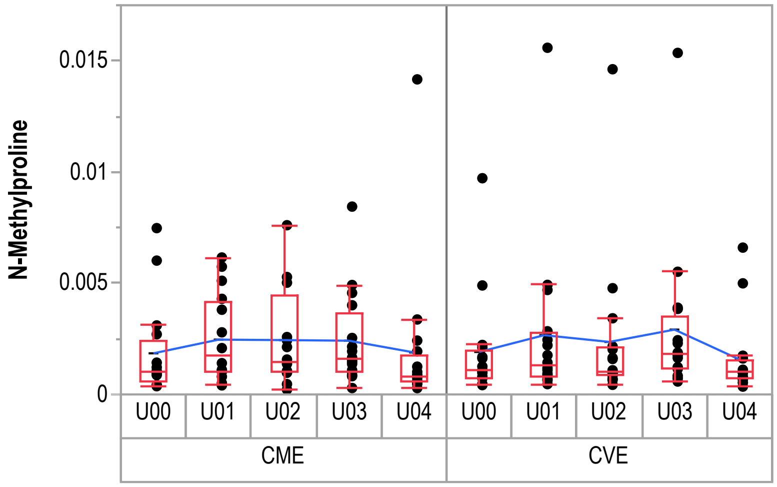

Variability Gauge

Variability Chart for Pantothenic acid

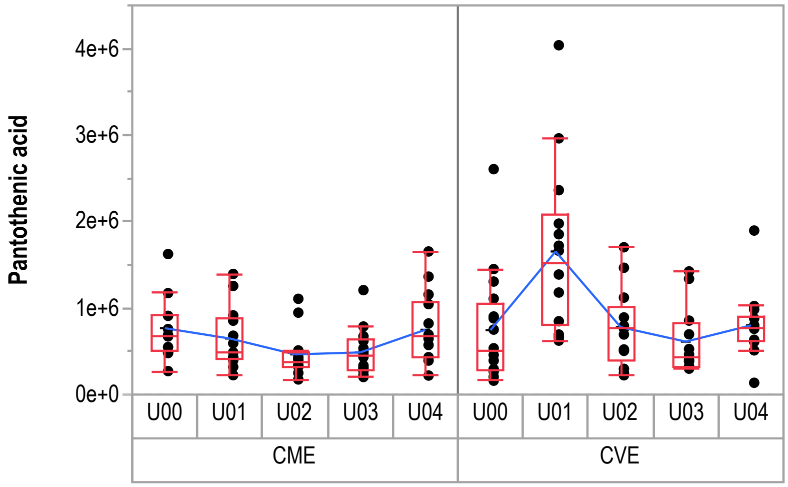

Variability Gauge

Variability Chart for Pyruvate

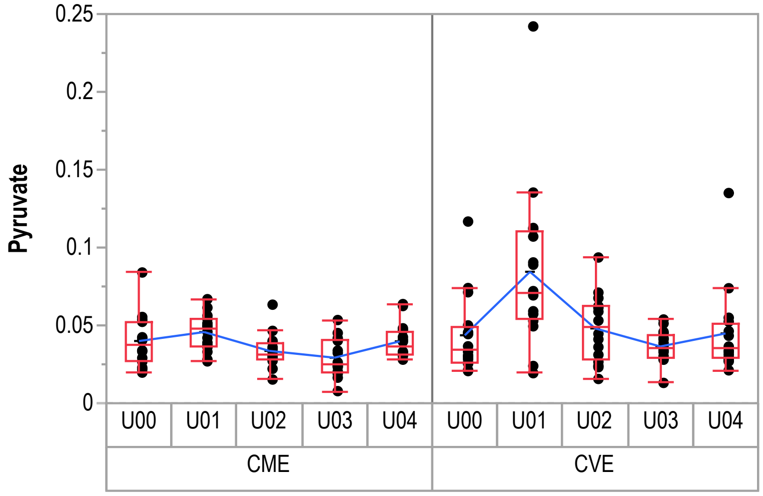

Variability Gauge

Variability Chart for Resorcinol and Mesaconic acid

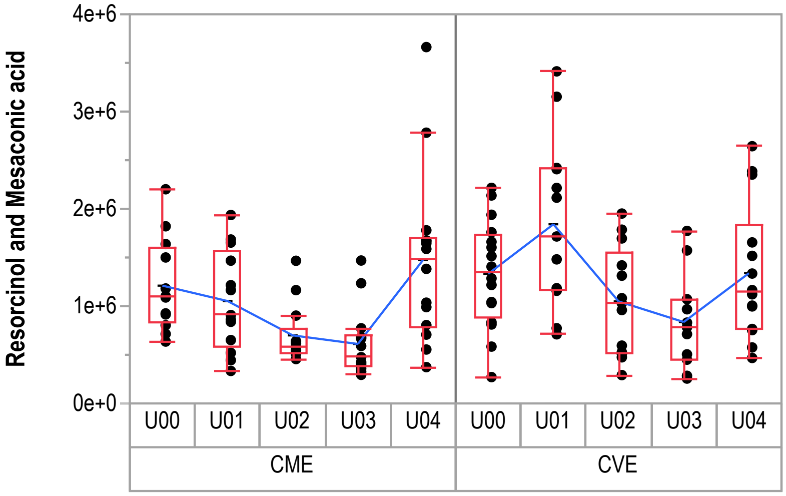

Variability Gauge

Variability Chart for Ribonic acid-gamma-lactone*

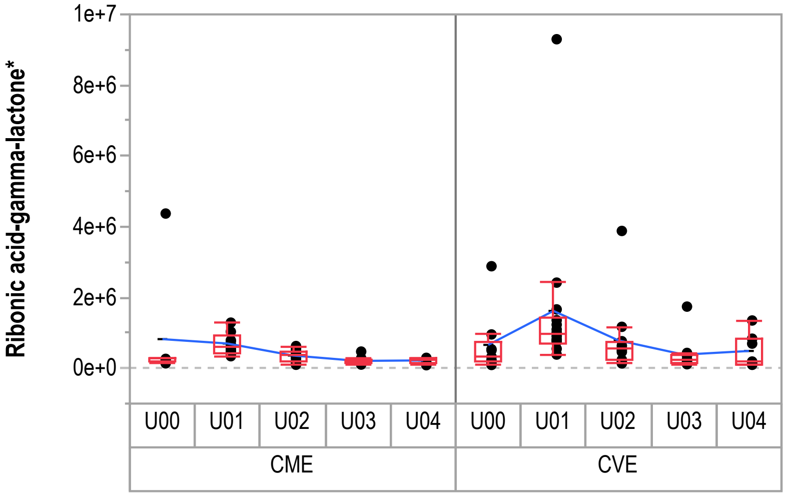

Variability Gauge

Variability Chart for Similar to 1,5-Anhydrosorbitol

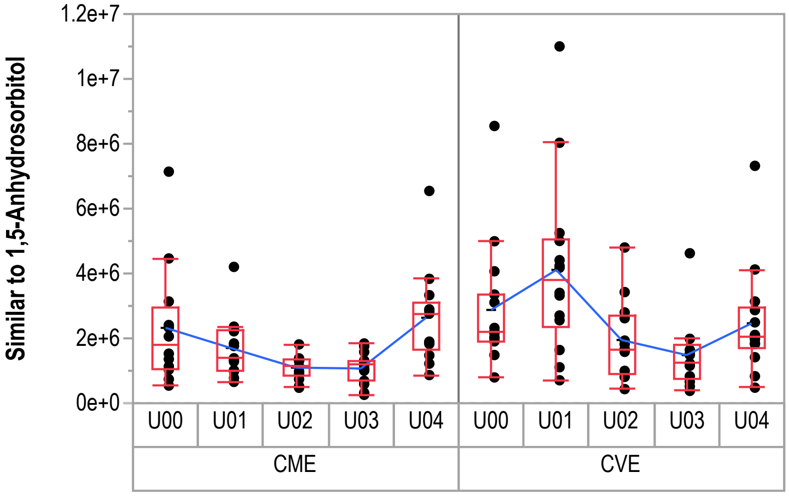

Variability Gauge

Variability Chart for Similar to 3',5'-Dihydroxyflavon

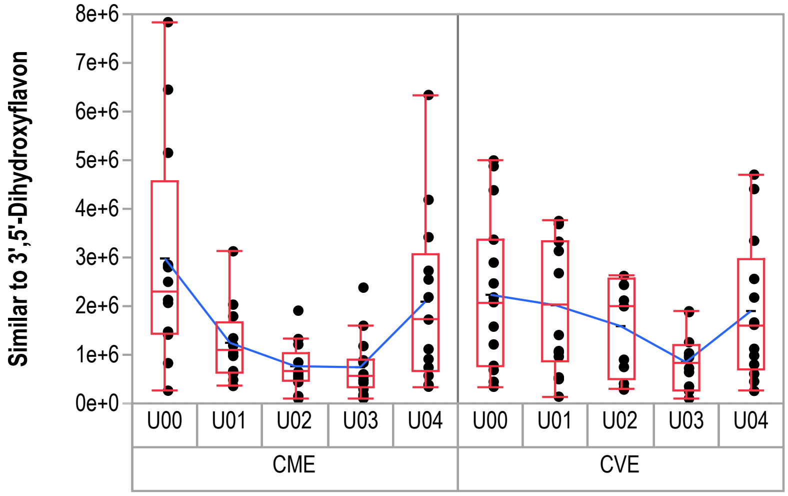

Variability Gauge

Variability Chart for Similar to α-Hydroxyglutaric acid

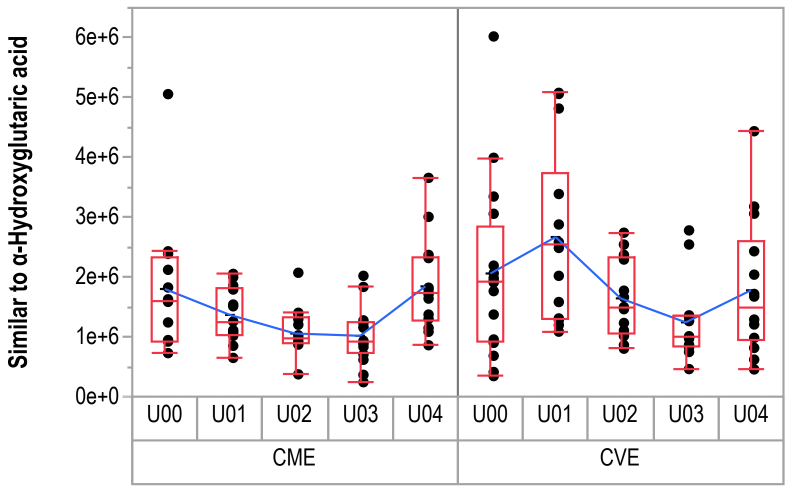

Variability Gauge

Variability Chart for Sugar-like 1

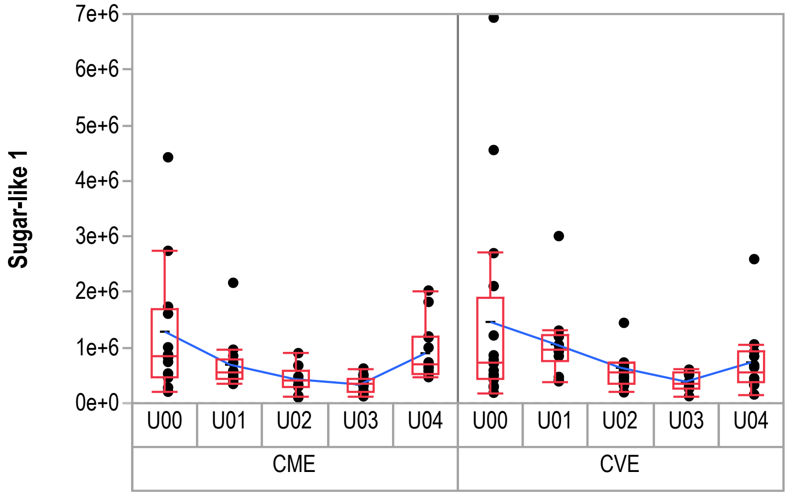

Variability Gauge

Variability Chart for Sugar-like 2

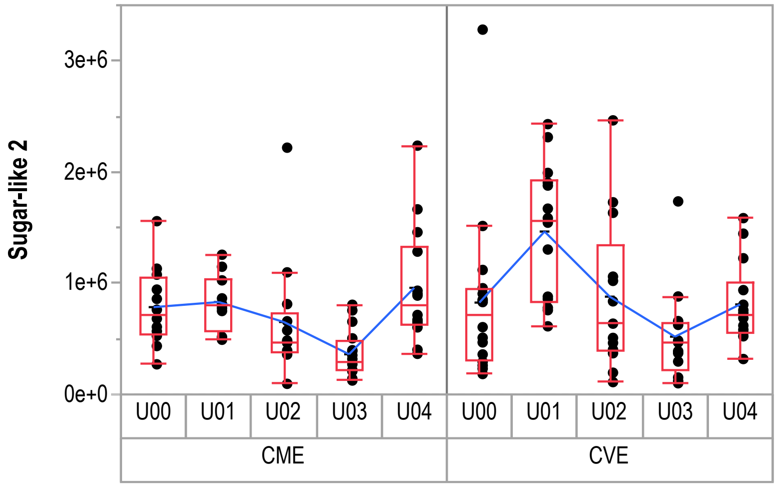

Variability Gauge

Variability Chart for Sugar-like 3

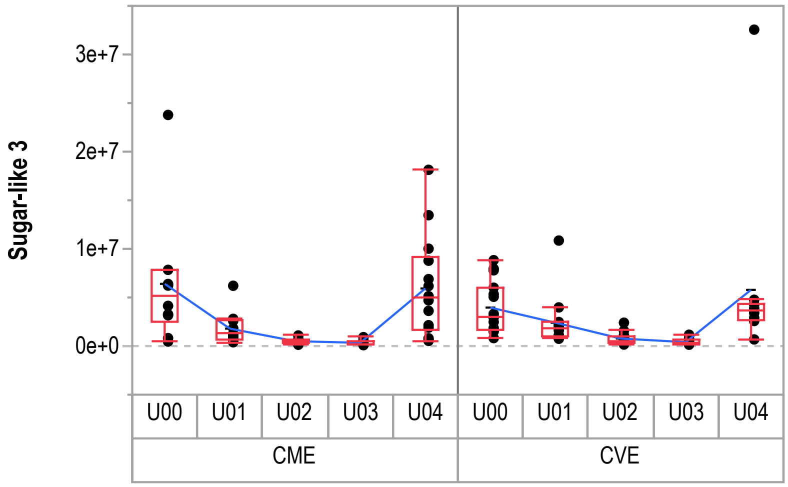

Variability Gauge

Variability Chart for Sugar-like 4

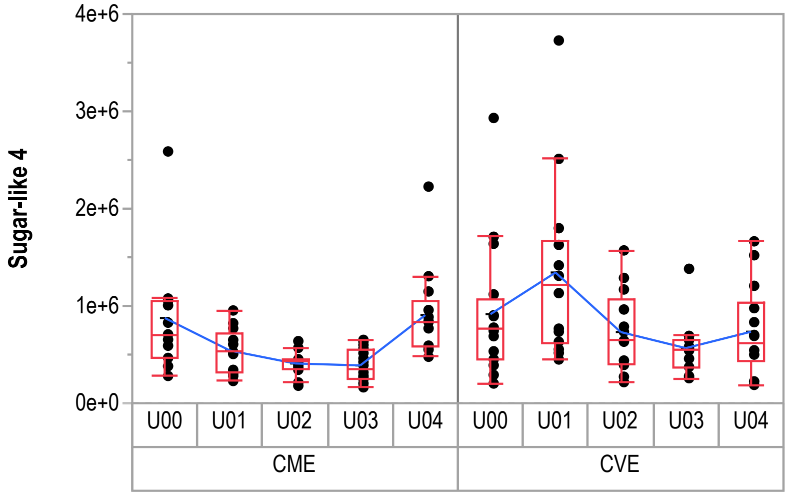

Variability Gauge

Variability Chart for Taurine

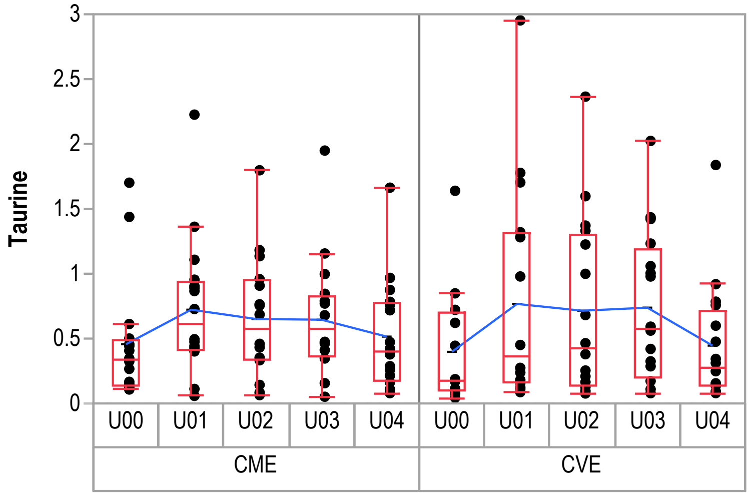

Variability Gauge

Variability Chart for Threonine

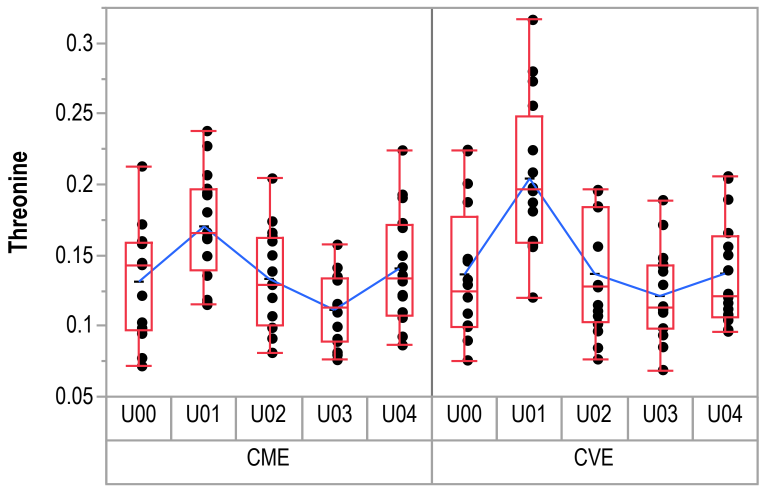

Variability Gauge

Variability Chart for Tiglylglycine

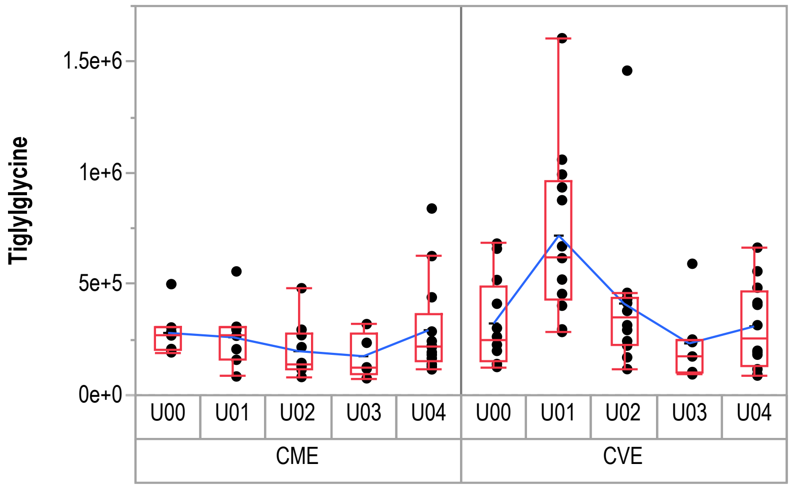

Variability Gauge

Variability Chart for trans-Aconitate

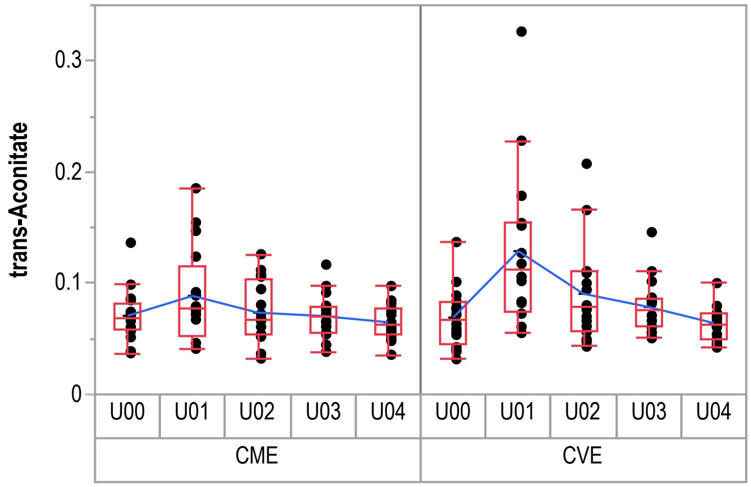

Variability Gauge

Variability Chart for Trigonelline

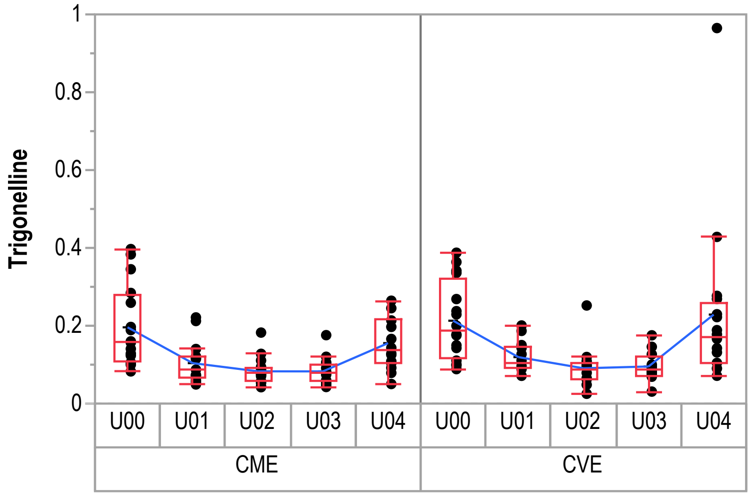

Variability Gauge

Variability Chart for Tryptophan

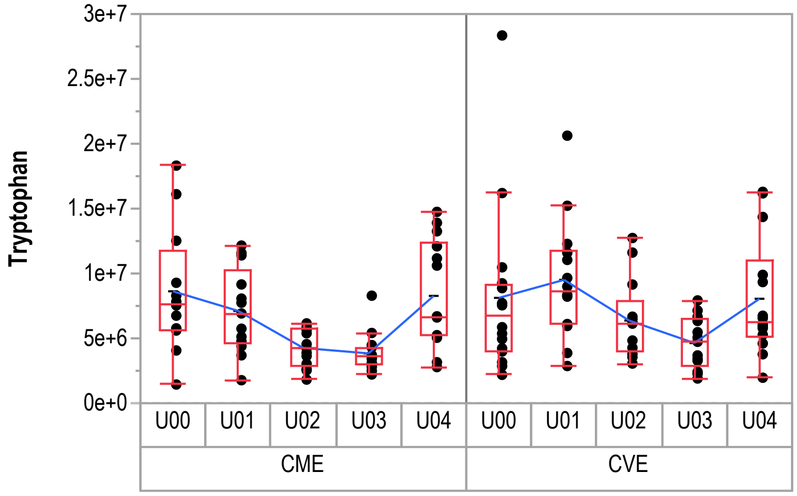

Variability Gauge

Variability Chart for Tyrosine

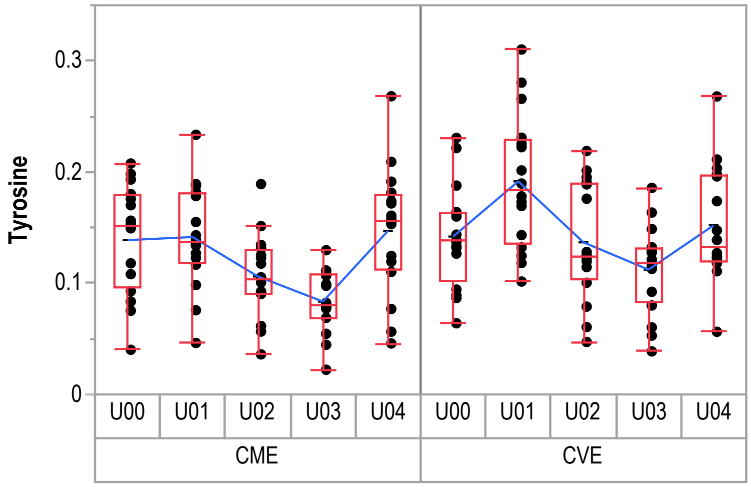

Variability Gauge

Variability Chart for Unknown 0089*

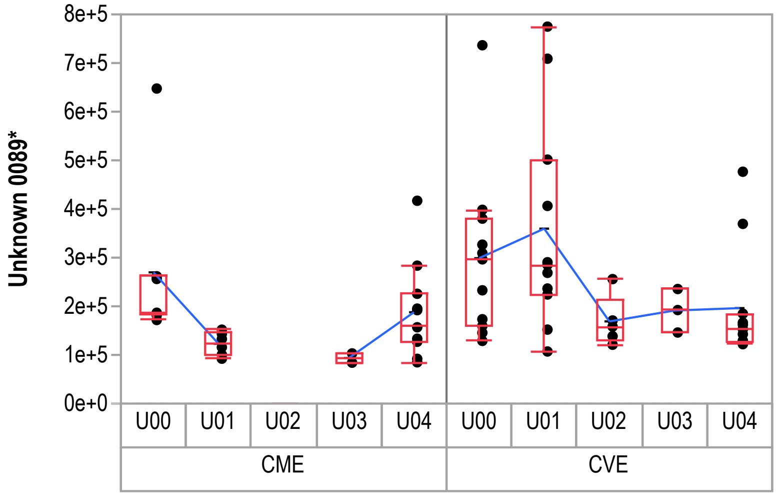

Variability Gauge

Variability Chart for Unknown 0231

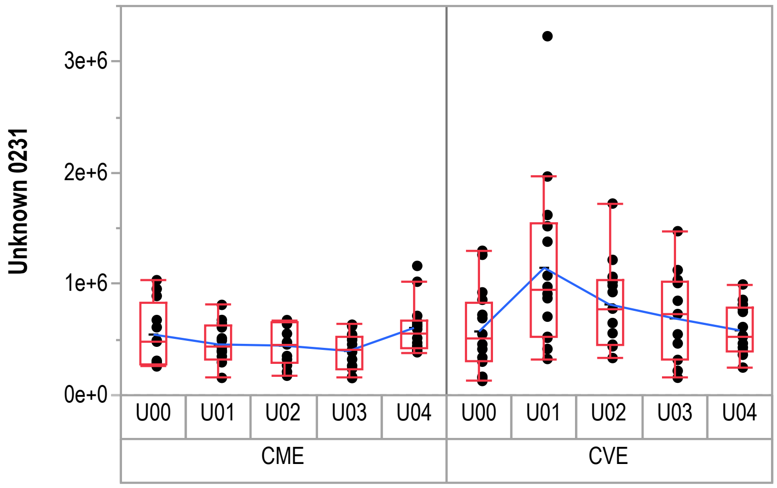

Variability Gauge

Variability Chart for Unknown 0262

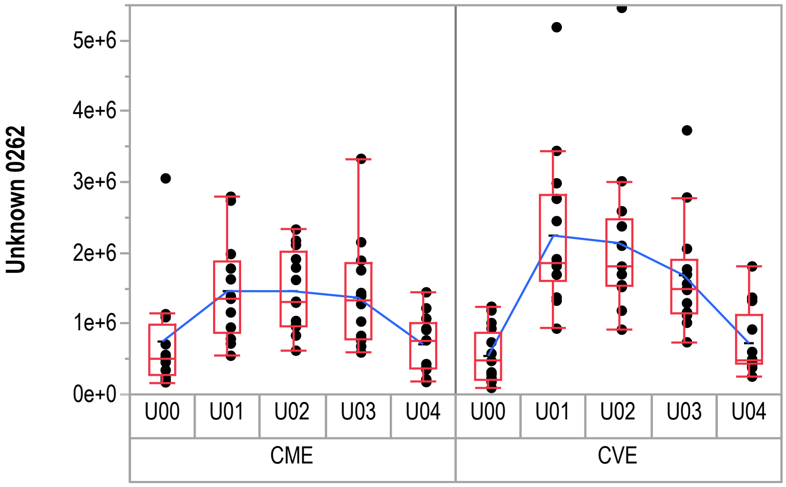

Variability Gauge

Variability Chart for Unknown 0274

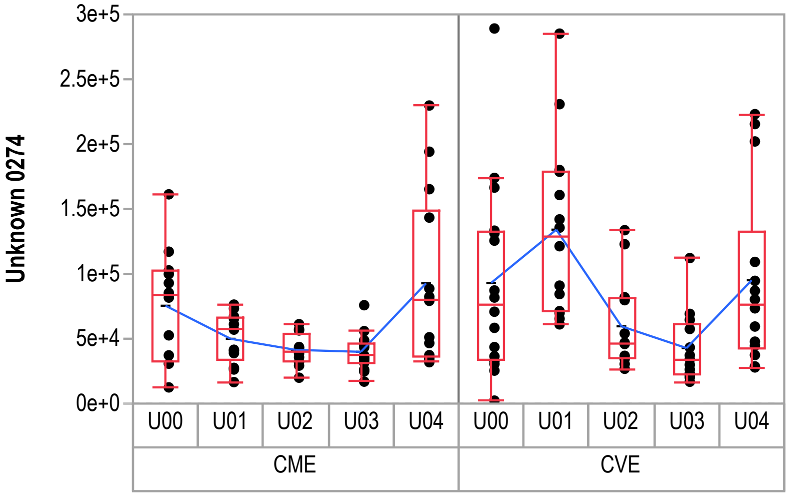

Variability Gauge

Variability Chart for Unknown 0339

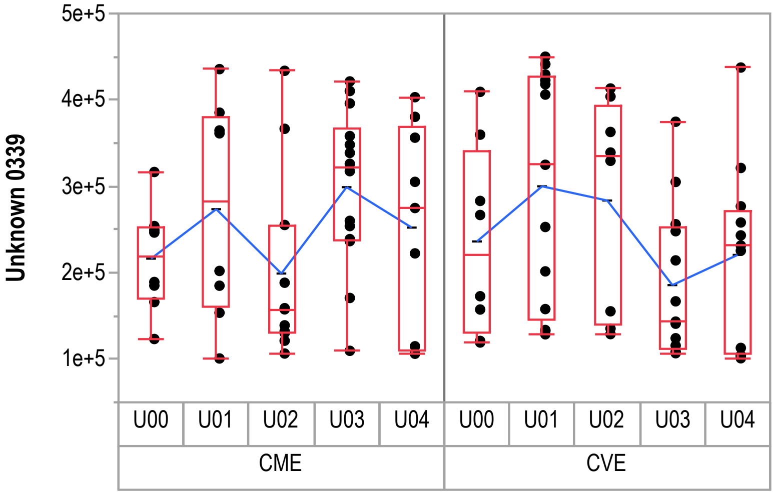

Variability Gauge

Variability Chart for Unknown 0430*

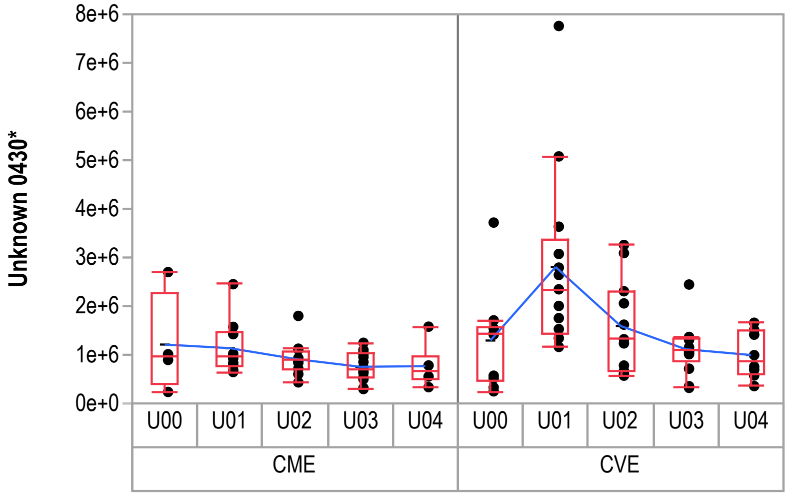

Variability Gauge

Variability Chart for Unknown 0456

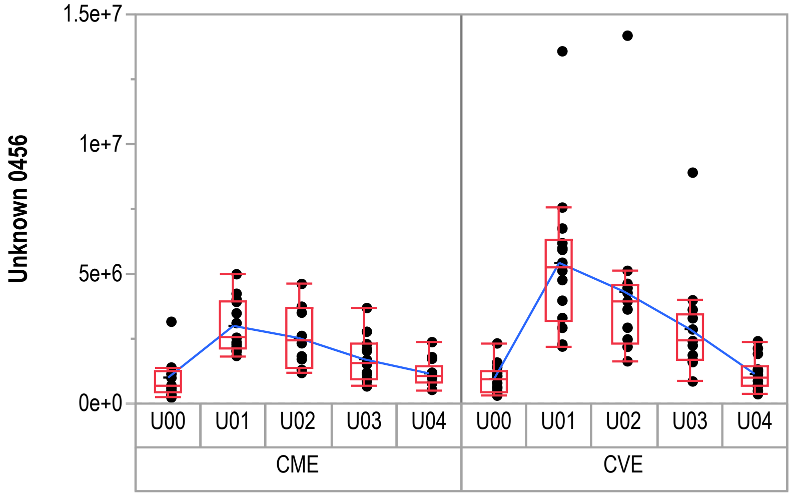

Variability Gauge

Variability Chart for Unknown 0468

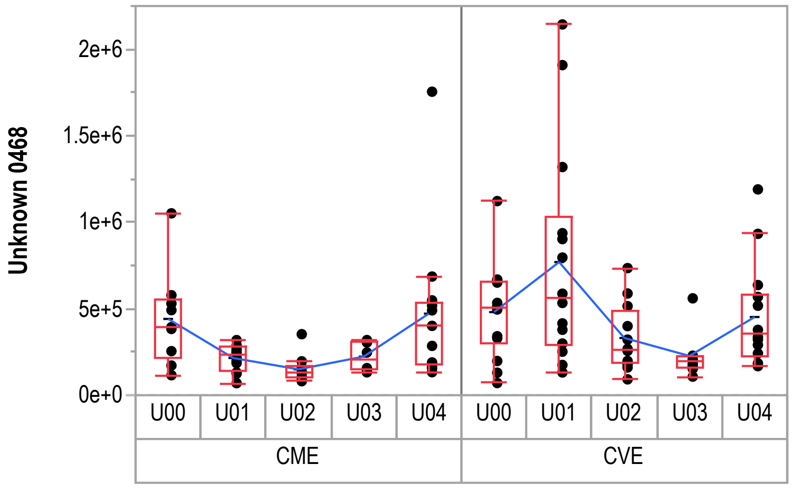

Variability Gauge

Variability Chart for Unknown 0529

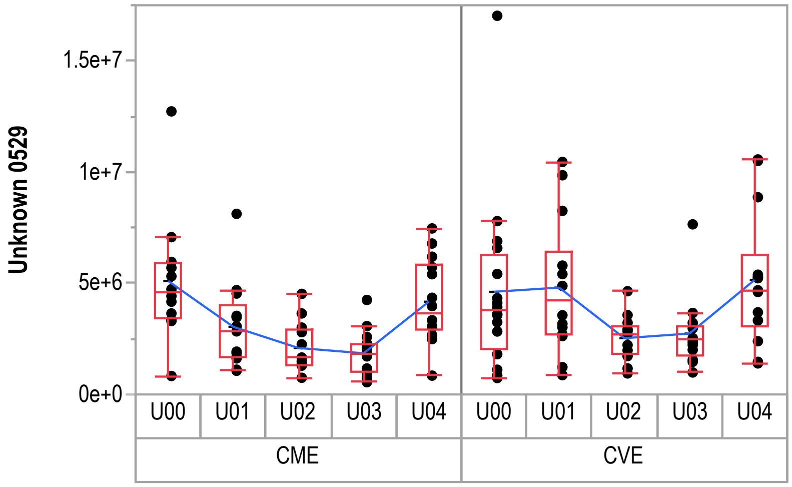

Variability Gauge

Variability Chart for Unknown 0533

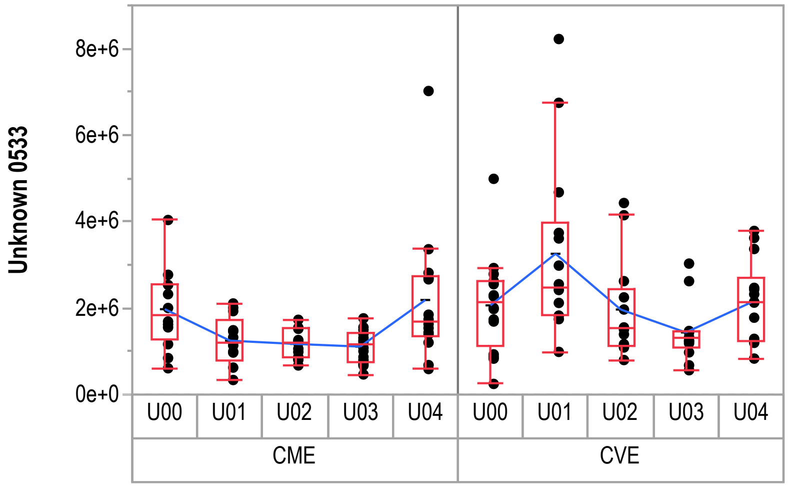

Variability Gauge

Variability Chart for Unknown 0569

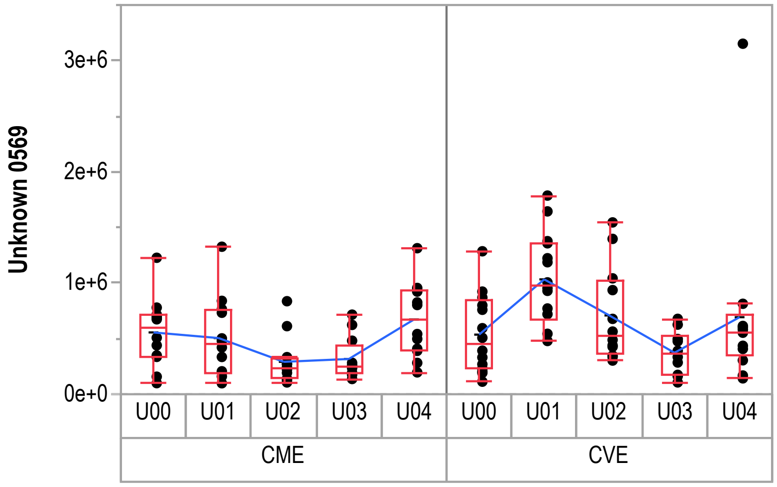

Variability Gauge

Variability Chart for Unknown 0598

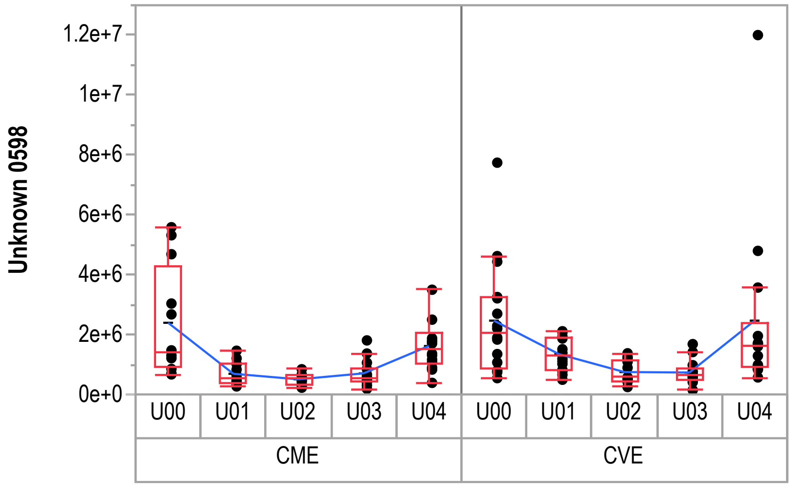

Variability Gauge

Variability Chart for Unknown 0603

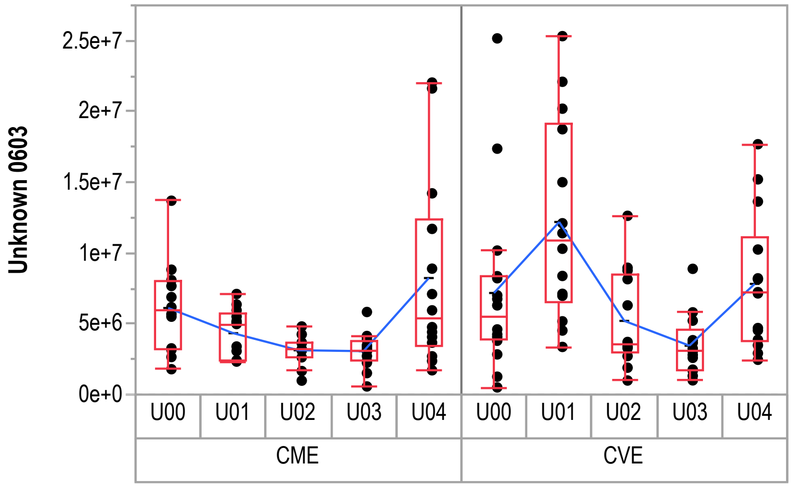

Variability Gauge

Variability Chart for Unknown 0629

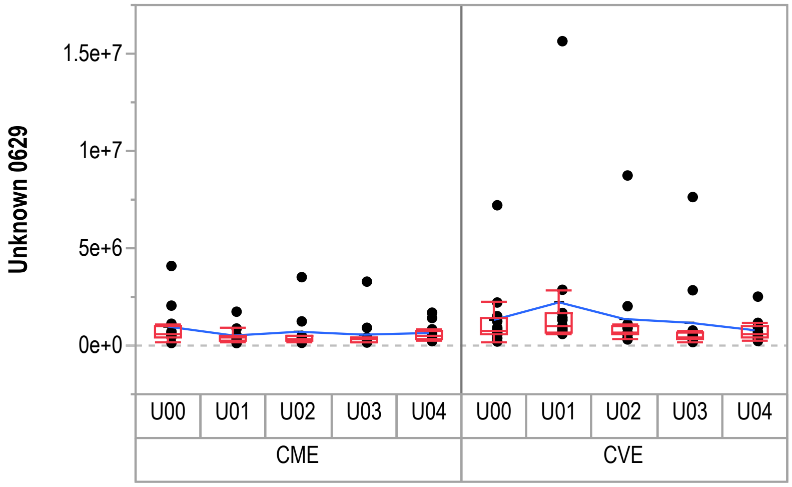

Variability Gauge

Variability Chart for Unknown 0673

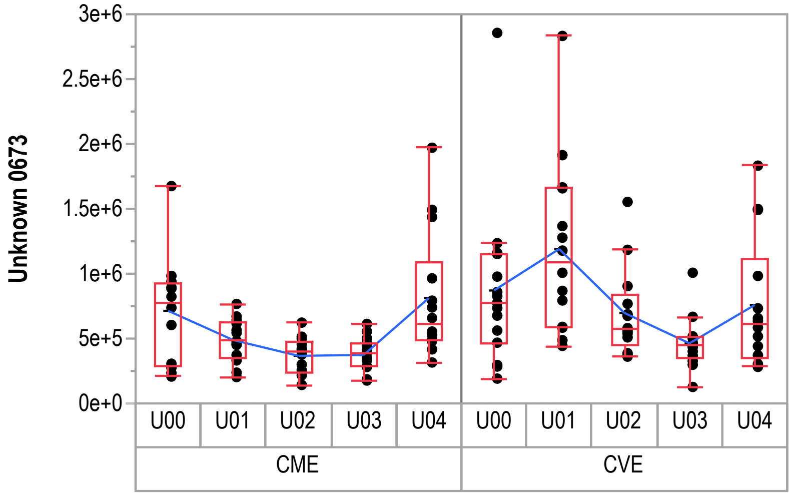

Variability Gauge

Variability Chart for Unknown 0688

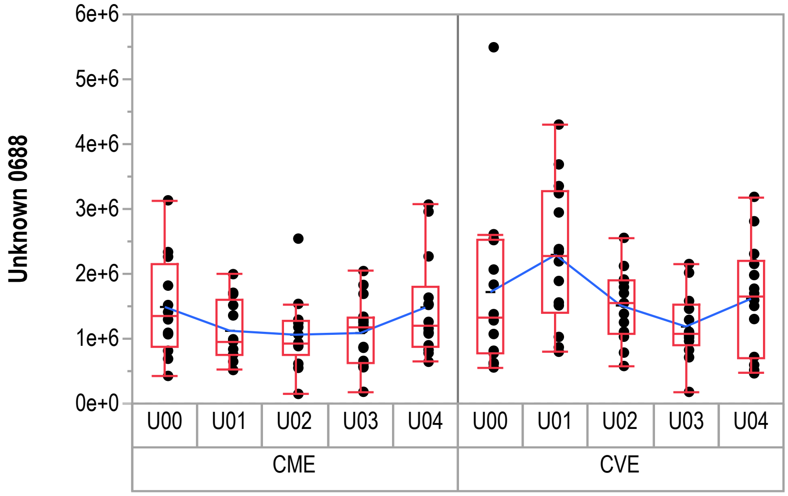

Variability Gauge

Variability Chart for Unknown 0737

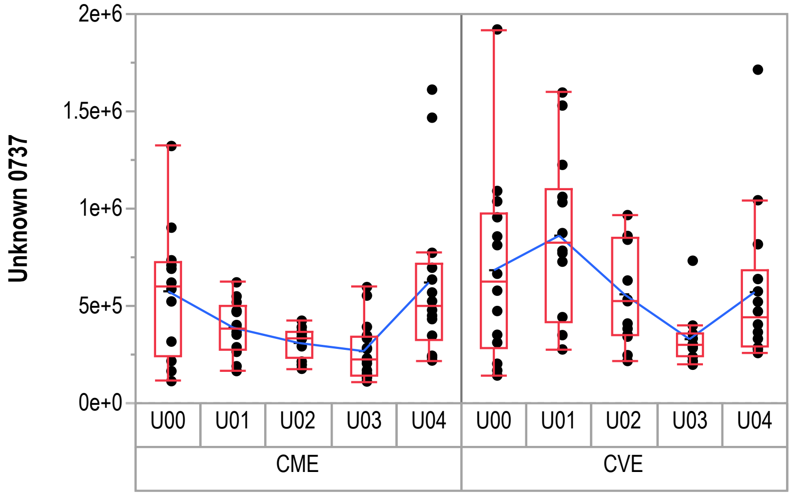

Variability Gauge

Variability Chart for Unknown 0740

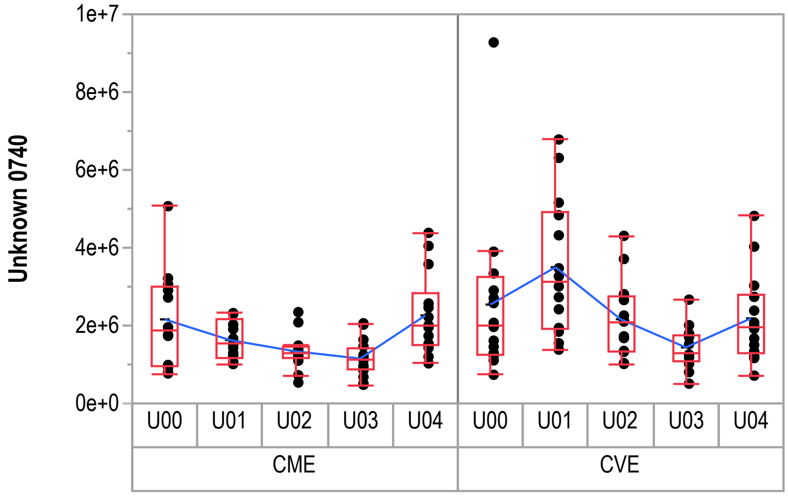

Variability Gauge

Variability Chart for Unknown 0775

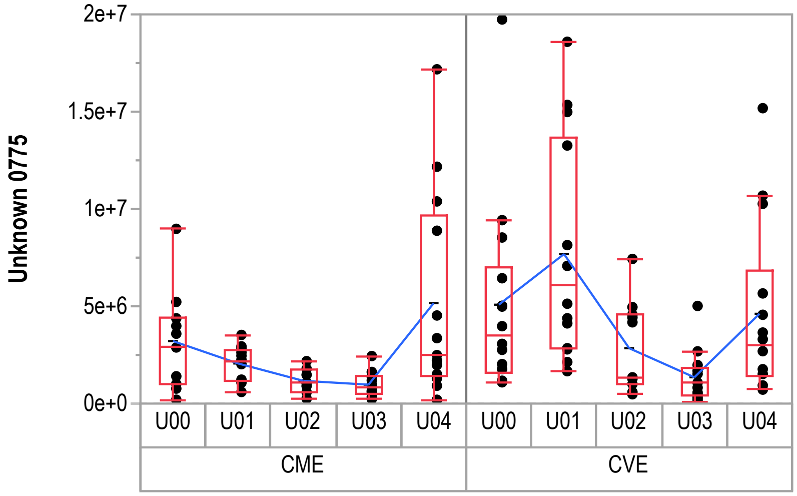

Variability Gauge

Variability Chart for Unknown 0794

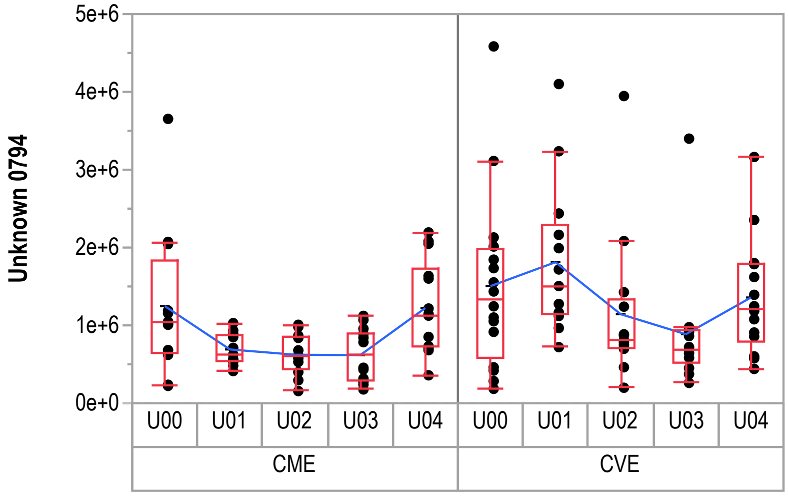

Variability Gauge

Variability Chart for Unknown 0829

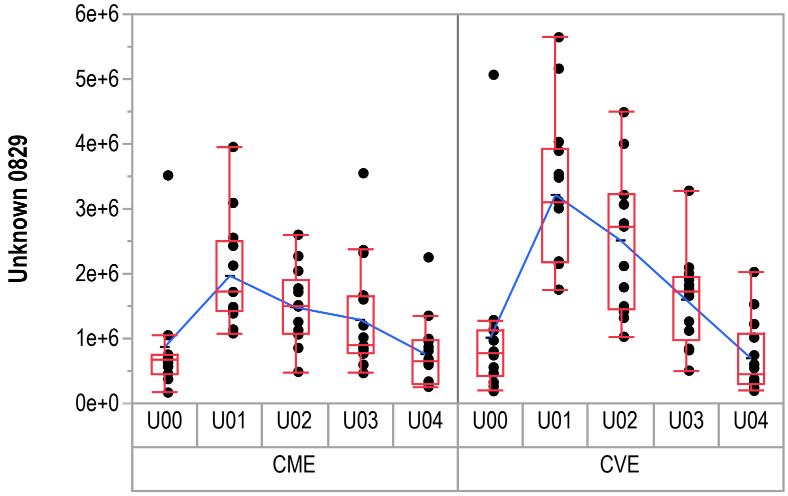

Variability Gauge

Variability Chart for Unknown 0876

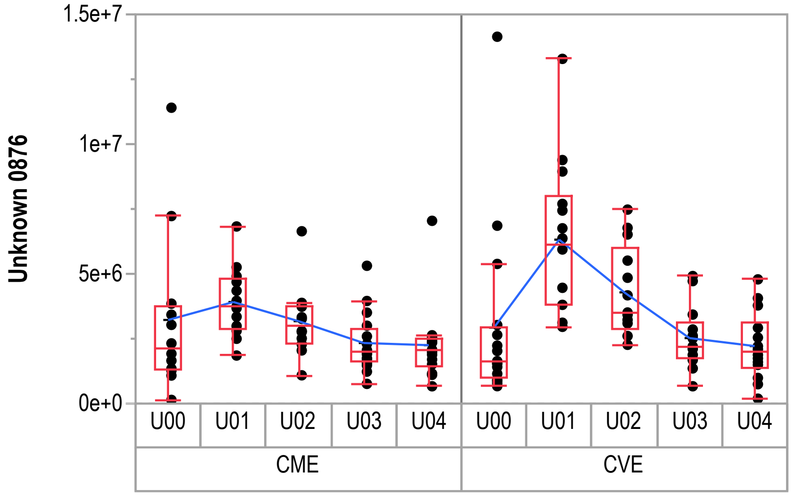

Variability Gauge

Variability Chart for Unknown 0928*

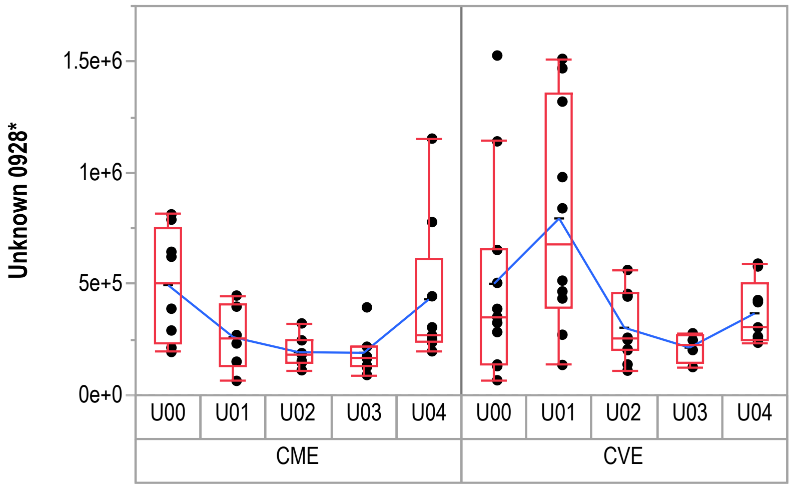

Variability Gauge

Variability Chart for Unknown 0956

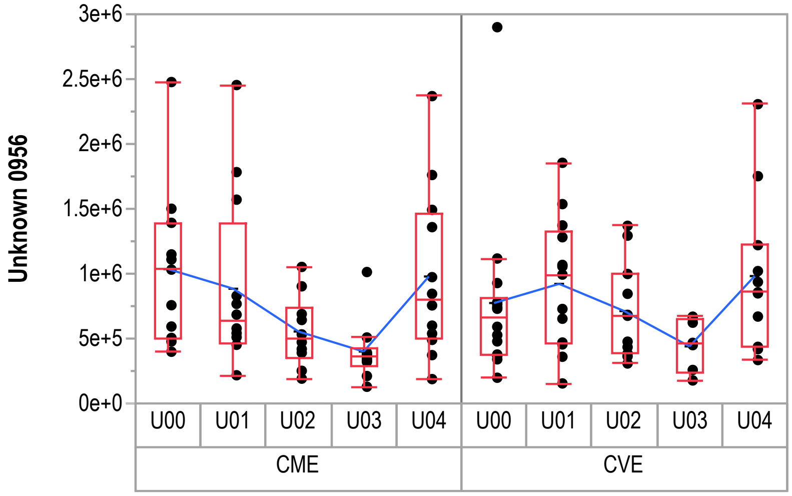

Variability Gauge

Variability Chart for Unknown 1069

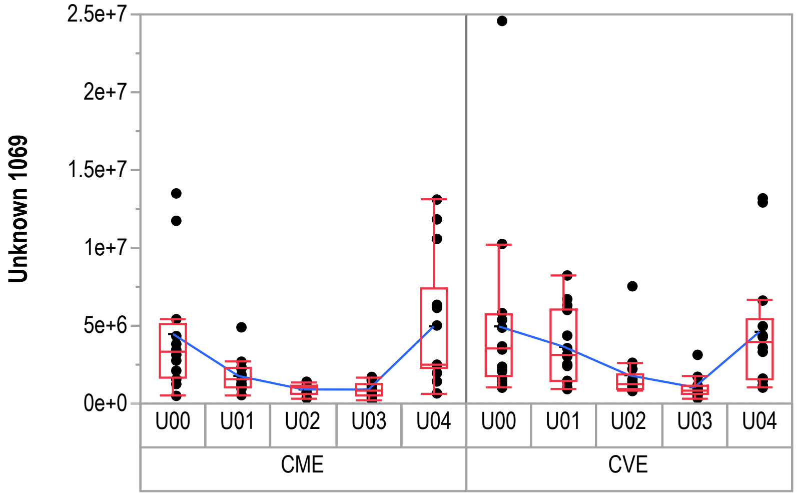

Variability Gauge

Variability Chart for Unknown 1083

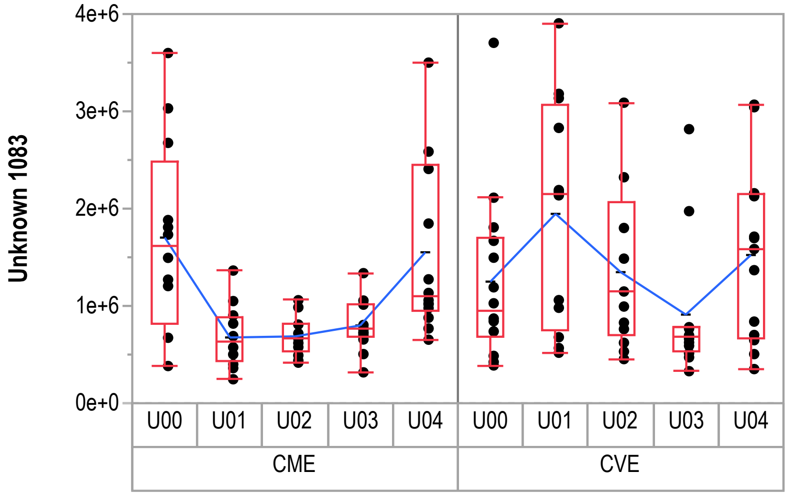

Variability Gauge

Variability Chart for Unknown 1091

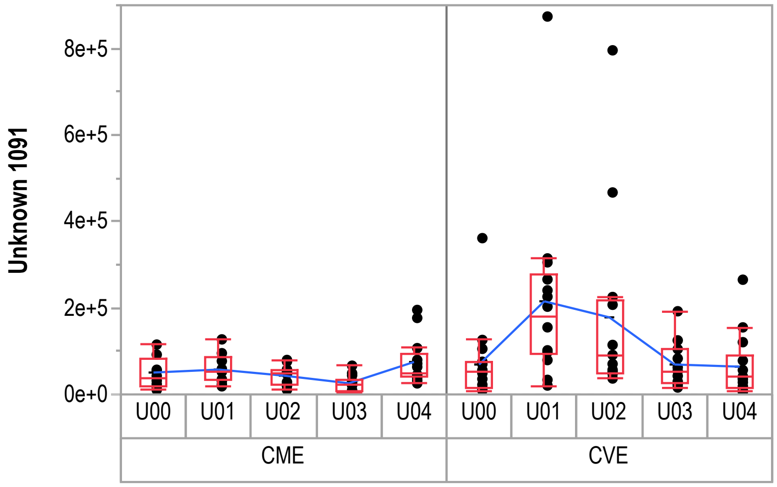

Variability Gauge

Variability Chart for Unknown 1163

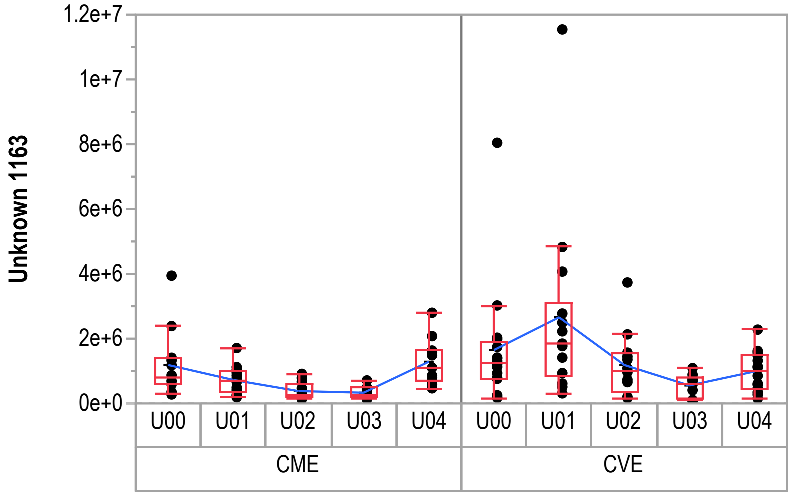

Variability Gauge

Variability Chart for Uracil

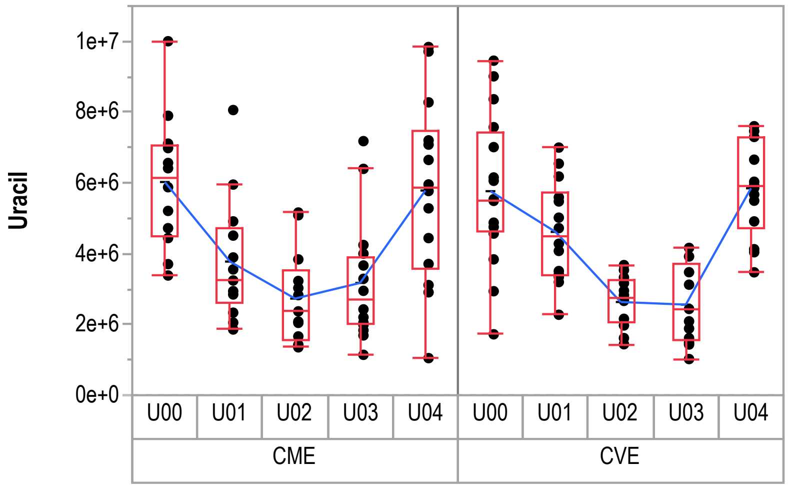

Variability Gauge

Variability Chart for Xanthine

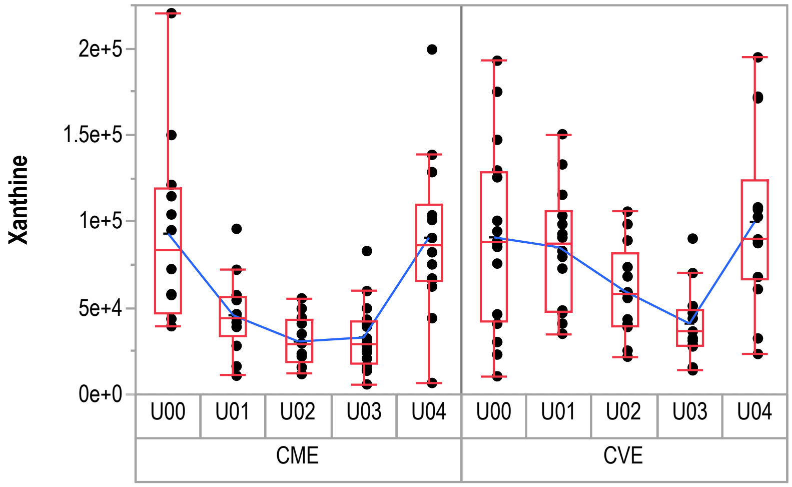

Variability Gauge

Variability Chart for γ-Butyrobetaine

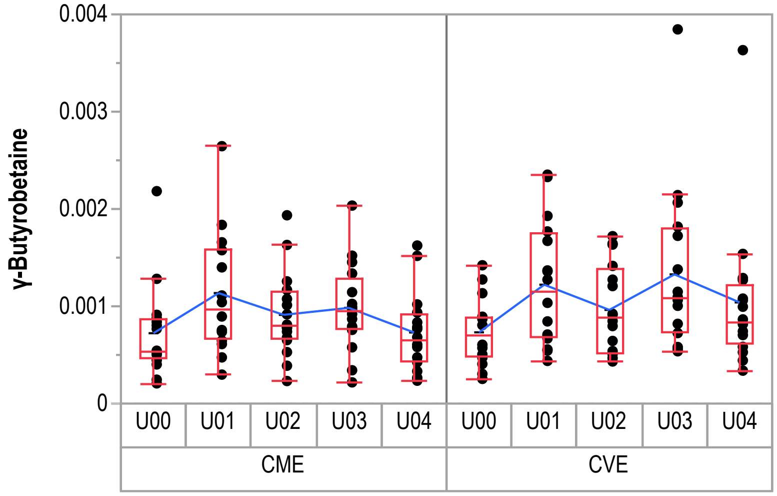

Supplement: Supplementary file 1 [file DataSheet2.PDF]
