# Supplementary figures and images for: Acute effects of moderate vs. vigorous endurance exercise on urinary metabolites in healthy, young, physically active men—A multi-platform metabolomics approach
Source: Front Physiol. 2023 Jan 30;14:1028643. doi: 10.3389/fphys.2023.1028643 (PMC9927024; doi:10.3389/fphys.2023.1028643)

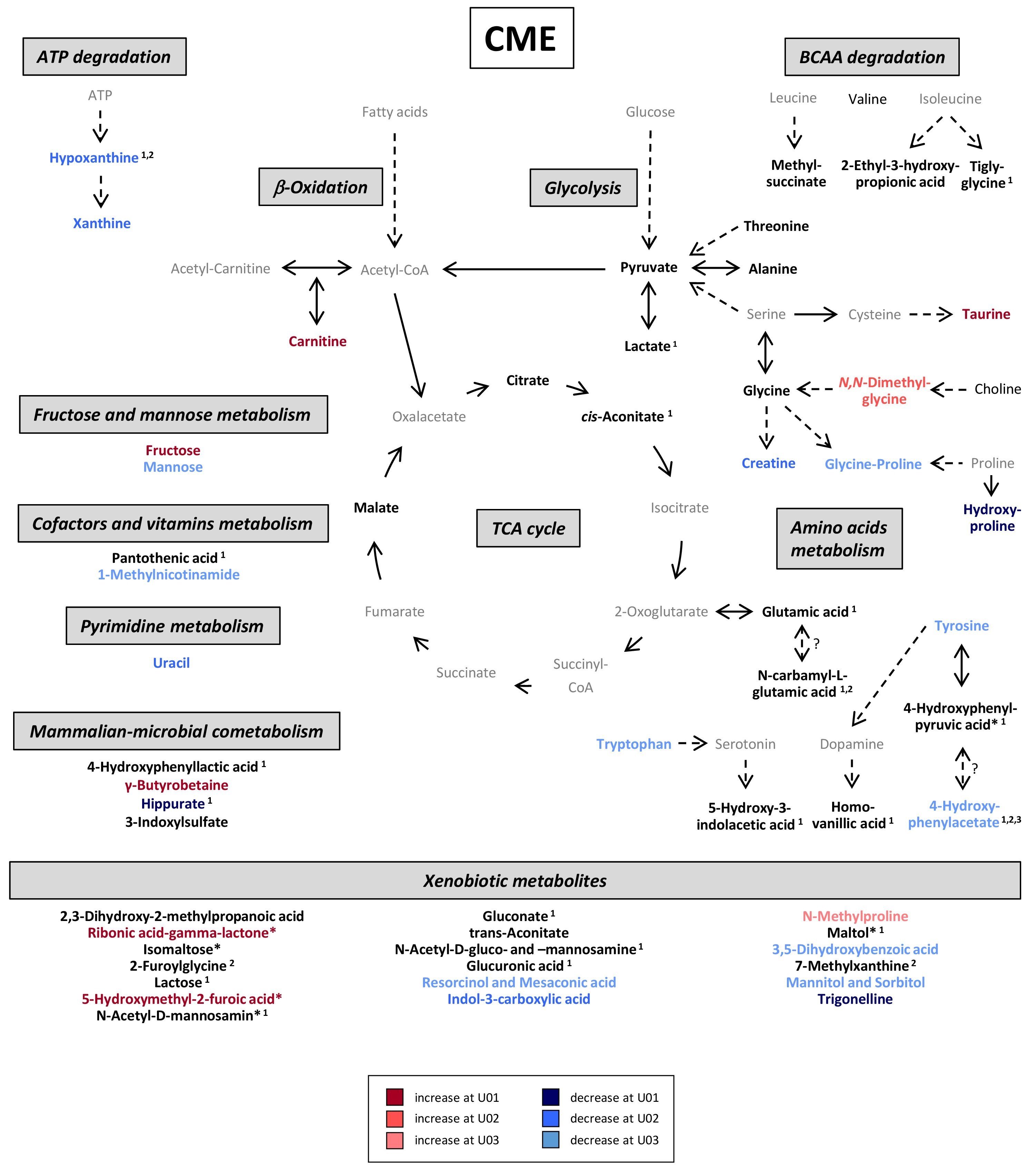

Supplement: Supplementary file 2 [file Image3.JPEG]

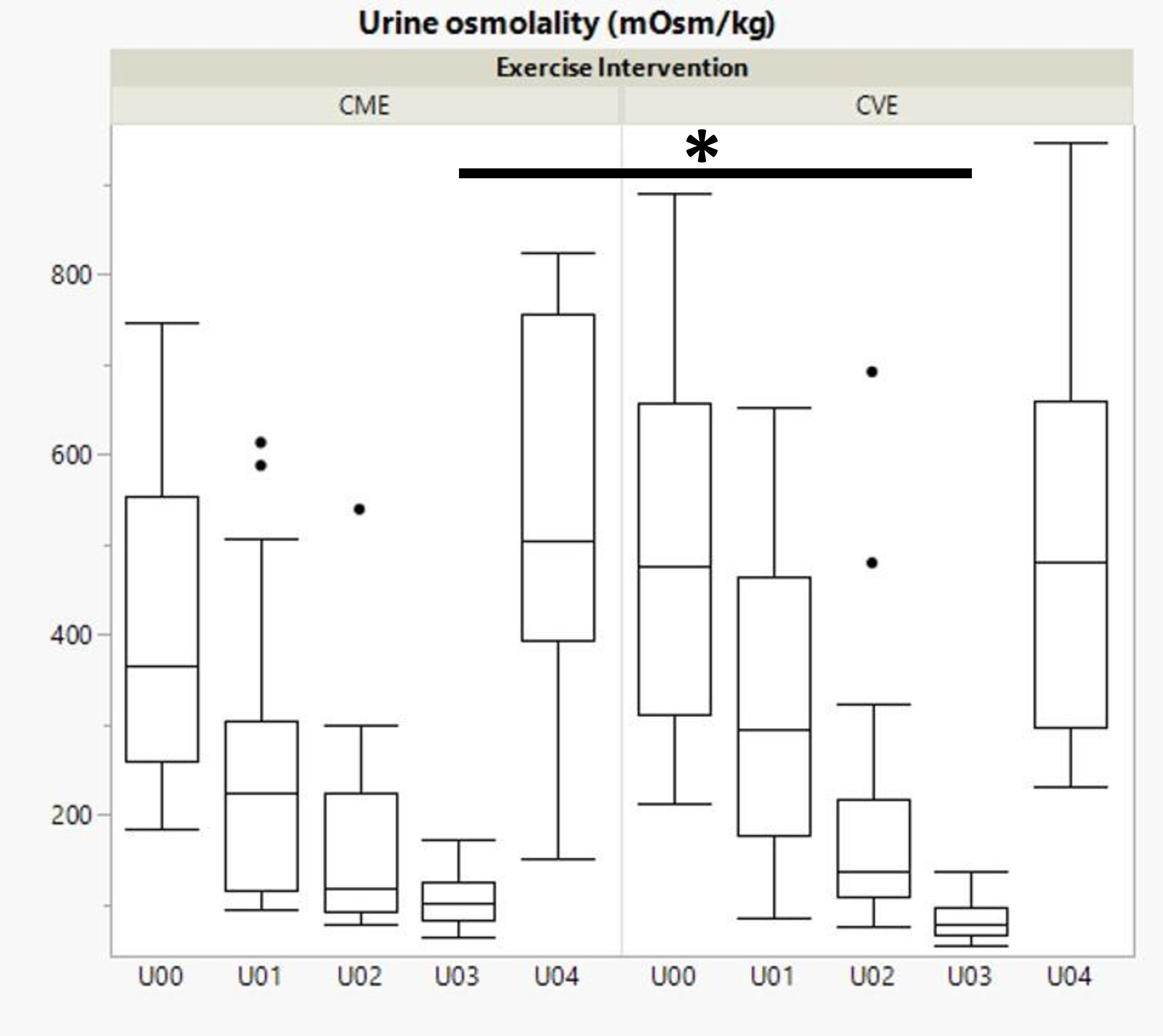

Supplement: Supplementary file 5 [file Image1.JPEG]

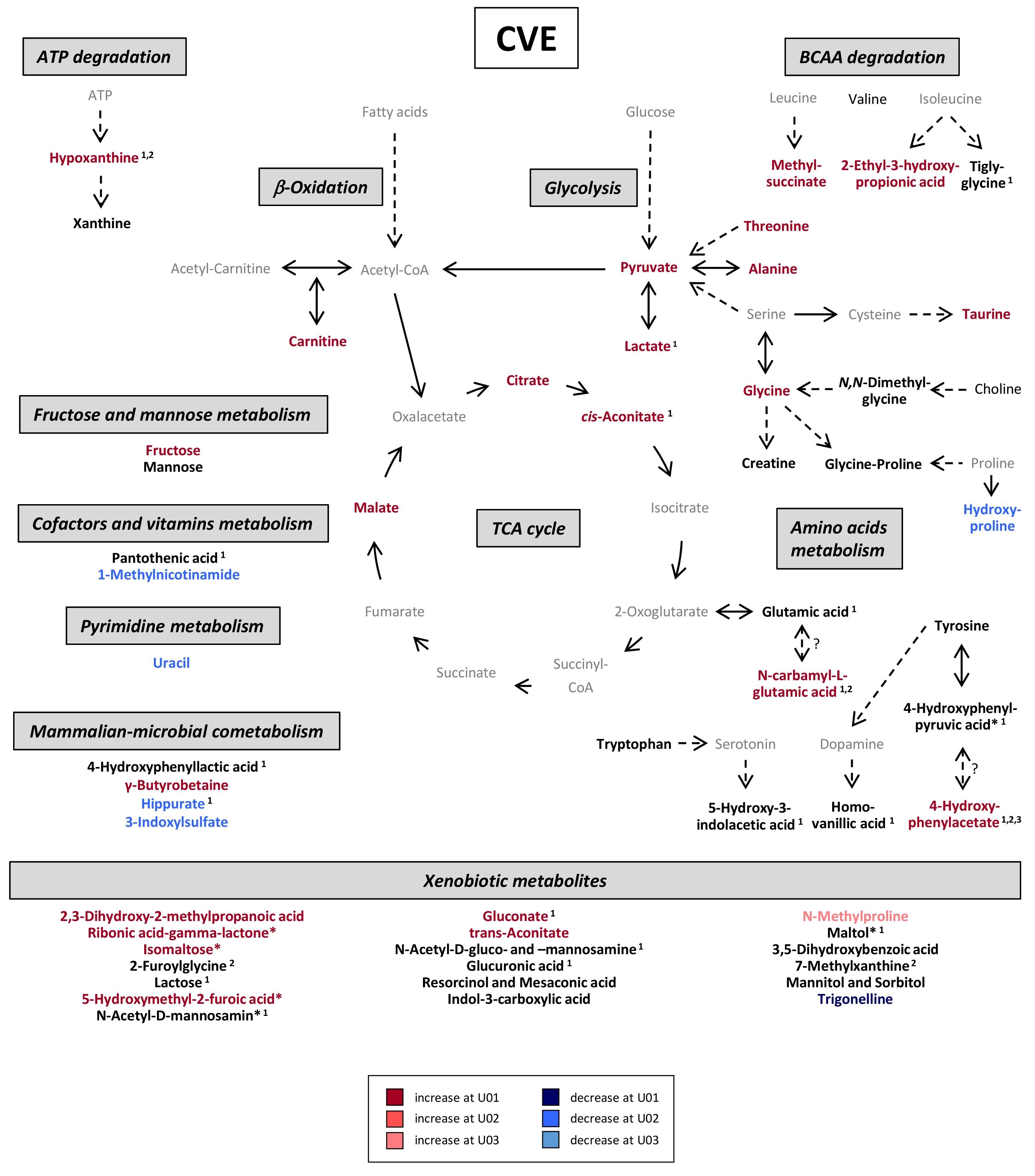

Supplement: Supplementary file 6 [file Image4.JPEG]

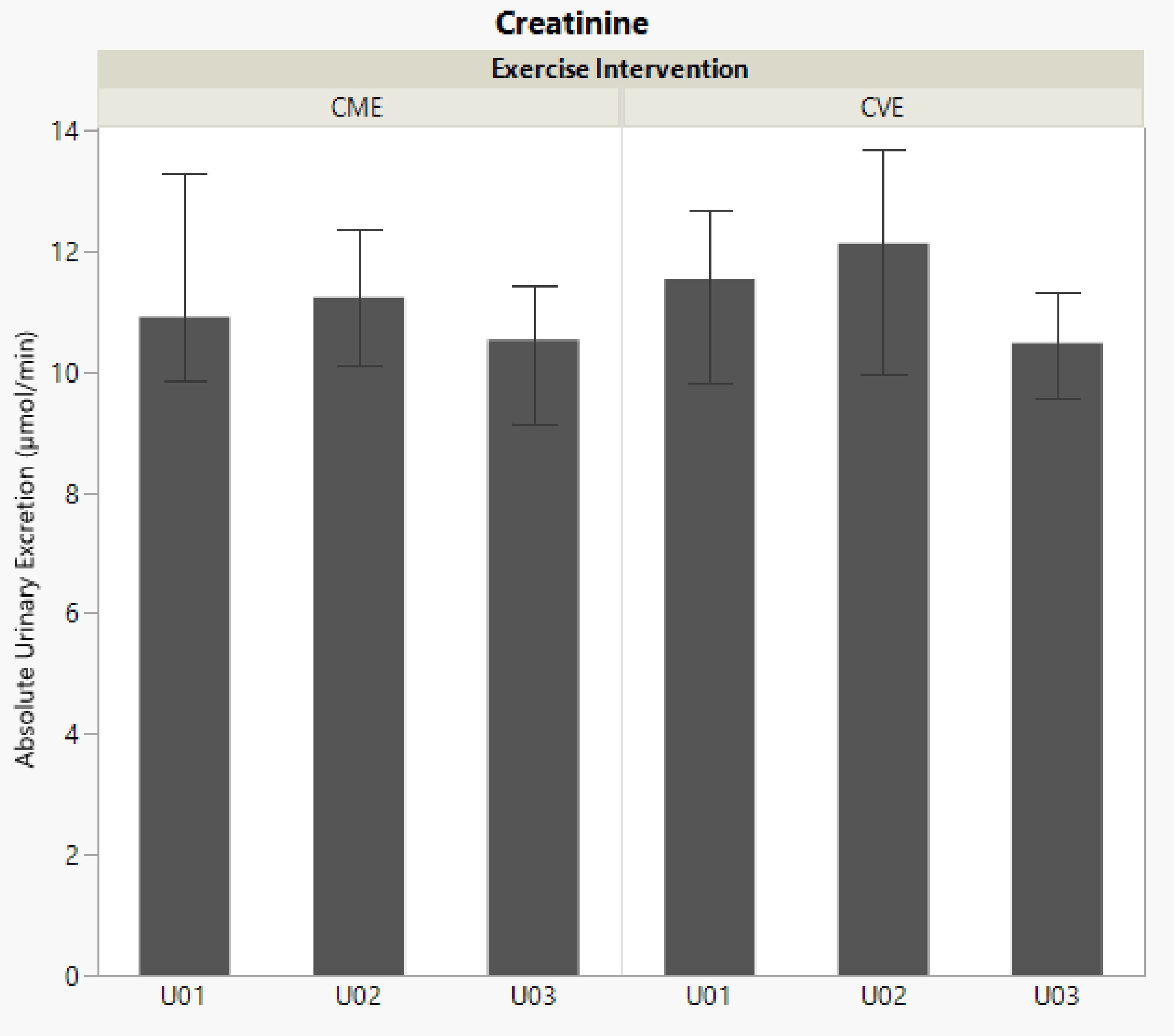

Supplement: Supplementary file 7 [file Image7.JPEG]

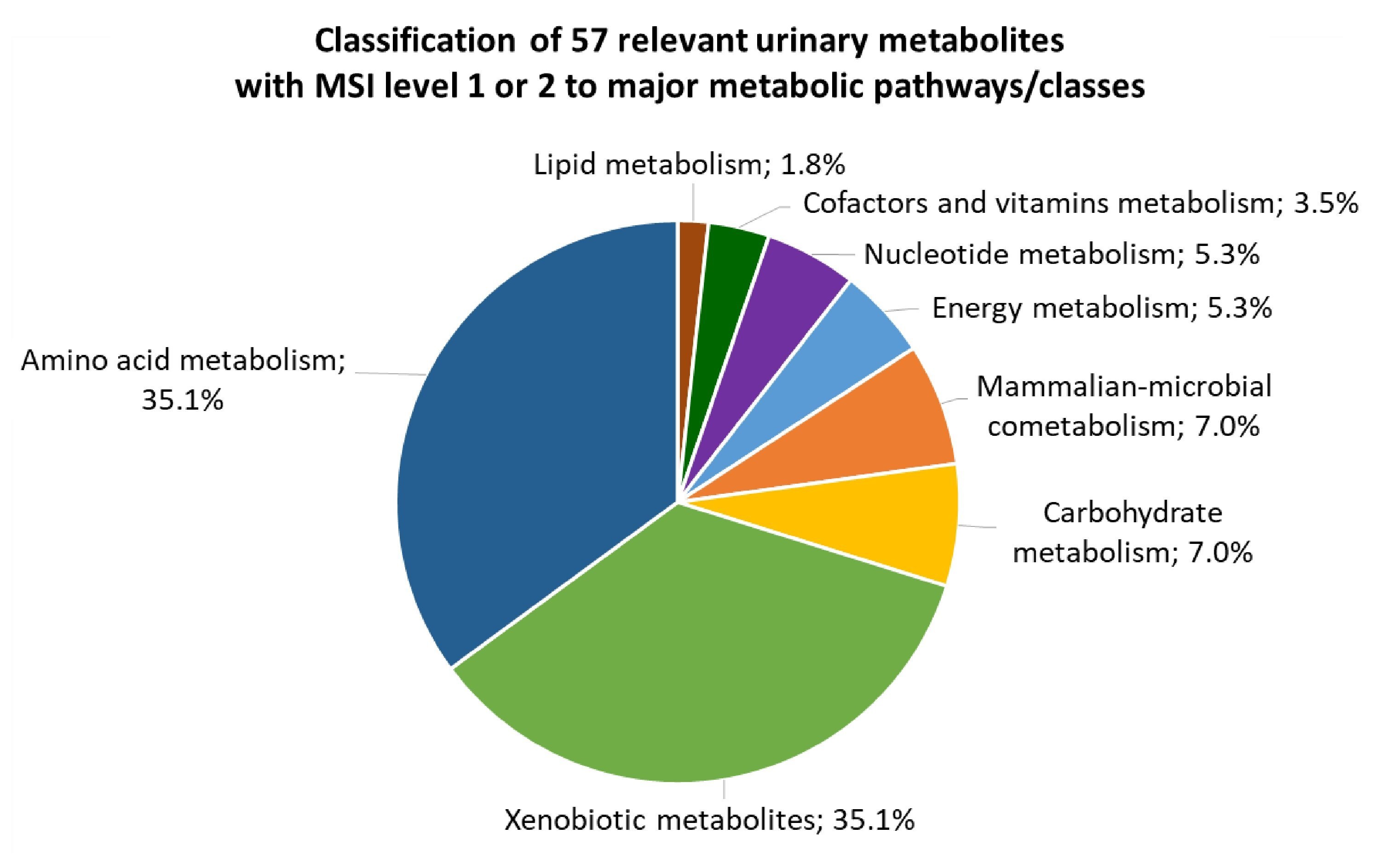

Supplement: Supplementary file 8 [file Image2.JPEG]

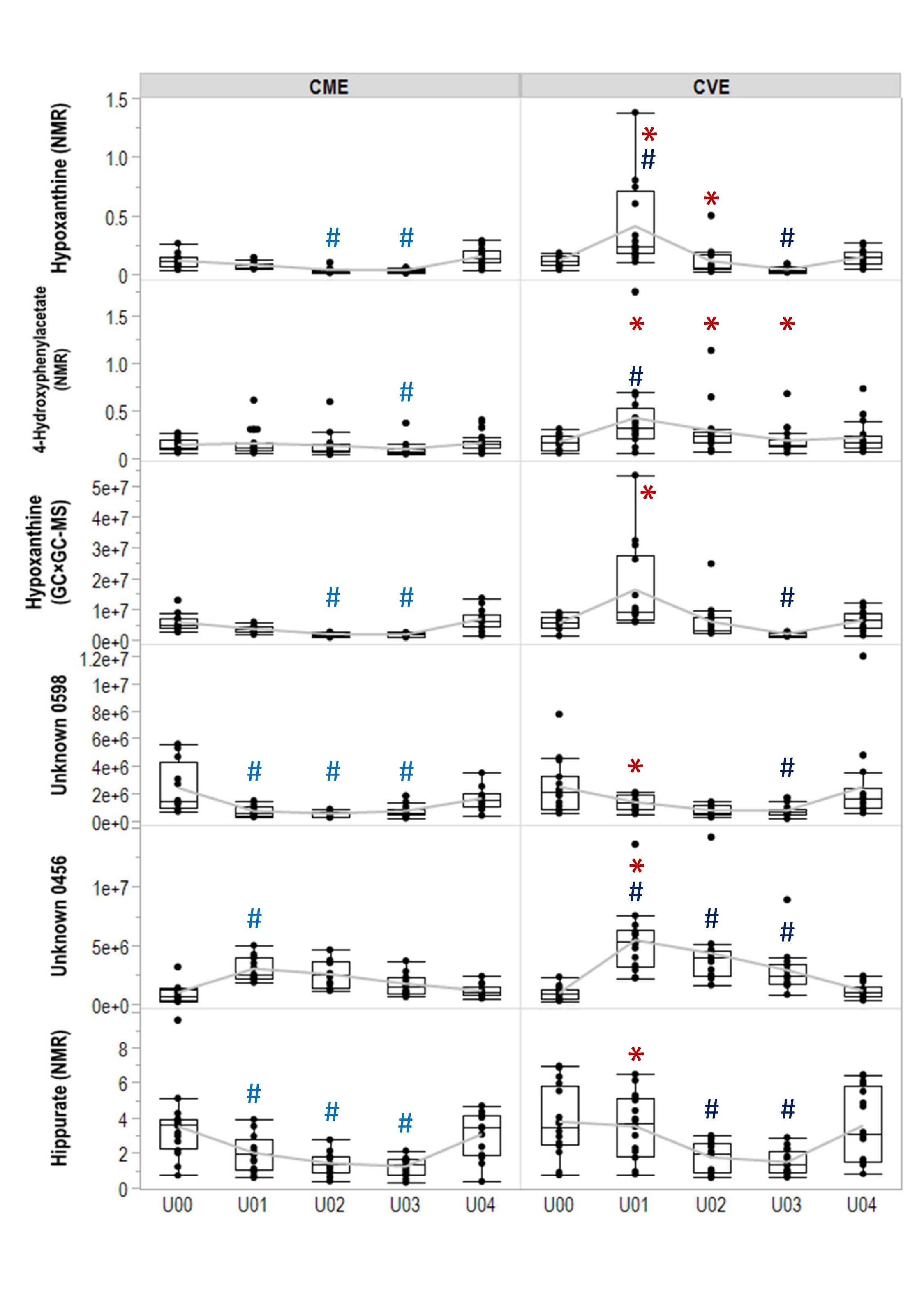

Supplement: Supplementary file 9 [file Image5.JPEG]

# Absolute excretion of metabolites with relevant median FCs for CVE/CME ratio

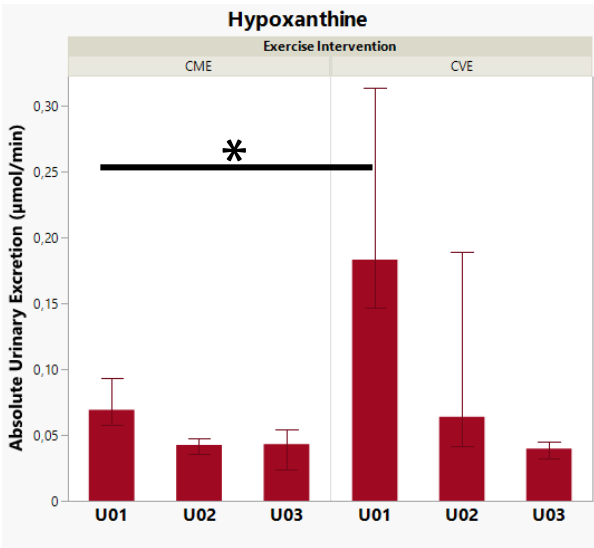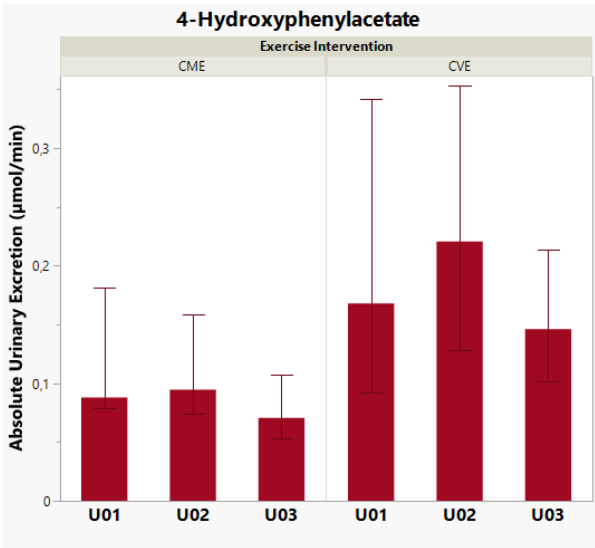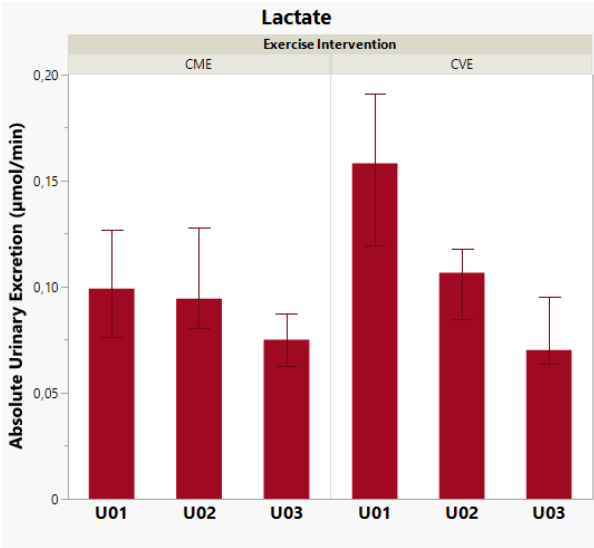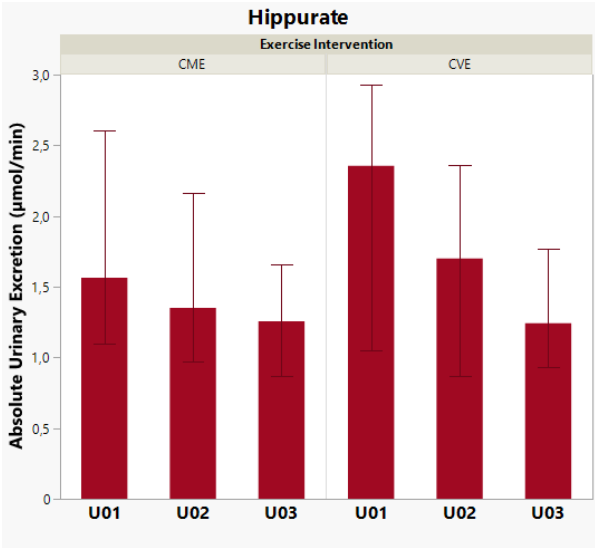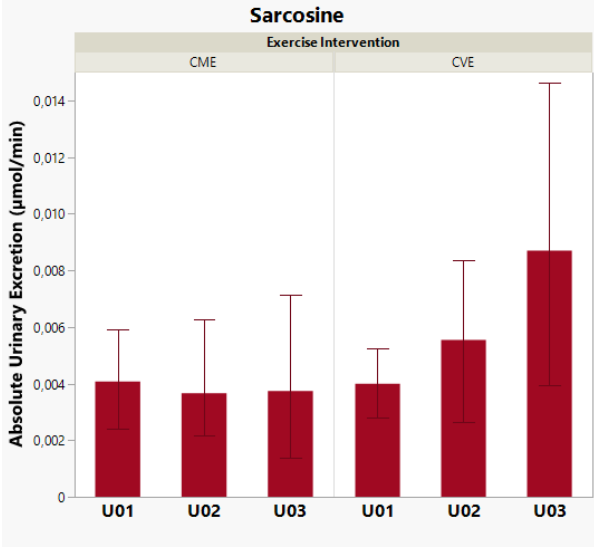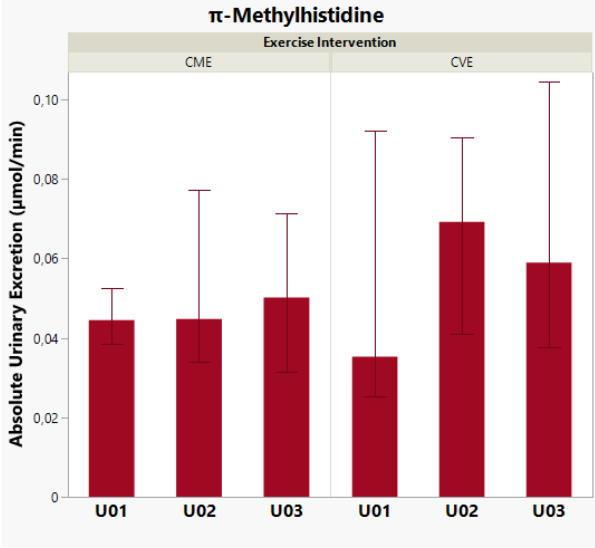

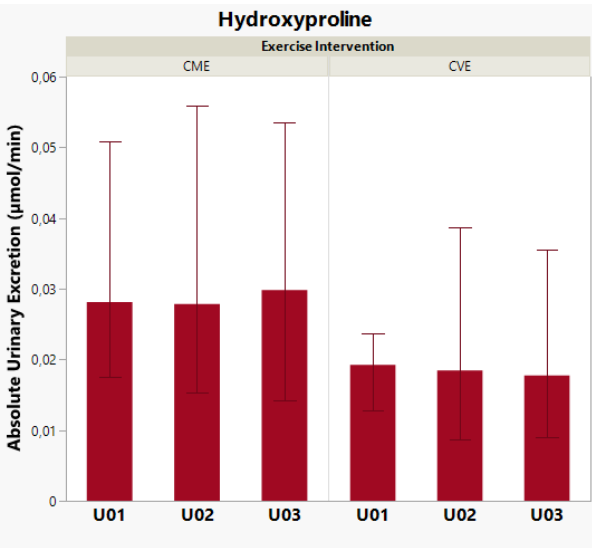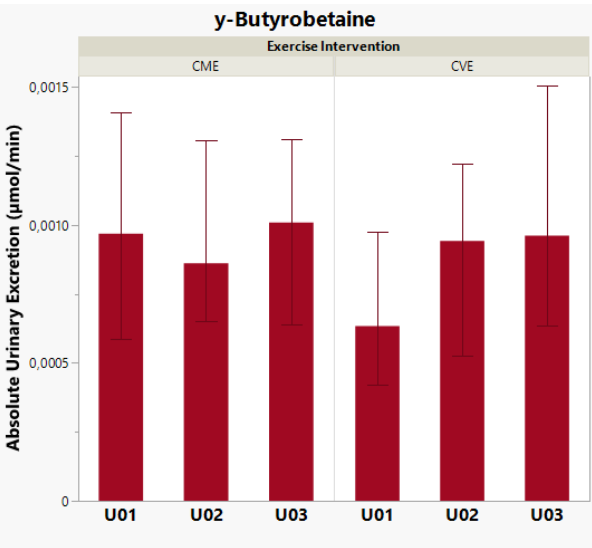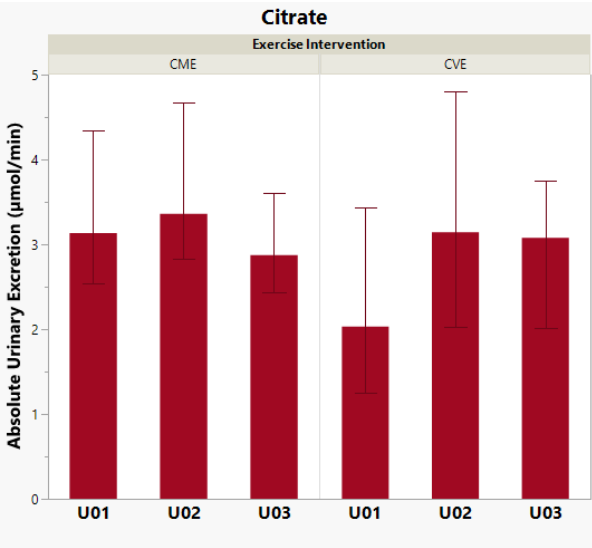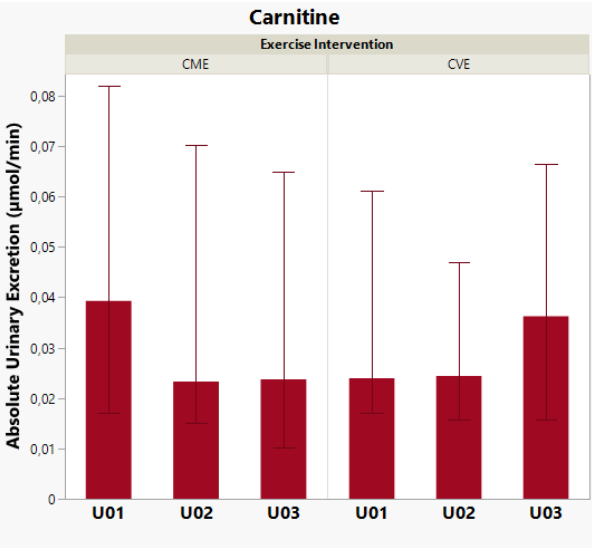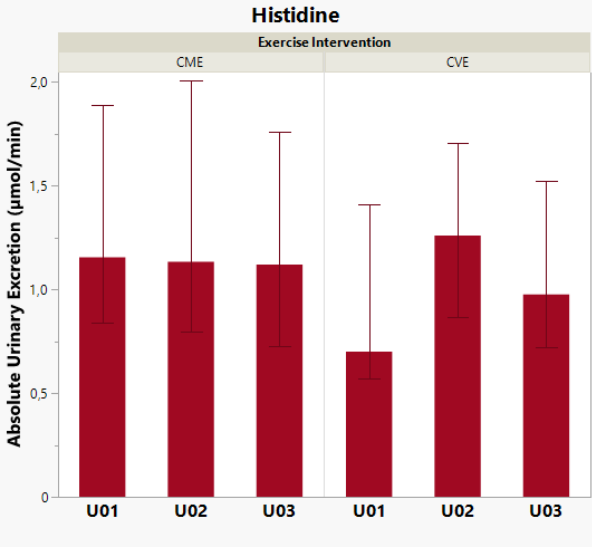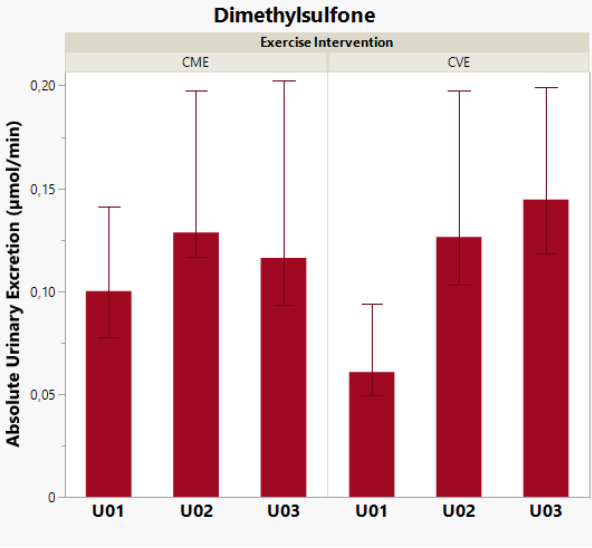

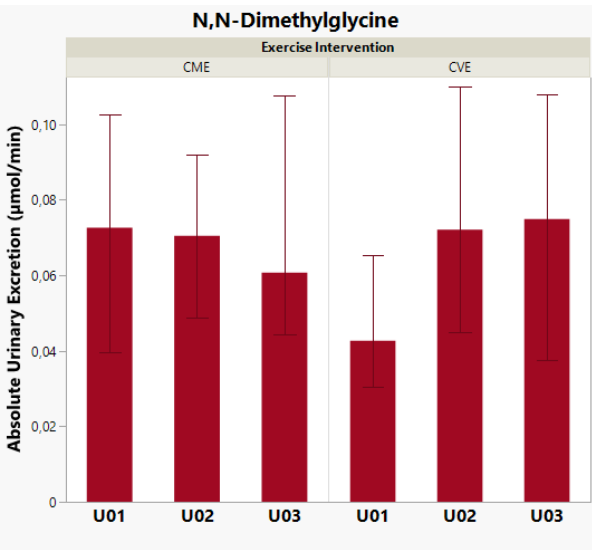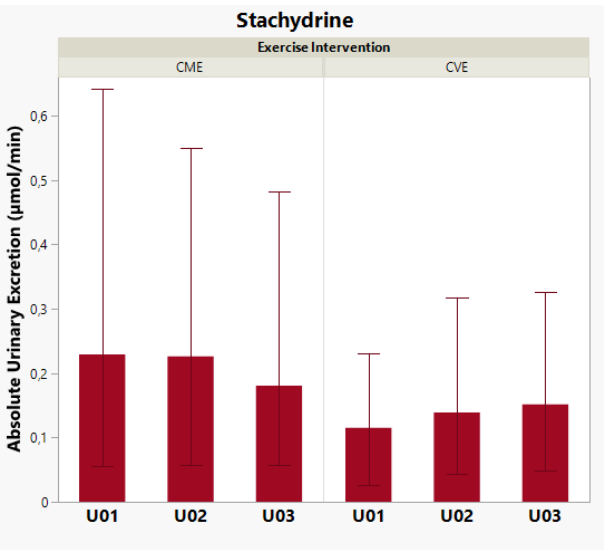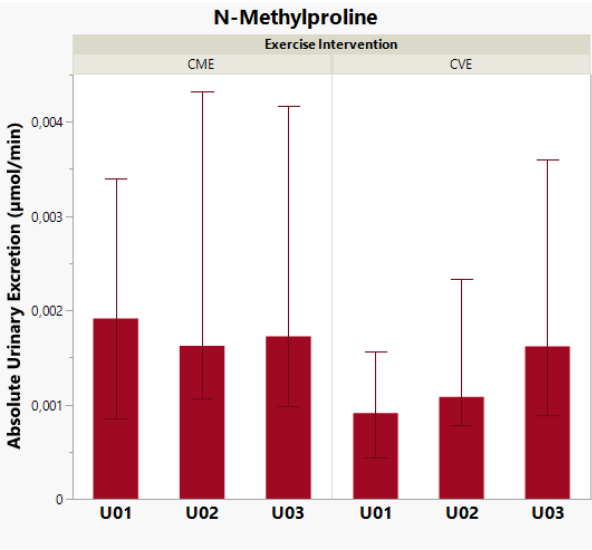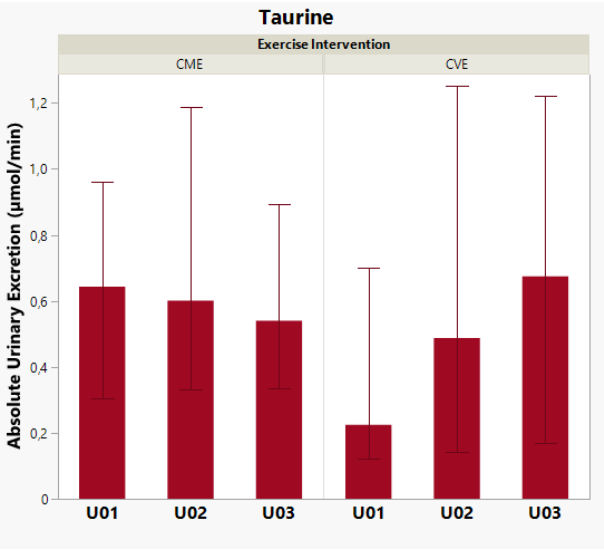

Supplement: Supplementary file 10 [file DataSheet3.PDF]

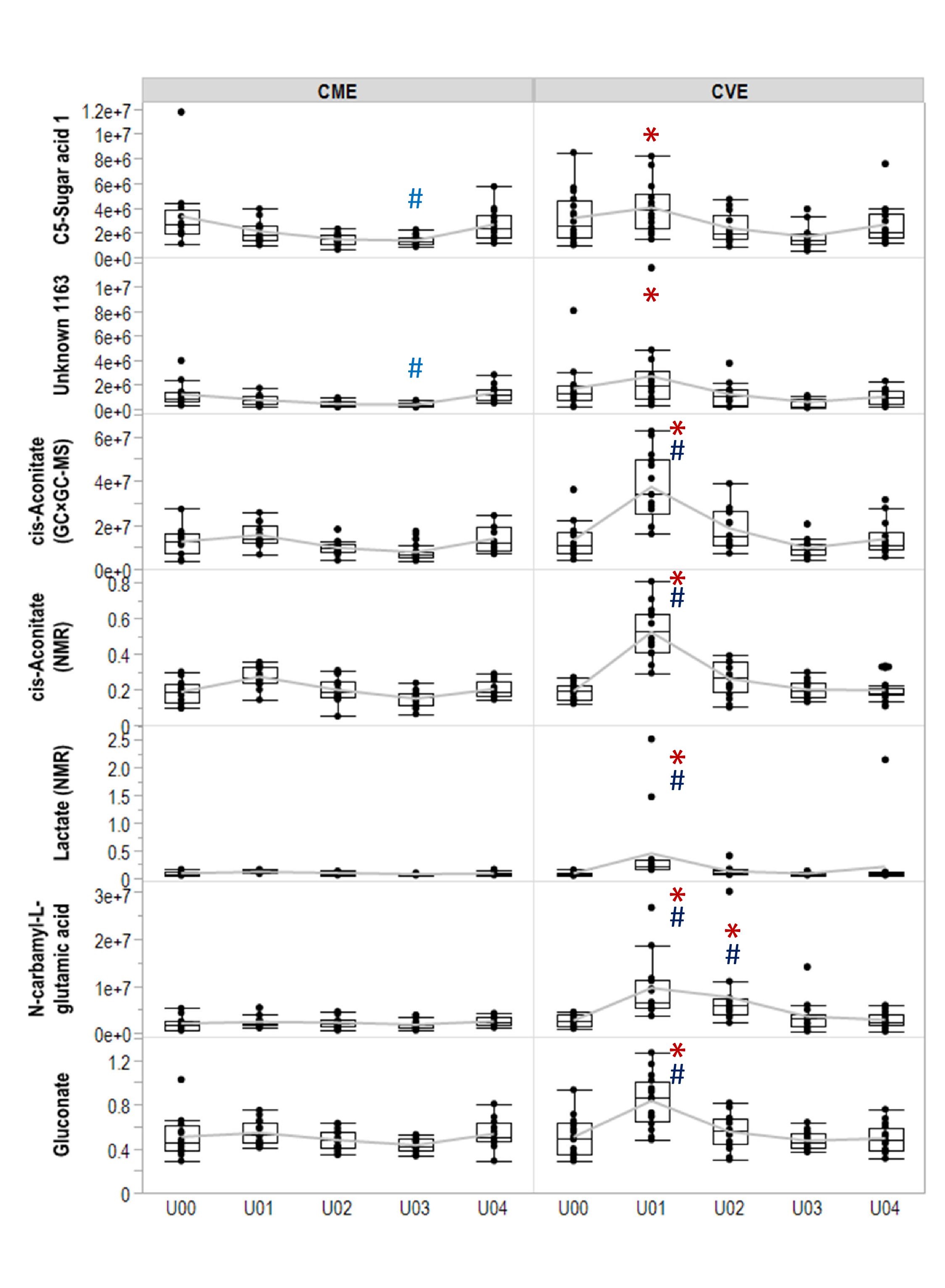

Supplement: Supplementary file 16 [file Image6.JPEG]
