# Supplementary material for: Acute effects of moderate vs. vigorous endurance exercise on urinary metabolites in healthy, young, physically active men—A multi-platform metabolomics approach
Source: Front Physiol. 2023 Jan 30;14:1028643. doi: 10.3389/fphys.2023.1028643 (PMC9927024; doi:10.3389/fphys.2023.1028643)
Supplement: Supplementary file 4 [file Table1.DOCX]

| Inclusion Criteria | Exclusion Criteria |
| --- | --- |
| - Healthy, physically active men - Minimum age of 18 years - Non-smokers - Volunteers giving their written and informed consent | - Smokers - Volunteers with diseases of the gastrointestinal tract, metabolism, nervous system and infectious or immunological diseases in therapeutic need - Volunteers with tumors, acute or chronic infectious diseases - Volunteers with drug or alcohol abuse - Volunteers with diseases of the cardiovascular system and/or cardiac pacemaker - Volunteers with intolerances against gluten, fructose or lactose - Volunteers who donated blood in the last three months - Volunteers who may not adhere to the study protocol - Institutionalized patients in psychiatric hospitals |
