# Supplementary material for: Acute effects of moderate vs. vigorous endurance exercise on urinary metabolites in healthy, young, physically active men—A multi-platform metabolomics approach
Source: Front Physiol. 2023 Jan 30;14:1028643. doi: 10.3389/fphys.2023.1028643 (PMC9927024; doi:10.3389/fphys.2023.1028643)
Supplement: Supplementary file 11 [file Table2.DOCX]

| ***Diet (pre-IV day and IV day)*** | ***Mean*** | **±** | ***SD*** | ***Min.*** | | ***Max.*** | |
| --- | --- | --- | --- | --- | --- | --- | --- |
| Energy intake (kcal)^1^ | 4095 | ± | 170 | 3770 | | 4324 | |
| Carbohydrates (%)^1^ | 55.0 | ± | 1.6 | 52.5 | | 57.4 | |
| Carbohydrates (g) ^1^ | 555 | ± | 24 | 523 | | 593 | |
| Fat (%)^1^ | 31.7 | ± | 1.7 | 29.1 | | 34.0 | |
| Fat (g)^1^ | 145 | ± | 10 | 125 | | 163 | |
| Proteins (%)^1^ | 10.9 | ± | 0.3 | 10.5 | | 11.4 | |
| Proteins (g)^1^ | 110 | ± | 7 | 97 | | 121 | |
| Fibers (%)^1^ | 2.3 | ± | 0.1 | 2.2 | | 2.4 | |
| Fibers (g)^1^ | 48.8 | ± | 2.5 | 44.9 | | 52.1 | |
| ***Breakfast (pre-CME trial)*** |  | | |  |  | |  |
| Energy intake (kcal)^2^ | 877 | ± | 207 | 456 | | 1428 | |
| Carbohydrates (%)^2^ | 63.4 | ± | 6.1^#^ | 53.6 | | 77.6 | |
| Carbohydrates (g)^2^ | 137 | ± | 29 | 72 | | 197 | |
| Fat (%)^2^ | 25.5 | ± | 6.1 | 11.5 | | 35.9 | |
| Fat (g)^2^ | 25.7 | ± | 10.9 | 10.7 | | 57.7 | |
| Proteins (%)^2^ | 9.9 | ± | 2.6^°^ | 6.9 | | 15.9 | |
| Proteins (g)^2^ | 21.4 | ± | 7.1^*^ | 13.1 | | 37.1 | |
| Fibers (%)^2^ | 1.1 | ± | 0.1 | 0.9 | | 1.4 | |
| Fibers (g)^2^ | 5.2 | ± | 1.2 | 3.0 | | 7.9 | |
| ***Breakfast (pre-CVE trial)*** |  | | |  |  | | |
| Energy intake (kcal)^2^ | 994 | ± | 274 | 549 | | 1455 | |
| Carbohydrates (%)^2^ | 58.2 | ± | 6.8^#^ | 44.4 | | 69.7 | |
| Carbohydrates (g)^2^ | 142 | ± | 38 | 91 | | 226 | |
| Fat (%)^2^ | 29.2 | ± | 5.5 | 18.4 | | 38.3 | |
| Fat (g)^2^ | 33.2 | ± | 12.2 | 11.3 | | 53.1 | |
| Proteins (%)^2^ | 11.6 | ± | 2.5^°^ | 7.4 | | 16.3 | |
| Proteins (g)^2^ | 28.8 | ± | 11.3^*^ | 13.0 | | 53.5 | |
| Fibers (%)^2^ | 1.1 | ± | 0.1 | 0.9 | | 1.4 | |
| Fibers (g)^2^ | 5.5 | ± | 1.5 | 4.0 | | 8.2 | |
| ***Liquid intake (post-exercise)*** |  |  |  |  | |  | |
| Water intake after CME trial (L) | 2.2 | ± | 0.6^◊^ | 1.3 | | 3.1 | |
| Water intake after CVE trial (L) | 2.7 | ± | 0.8^◊^ | 1.3 | | 4.0 | |

^1^: calculated energy/nutrient intake, i.e., amounts that should be consumed on pre-intervention and intervention days; ^2^: actual energy nutrient intake, i.e., amounts that were actually consumed at breakfast on intervention days, */#/°/◊: significant difference (p*<*0.05) between CME and CVE trial based on paired Wilcoxon signed-rank test. %: percentage of energy intake from carbohydrates, fat, proteins, or fibers. CME: continuous moderate exercise; CVE: continuous vigorous exercise; IV: intervention; Max: maximum; Min: minimum; SD: standard deviation.
