# Supplementary material for: Acute effects of moderate vs. vigorous endurance exercise on urinary metabolites in healthy, young, physically active men—A multi-platform metabolomics approach
Source: Front Physiol. 2023 Jan 30;14:1028643. doi: 10.3389/fphys.2023.1028643 (PMC9927024; doi:10.3389/fphys.2023.1028643)
Supplement: Supplementary file 12 [file DataSheet1.PDF]

Sample preparation for GC×GC-MS analysis

20 µL of an internal standard solution containing 11 standard compounds (50 µmol/L: 3-*O*-methyl-D-glucopyranose, D-pinitol, 5-bromo-2-hydroxyhippuric acid; 100 µmol/L: phenyl-β-D-glucopyranoside, hexylamine, *p*-chloro-L-phenylalanine, 5-chlorosalicylic acid, 2-chlorophenylacetic acid, 5-bromo-2,4-dihydroxybenzoic acid, 3,4-methylenedioxycinnamic acid; 150 µmol/L: 2-amino-6-chloropurine) were placed in screw-capped glass vials with 300 µL micro inserts. After thawing of the urine samples (QC and study samples) on ice, 40 µL were diluted with deionized water to normalize the samples to an osmolality of 60 mOsm/kg. Then, 40 µL of each diluted urine sample were pipetted into the screw-capped glass vials. Evaporation of samples was performed in a rotary vacuum concentrator (Savant SPD131DDA/RVT5105, Thermo Fisher Scientific, Massachusetts, USA; 1 h, 40 °C, <1 mbar), then 20 µL of methanol were added and again evaporated until dryness (20 min, 40 °C, <1 mbar). Afterwards, 15 µL of *O*-methoxylaminehydrochloride in pyridine (20 mg/mL; Chemos GmbH&Co.KG, Germany) were added and the methoximation conducted at 40 °C for 1 h at 1400 rpm. Trimethylsilylation was carried out by addition of 50 µL of MSTFA (Carl Roth GmbH&Co.KG, Germany) at 75 °C for 1.5 h and 800 rpm. To one daily QC sample, 10 µL of a retention index marker solution (C7-C28 saturated straight-chain fatty acid methyl esters 250 µmol/L in heptane) were added.

GC×GC-MS system and analysis

The untargeted GC×GC-MS system consisted of a gas chromatograph combined with a quadrupole mass spectrometer (Shimadzu GCMS QP2010 Ultra, Kyoto, Japan), an AOC-5000 Plus autosampler (Shimadzu, Kyoto, Japan) and a ZX-2 cryogenic loop-type modulator (ZOEX Corp, Houston, USA). The applied carrier gas was Helium 5.0 with an initial head pressure of 151.9 kPa, linear velocity was kept constant. Programmed temperature vaporization was performed with an OPTIC 4 (GL Sciences, Eindhoven, The Netherlands), the applied temperature program was as follows: 90°C → 60°C/s → 280°C (until end of run). Additionally, a split program was used: split ratio at the beginning was 1:3, after 1 min it was 1:100 and after 20 min 1:20 until end of run. 1 µL of the derivatized sample were injected into a deactivated, fritted glass liner with wool. The column of the first dimension was an RXI-5SilMS (Length: 39.5 m including 9.5 m of a guard column; internal diameter: 0.25 mm; film thickness: 0.25 µm; Restek, Bellefont, USA) and the column of the second dimension was a BPX50 (Length: 2.2 m, including a separation segment of 0.7 m; internal diameter: 0.15 mm; film thickness: 0.15 µm; Trajan Scientific, Ringwood, Australia). Both columns were connected using an Ultimate Union connector (Agilent Technologies GmbH, Waldbronn, Germany). The GC temperature ramp was: 90°C→2°C/min→100°C→3°C/min→140°C→2°C/min →180°C→2.5°C/min→200°C→3°C/min →240°C→5°C/min→280°C→8°C/min→300°C hold for 4.83 min), resulting in a total run time of 75 min. The temperature program of the modulator's hot jet was kept always at least 50°C above oven temperature in a stepwise manner, starting with 200 °C. The modulation period was 3 s and the hot jet pulse time 250 ms. The transferline was kept at 280 °C. Scan mode (*m/z* 60-550) was used with a scan speed of 20 000 u/s. The ion source temperature was 200 °C and EI spectra were recorded with 70 eV. Daily measurement order consisted of a daily QC sample with retention index marker, followed by two consecutive QC samples, three blocks of four study samples separated by two intermittent single QC samples, and finally two consecutive QC samples. Study samples were measured in a randomized way over all batches. Daily solvent blank samples were evaporated and only at the very last day of the measurement series derivatized and measured at the end of the whole measurement series. Liners and septa were changed weakly and the qMS was tuned weakly followed by six equilibration runs.

GC×GC-MS data processing

Data processing involves a number of different softwares (Shimadzu GCMSsolution, R modules, Microsoft Excel). The here applied workflow for a peaklet-based generic analysis of untargeted GC×GC-MS data was originally developed by Egert et al., however minor modifications have been made. In the first step, a textual peak list per run, including retention times (apex, start and end time), area, height, RI, annotation and mass spectra, was created using the GCMSsolution software (version 4.45). Second, a first global data matrix was created with a continuous peak list for all samples. In the next step, a data reduction was performed including several filters (e.g. for minimal peak height, noise and background peaks). Then, an alignment was performed for the combination of all matching modulations in one run and across all selected runs based on retention times in the first and second dimension and mass spectral similarity followed by a hierarchical cluster analysis for the mass spectra within each cluster. In case of clusters, that demanded to separate peaks with a high mass spectral similarity (e.g. isomers), a tentative separation was performed based on their peak height profile. After performance of peak merging (demodulation), a QC-based and batch-based drift correction was performed for those analyte clusters, where occurrence in QC samples was at least 80% in all measurement batches.

Egert, B., Weinert, C.H., and Kulling, S.E. (2015). A peaklet-based generic strategy for the untargeted analysis of comprehensive two-dimensional gas chromatography mass spectrometry data sets. *Journal of Chromatography A* 1405**,** 168-177. doi: 10.1016/j.chroma.2015.05.056.
