# Supplementary material for: Acute effects of moderate vs. vigorous endurance exercise on urinary metabolites in healthy, young, physically active men—A multi-platform metabolomics approach
Source: Front Physiol. 2023 Jan 30;14:1028643. doi: 10.3389/fphys.2023.1028643 (PMC9927024; doi:10.3389/fphys.2023.1028643)
Supplement: Supplementary file 13 [file Table5.DOCX]

|  |  |  | **Direction of Change** | | **Within Group FC (CME)** | | | **Within Group FC (CVE)** | | | **Between Groups FC**  **(CVE vs. CME)** | | |
| --- | --- | --- | --- | --- | --- | --- | --- | --- | --- | --- | --- | --- | --- |
| **Name** | **MSI** | **Specific Metabolic Pathway/Class** | **CME** | **CVE** | **U01/U00** | **U02/U00** | **U03/U00** | **U01/U00** | **U02/U00** | **U03/U00** | **U01(CVE)/U01(CME)** | **U02(CVE)/U02(CME)** | **U03(CVE)/U03(CME)** |
| **Amino acid metabolism** |  |  |  |  |  |  |  |  |  |  |  |  |  |
| 2-Ethyl-3-hydroxypropionic acid ^a^ | 2 | Isoleucine, leucine and valine metabolism | **-** | **Ʌ** |  |  |  | **3.5** | **2.4** |  |  |  |  |
| Tiglylglycine ^a^ | 1 | Isoleucine, leucine and valine metabolism | **-** | **-** |  |  | *0.5* | *2.5* |  |  | **2.3** | *2.5* |  |
| *N*-carbamyl-L-glutamic acid ^a^ | 1 | Glutamate metabolism | **-** | **Ʌ** |  |  |  | **2.5** | **2.2** |  | **3.2** | **2.9** |  |
| Alanine ^c^ | 2 | Alanine and aspartate metabolism | **-** | **Ʌ** | 1.4 |  |  | **2.4** | 1.4 |  | 1.5 |  |  |
| Alanine ^a^ | 1 | Alanine and aspartate metabolism | **-** | **Ʌ** |  |  |  | **2.3** |  |  |  |  |  |
| 4-Hydroxyphenylacetate ^a^ | 1 | Phenylalanine and tyrosine metabolism | **-** | **-** |  |  |  | *2.3* |  |  | **3.6** | *3.5* |  |
| Glutamic acid ^a^ | 1 | Glutamate metabolism | **-** | **-** |  |  | *0.5* | *2.2* |  |  | **3.2** |  |  |
| Taurine ^c^ | 2 | Cysteine, methionine and taurine metabolism | **Ʌ** | **Ʌ** | **1.8** | **1.7** | **1.7** | **2.0** | **2.4** | **3.2** | *0.6* |  |  |
| 4-Hydroxyphenylacetate ^c^ | 2 | Phenylalanine and tyrosine metabolism | **V** | **Ʌ** |  |  | **0.6** | **1.9** |  |  | **2.9** | **2.9** | **2.0** |
| Methylsuccinate ^c^ | 2 | Isoleucine, leucine and valine metabolism | **-** | **Ʌ** | 1.3 |  | 0.9 | **1.9** | 1.5 |  | 1.4 | 1.2 | 1.3 |
| 4-Hydroxyphenylpyruvic acid* ^a^ | 1 | Phenylalanine and tyrosine metabolism | **-** | **-** |  |  |  |  |  |  | **2.8** |  |  |
| Glycine ^c^ | 2 | Glycine, serine and threonine metabolism | **-** | **Ʌ** | 1.4 | 1.3 |  | **1.6** |  |  |  |  |  |
| Threonine ^c^ | 2 | Glycine, serine and threonine metabolism | **-** | **Ʌ** | 1.2 |  | 0.8 | **1.6** |  |  |  |  |  |
| Homovanillic acid ^a^ | 2 | Phenylalanine and tyrosine metabolism | **-** | **-** |  |  | 0.5 |  |  |  | **2.1** |  |  |
| 5-Hydroxy-3-indolacetic acid ^a^ | 2 | Tryptophan metabolism | **-** | **-** |  |  | *0.5* |  |  |  | **2.2** | 1.6 |  |
| *N,N*-Dimethylglycine ^b^ | 1 | Glycine, serine and threonine metabolism | **Ʌ** | **-** | 1.5 | **1.6** |  |  |  | 1.3 |  |  |  |
| Tyrosine ^c^ | 2 | Phenylalanine and tyrosine metabolism | **V** | **-** |  | 0.7 | **0.5** | 1.3 |  |  | 1.3 |  | 1.5 |
| Tryptophan ^a^ | 1 | Tryptophan metabolism | **V** | **-** |  |  | **0.5** |  |  |  |  |  |  |
| Creatine ^b^ | 1 | Creatine metabolism | **V** | **-** |  | **0.7** | **0.6** |  |  | 0.7 | *1.5* |  |  |
| Glycine-Proline ^a^ | 1 | Dipeptides | **V** | **-** | *0.3* | *0.2* | **0.2** |  |  | *0.4* |  | *2.1* |  |
| Hydroxyproline ^b^ | 1 | Arginine and proline metabolism; urea cycle | **V** | **V** | **0.6** | **0.5** | **0.6** |  | **0.4** | **0.4** |  |  | *0.6* |
| **Xenobiotic metabolites** |  |  |  |  |  |  |  |  |  |  |  |  |  |
| 2,3-Dihydroxy-2-methylpropanoic acid ^a^ | 2 | Unclassified | **-** | **Ʌ** |  | *2.3* | *2.1* | **3.1** | *2.6* | *2.2* |  |  |  |
| Ribonic acid-γ-lactone* ^a^ | 1 | Sugars, sugar substitutes and sugar derivatives | **Ʌ** | **Ʌ** | **2.2** |  |  | **2.9** |  |  |  |  |  |
| Isomaltose* ^a^ | 1 | Sugars, sugar substitutes and sugar derivatives | **-** | **Ʌ** |  |  |  | **2.8** |  |  |  |  |  |
|  |  |  |  |  |  |  |  |  |  |  |  |  |  |
|  |  |  |  |  |  |  |  |  |  |  |  |  |  |
|  |  |  | **Direction of Change** | | **Within Group FC (CME)** | | | **Within Group FC (CVE)** | | | **Between Groups FC**  **(CVE vs. CME)** | | |
| **Name** | **MSI** | **Specific Metabolic Pathway/Class** | **CME** | **CVE** | **U01/U00** | **U02/U00** | **U03/U00** | **U01/U00** | **U02/U00** | **U03/U00** | **U01(CVE)/**  **U01(CME)** | **U02(CVE)/**  **U02(CME)** | **U03(CVE)/**  **U03(CME)** |
| 2-Furoylglycine ^a^ | 1 | Food or plant constituents | **-** | **-** |  |  |  | *2.6* |  |  | *2.8* | **2.7** |  |
| Lactose ^a^ | 2 | Sugars, sugar substitutes and sugar derivatives | **-** | **-** |  |  |  | *2.1* |  |  | **2.1** |  |  |
| 5-Hydroxymethyl-2-furoic acid* ^a^ | 1 | Unclassified | **Ʌ** | **Ʌ** | **2.5** |  |  | **2.0** |  |  |  |  |  |
| *N*-Acetyl-D-mannosamine* ^a^ | 1 | Sugars, sugar substitutes and sugar derivatives | **-** | **-** |  |  |  |  |  |  | **2.8** |  |  |
| Gluconate ^c^ | 2 | Food or plant constituents | **-** | **Ʌ** |  |  |  | **1.7** |  |  | **1.6** |  |  |
| *trans*-Aconitate ^c^ | 2 | Food or plant constituents | **-** | **Ʌ** |  |  |  | **1.7** |  |  | 1.4 |  |  |
| *N*-Acetyl-D-gluco- and -mannosamine ^a^ | 1 | Sugars, sugar substitutes and sugar derivatives | **-** | **-** |  |  |  |  |  |  | **2.1** |  |  |
| Glucuronic acid ^a^ | 2 | Sugars, sugar substitutes and sugar derivatives | **-** | **-** |  |  |  |  |  |  | **2.1** | 1.4 |  |
| Resorcinol and Mesaconic acid ^a^ | 2 | Unclassified | **V** | **-** |  |  | **0.4** |  |  |  |  |  |  |
| Indol-3-carboxylic acid ^a^ | 2 | Food or plant constituents | **V** | **-** |  | **0.4** | **0.4** |  |  |  |  | 1.9 |  |
| *N*-Methylproline ^b^ | 1 | Food or plant constituents | **Ʌ** | **Ʌ** | *1.7* |  | **1.6** |  |  | **1.7** |  |  |  |
| Maltol* ^a^ | 2 | Sugars, sugar substitutes and sugar derivatives | **-** | **-** |  |  |  |  |  |  | **3.1** |  |  |
| 3,5-Dihydroxybenzoic acid ^a^ | 1 | Food or plant constituents | **V** | **-** | *0.5* | *0.4* | **0.4** |  |  | *0.3* | *3.3* | *2.0* |  |
| 7-Methylxanthine ^a^ | 1 | Xanthine metabolism | **-** | **-** | *0.4* | *0.3* | *0.3* |  |  | *0.2* | *2.7* | **2.5** |  |
| Mannitol and Sorbitol ^a^ | 1 | Sugars, sugar substitutes and sugar derivatives | **V** | **-** |  |  | **0.5** |  |  | *0.4* |  |  |  |
| Trigonelline ^b^ | 1 | Food or plant constituents | **V** | **V** | **0.6** | **0.5** | **0.5** | **0.6** | **0.5** | **0.5** |  |  |  |
| **Carbohydrate metabolism** |  |  |  |  |  |  |  |  |  |  |  |  |  |
| Fructose ^a^ | 1 | Fructose and mannose metabolism | **Ʌ** | **Ʌ** | **22.0** | *3.1* |  | **15.7** | *2.8* |  |  |  |  |
| Lactate ^c^ | 2 | Glucose and pyruvate metabolism | **-** | **Ʌ** | 1.4 |  | 1.0 | **2.8** | 1.3 |  | **2.0** |  |  |
| Lactate ^a^ | 1 | Glucose and pyruvate metabolism | **-** | **Ʌ** |  |  |  | **2.0** |  |  | 1.7 |  |  |
| Pyruvate ^c^ | 2 | Glucose and pyruvate metabolism | **-** | **Ʌ** |  |  |  | **2.0** |  |  | 1.5 | *1.6* |  |
| Mannose ^a^ | 2 | Fructose and mannose metabolism | **V** | **-** | *0.5* | *0.4* | **0.5** |  |  |  |  |  |  |
| **Mammalian-microbial cometabolism** |  |  |  |  |  |  |  |  |  |  |  |  |  |
| 4-Hydroxyphenyllactic acid ^a^ | 1 | Polyphenolic compounds metabolism | **-** | **-** |  |  |  | *2.4* |  |  | **2.9** | *3.0* | *2.0* |
| γ-Butyrobetaine ^b^ | 1 | Trimethylamines metabolism | **Ʌ** | **Ʌ** | **1.8** | 1.5 | **1.8** | **1.6** |  | **1.5** |  |  |  |
| Hippurate ^c^ | 2 | Polyphenolic compounds metabolism | **V** | **V** | **0.5** | **0.4** | **0.4** |  | **0.6** | **0.4** | **1.9** |  |  |
|  |  |  |  |  |  |  |  |  |  |  |  |  |  |
|  |  |  | **Direction of Change** | | **Within Group FC (CME)** | | | **Within Group FC (CVE)** | | | **Between Groups FC**  **(CVE vs. CME)** | | |
| **Name** | **MSI** | **Specific Metabolic Pathway/Class** | **CME** | **CVE** | **U01/U00** | **U02/U00** | **U03/U00** | **U01/U00** | **U02/U00** | **U03/U00** | **U01(CVE)/**  **U01(CME)** | **U02(CVE)/**  **U02(CME)** | **U03(CVE)/**  **U03(CME)** |
| Hippurate ^a^ | 1 | Polyphenolic compounds metabolism | **V** | **-** |  | **0.4** | **0.4** |  |  | *0.4* |  |  |  |
| 3-Indoxylsulfate ^c^ | 2 | Tryptophan metabolism | **-** | **V** | 0.8 | 0.7 | 0.7 |  | **0.6** | **0.5** | 1.4 |  |  |
| **Energy metabolism** |  |  |  |  |  |  |  |  |  |  |  |  |  |
| Malic acid ^a^ | 1 | Tricarboxylic acid cycle | **-** | **Ʌ** |  |  |  | **4.2** |  |  | 2.0 |  |  |
| *cis*-Aconitate ^a^ | 1 | Tricarboxylic acid cycle | **-** | **Ʌ** |  |  |  | **3.1** |  |  | **2.5** | 1.6 |  |
| *cis*-Aconitate ^c^ | 2 | Tricarboxylic acid cycle | **-** | **Ʌ** | 1.4 |  | 0.8 | **2.8** | 1.4 |  | **2.0** | 1.5 | 1.2 |
| Citrate ^c^ | 2 | Tricarboxylic acid cycle | **-** | **Ʌ** | 1.5 | 1.4 |  | **1.9** | **1.6** | **1.5** |  |  |  |
| **Nucleotide metabolism** |  |  |  |  |  |  |  |  |  |  |  |  |  |
| Hypoxanthine ^c^ | 2 | Purine metabolism | **V** | **ɅV** |  | **0.4** | **0.4** | **2.1** | *0.6* | **0.4** | **3.0** | **1.6** |  |
| Hypoxanthine ^a^ | 1 | Purine metabolism | **V** | **V** |  | **0.4** | **0.4** | 1.7 |  | **0.4** | **2.6** | 1.8 |  |
| Xanthine ^a^ | 2 | Purine metabolism | **V** | **-** |  | **0.4** | **0.3** |  |  | *0.4* | 2.0 | 2.0 |  |
| Uracil ^a^ | 1 | Pyrimidine metabolism | **V** | **V** |  | **0.4** | **0.4** |  | **0.5** | **0.4** |  |  |  |
| **Cofactors and vitamins metabolism** |  |  |  |  |  |  |  |  |  |  |  |  |  |
| Pantothenic acid ^a^ | 1 | Pantothenate and CoA biosynthesis | **-** | **-** |  |  |  | *3.1* |  |  | **3.1** | *2.1* |  |
| 1-Methylnicotinamide ^c^ | 2 | Nicotinate and nicotinamide metabolism | **V** | **V** | 0.7 | 0.7 | **0.6** | *0.6* | **0.5** | **0.6** | 1.1 |  |  |
| **Lipid metabolism** |  |  |  |  |  |  |  |  |  |  |  |  |  |
| Carnitine ^b^ | 1 | Carnitine metabolism | **Ʌ** | **Ʌ** | **3.0** | *1.5* | *1.5* | **2.3** | *1.6* | *2.4* |  |  | *2.2* |
| **Unclassified** |  |  |  |  |  |  |  |  |  |  |  |  |  |
| Sugar-like 2 ^a^ | 3 | - | **V** | **-** |  |  | **0.4** | *2.2* |  |  |  |  |  |
| Similar to 1,5-Anhydrosorbitol ^a^ | 3 | - | **-** | **-** |  |  |  |  |  |  | **2.7** |  |  |
| C6-Sugar acid lactone* ^a^ | 3 | - | **-** | **-** |  |  |  |  |  |  | **2.0** |  |  |
| Sugar-like 4 ^a^ | 3 | - | **-** | **-** |  |  |  |  |  |  | **2.3** |  |  |
| Deoxymethyl-Sugar acid (C5) ^a^ | 3 | - | **-** | **-** |  |  |  |  |  |  | **2.4** | 1.7 |  |
| Amino derivative ^a^ | 3 | - | **-** | **-** |  |  |  |  |  |  | **2.7** |  |  |
| *N*-Acetyl-D-hexosaminitol ^a^ | 3 | - | **V** | **-** |  | *0.4* | **0.5** |  |  |  |  |  |  |
| C5-Sugar acid 1 ^a^ | 3 | - | **V** | **-** |  |  | **0.5** |  |  |  | **2.1** |  |  |
|  |  |  |  |  |  |  |  |  |  |  |  |  |  |
|  |  |  |  |  |  |  |  |  |  |  |  |  |  |
|  |  |  | **Direction of Change** | | **Within Group FC (CME)** | | | **Within Group FC (CVE)** | | | **Between Groups FC**  **(CVE vs. CME)** | | |
| **Name** | **MSI** | **Specific Metabolic Pathway/Class** | **CME** | **CVE** | **U01/U00** | **U02/U00** | **U03/U00** | **U01/U00** | **U02/U00** | **U03/U00** | **U01(CVE)/**  **U01(CME)** | **U02(CVE)/**  **U02(CME)** | **U03(CVE)/**  **U03(CME)** |
| C5-Sugar acid 2 ^a^ | 3 | - | **-** | **-** |  |  |  |  |  |  | **2.6** |  |  |
| Amino acid-like* ^a^ | 3 | - | **-** | **-** |  |  |  |  |  |  | **2.3** |  |  |
| Sugar-like 1 ^a^ | 3 | - | **V** | **-** |  | *0.5* | **0.4** |  |  |  |  |  |  |
| Similar to α-Hydroxyglutaric acid ^a^ | 3 | - | **-** | **-** |  |  |  |  |  |  | **2.0** |  |  |
| 3-Deoxyhexonic acid ^a^ | 3 | - | **-** | **-** |  | *0.5* | *0.5* |  |  |  | **2.4** |  |  |
| Similar to 3',5'-Dihydroxyflavone ^a^ | 3 | - | **V** | **-** | *0.5* | **0.3** | **0.3** |  |  | *0.4* |  | *3.1* |  |
| Sugar-like 3 ^a^ | 3 | - | **V** | **V** | *0.2* | **0.1** | **0.0** |  | **0.2** | **0.1** |  |  |  |
| **Unknown** |  |  |  |  |  |  |  |  |  |  |  |  |  |
| U0456 ^a^ | 4 | - | **Ʌ** | **Ʌ** | **3.6** | *3.5* | *2.2* | **5.8** | **4.3** | **2.6** | **2.1** |  |  |
| U0829 ^a^ | 4 | - | **Ʌ** | **Ʌ** | **2.6** | *2.2* |  | **4.0** | **3.5** | *2.2* | 1.8 |  |  |
| U0262 ^a^ | 4 | - | **-** | **Ʌ** | *2.7* | *2.6* | *2.6* | **3.9** | **3.8** | **3.1** |  |  |  |
| U0876 ^a^ | 4 | - | **-** | **Ʌ** |  |  |  | **3.8** | *2.2* |  |  |  |  |
| U1091 ^a^ | 4 | - | **-** | **-** |  |  |  | *3.5* |  |  | **3.4** |  | *2.3* |
| U1083 ^a^ | 4 | - | **-** | **-** | *0.4* | *0.4* | *0.5* | *2.3* |  |  | **3.4** |  |  |
| U0569 ^a^ | 4 | - | **-** | **-** |  | *0.4* | *0.4* | *2.2* |  |  | **2.2** | **2.3** |  |
| U0603 ^a^ | 4 | - | **-** | **-** |  |  |  |  |  |  | **2.2** |  |  |
| U0928* ^a^ | 4 | - | **-** | **-** |  |  |  |  |  |  | **2.7** |  |  |
| U0231 ^a^ | 4 | - | **-** | **-** |  |  |  |  |  |  | **2.2** |  |  |
| U0775 ^a^ | 4 | - | **-** | **-** |  | *0.4* | *0.3* |  | *0.4* | *0.3* | **2.8** |  |  |
| U0688 ^a^ | 4 | - | **-** | **-** |  |  |  |  |  |  | **2.4** |  |  |
| U0274 ^a^ | 4 | - | **-** | **-** |  | *0.5* | *0.4* |  |  | *0.5* | **2.3** |  |  |
| U0430* ^a^ | 4 | - | **-** | **-** |  |  |  |  |  |  | **2.4** |  |  |
| U0740 ^a^ | 4 | - | **-** | **-** |  |  |  |  |  |  | **2.1** |  |  |
| U0956 ^a^ | 4 | - | **V** | **-** |  | *0.5* | **0.4** |  |  |  |  |  |  |
| U0339 ^a^ | 4 | - | **-** | **-** |  |  |  |  |  |  |  | *2.1* | **0.5** |
| U1163 ^a^ | 4 | - | **V** | **-** |  | *0.3* | **0.3** |  |  | *0.5* | **2.6** | *4.2* | *2.2* |
|  |  |  |  |  |  |  |  |  |  |  |  |  |  |
|  |  |  | **Direction of Change** | | **Within Group FC (CME)** | | | **Within Group FC (CVE)** | | | **Between Groups FC**  **(CVE vs. CME)** | | |
| **Name** | **MSI** | **Specific Metabolic Pathway/Class** | **CME** | **CVE** | **U01/U00** | **U02/U00** | **U03/U00** | **U01/U00** | **U02/U00** | **U03/U00** | **U01(CVE)/**  **U01(CME)** | **U02(CVE)/**  **U02(CME)** | **U03(CVE)/**  **U03(CME)** |
| U0629 ^a^ | 4 | - | **-** | **-** |  |  |  |  |  |  | **2.6** |  |  |
| U0673 ^a^ | 4 | - | **-** | **-** |  |  |  |  |  |  | **2.3** | 1.5 |  |
| U0737 ^a^ | 4 | - | **-** | **-** |  |  | *0.4* |  |  | *0.5* | **2.2** |  |  |
| U0533 ^a^ | 4 | - | **-** | **-** |  |  |  |  |  |  | **2.1** |  |  |
| U0529 ^a^ | 4 | - | **V** | **-** |  | **0.4** | **0.4** |  |  |  |  |  |  |
| U0794 ^a^ | 4 | - | **-** | **-** |  |  |  |  |  |  | **2.4** |  |  |
| U0468 ^a^ | 4 | - | **-** | **-** |  | *0.3* |  |  |  | *0.4* | **2.4** | *2.0* |  |
| U0089* ^a^ | 4 | - | **-** | **-** |  |  |  |  |  |  | **2.3** |  |  |
| U1069 ^a^ | 4 | - | **V** | **V** | *0.5* | **0.3** | **0.3** |  | *0.4* | **0.2** |  |  |  |
| U0598 ^a^ | 4 | - | **V** | **V** | **0.4** | **0.4** | **0.4** |  | *0.3* | **0.3** | **2.3** |  |  |

Metabolites are sorted by most relevant major metabolic pathways and decreasing median FCs for U01/U00 (CVE). Bold: relevant median FC >2.0 or <0.5 (GC×GC-MS data) or >1.5 or <0.$\overline{6}$(LC-MS/NMR data) and FDR-corrected p-value <0.05 or interesting spectral features based on heuristic/visual approach (*); italics: relevant FC but FDR-corrected p-value ≥0.05; normal font: irrelevant FC but FDR-corrected p-value <0.05. FDR-corrected p-values were evaluated based on values rounded to four decimal places; FCs were evaluated based on values rounded to two decimal places. ^a^: GC×GC-MS-detected; ^b^: LC-MS-detected; ^c^: NMR-detected; V: decrease; Ʌ: increase; -: no change. The direction of change of urinary metabolites was evaluated based on bold values, i.e., if both FCs and FDR-corrected p-values were relevant. CME: continuous moderate exercise; CVE: continuous vigorous exercise; FC: fold change; MSI: Metabolomics Standards Initiative; U: unknown analyte; U00: fasting spot urine, pre-exercise; U01: urine produced during exercise trials until 45 minutes post-exercise; U02: urine produced from 45 to 105 minutes post-exercise; U03: urine produced from 105 to 195 minutes post-exercise.
