# Supplementary material for: Acute effects of moderate vs. vigorous endurance exercise on urinary metabolites in healthy, young, physically active men—A multi-platform metabolomics approach
Source: Front Physiol. 2023 Jan 30;14:1028643. doi: 10.3389/fphys.2023.1028643 (PMC9927024; doi:10.3389/fphys.2023.1028643)
Supplement: Supplementary file 15 [file Table6.DOCX]

|  | **Between Groups FC (CVE vs. CME)** | | | |
| --- | --- | --- | --- | --- |
| **Name** | **U01(CVE)/U01(CME)** | **U02(CVE)/U02(CME)** | **U03(CVE)/U03(CME)** | **U01+U02+U03(CVE)/U01+U02+U03(CME)** |
| Hypoxanthine | **2.6** | *1.5* |  | **1.9** |
| 4-Hydroxyphenylacetate | *1.9* | *2.3* | *2.1* | *2.5* |
| Lactate | *1.6* |  |  |  |
| Hippurate | *1.5* |  |  |  |
| Sarcosine |  | *1.5* | *2.3* | *1.6* |
| π-Methylhistidine |  | *1.5* |  |  |
| Hydroxyproline |  | *0.7* | *0.6* |  |
| y-Butyrobetaine | *0.7* |  |  |  |
| Citrate | *0.6* |  |  |  |
| Carnitine | *0.6* |  | *1.5* |  |
| Histidine | *0.6* |  |  |  |
| Dimethylsulfone | *0.6* |  |  |  |
| N,N-Dimethylglycine | *0.6* |  |  |  |
| Stachydrine | *0.5* | *0.6* |  |  |
| N-Methylproline | *0.5* |  |  |  |
| Taurine | *0.3* |  |  |  |

Metabolites are sorted by decreasing median FCs for U01(CVE)/U01(CME) ratio. Bold: relevant median FC >1.5 or <0.$\overline{6}$and FDR-corrected p-value <0.05; italics: relevant FC but FDR-corrected p-value ≥0.05. FDR-corrected p-values were evaluated based on values rounded to four decimal places; FCs were evaluated based on values rounded to two decimal places.
